# The nuclear genome of *Rhazya stricta* and the evolution of alkaloid diversity in a medically relevant clade of Apocynaceae

**DOI:** 10.1038/srep33782

**Published:** 2016-09-22

**Authors:** Jamal S. M. Sabir, Robert K. Jansen, Dhivya Arasappan, Virginie Calderon, Emmanuel Noutahi, Chunfang Zheng, Seongjun Park, Meshaal J. Sabir, Mohammed N. Baeshen, Nahid H. Hajrah, Mohammad A. Khiyami, Nabih A. Baeshen, Abdullah Y. Obaid, Abdulrahman L. Al-Malki, David Sankoff, Nadia El-Mabrouk, Tracey A. Ruhlman

**Affiliations:** 1 Biotechnology Research Group, Department of Biological Sciences, Faculty of Science, King Abdulaziz University, Jeddah 21589, Saudi Arabia; 2 Department of Integrative Biology, University of Texas at Austin, Austin, 78712 Texas, USA; 3 Département d’informatique et de recherche opérationnelle, Université de Montréal, CP 6128 succ Centre-Ville, Montréal, H3C 3J7 Québec, Canada; 4 Department of Mathematics and Statistics, University of Ottawa, 585 King Edward Ave., Ottawa, K1N 6N5 Ontario Canada; 5 Department of Biological Sciences, Faculty of Science, University of Jeddah, Jeddah, Saudi Arabia; 6 King Abdulaziz City for Science and Technology, Riyadh 11442, Saudi Arabia; 7 Department of Biological Sciences, Faculty of Science, King Abdulaziz University, Jeddah 21589, Saudi Arabia; 8 Department of Chemistry, Faculty of Science, King Abdulaziz University, Jeddah 21589, Saudi Arabia; 9 Department of Biochemistry, Faculty of Science, King Abdulaziz University, Jeddah 21589, Saudi Arabia

## Abstract

Alkaloid accumulation in plants is activated in response to stress, is limited in distribution and specific alkaloid repertoires are variable across taxa. Rauvolfioideae (Apocynaceae, Gentianales) represents a major center of structural expansion in the monoterpenoid indole alkaloids (MIAs) yielding thousands of unique molecules including highly valuable chemotherapeutics. The paucity of genome-level data for Apocynaceae precludes a deeper understanding of MIA pathway evolution hindering the elucidation of remaining pathway enzymes and the improvement of MIA availability *in planta* or *in vitro*. We sequenced the nuclear genome of *Rhazya stricta* (Apocynaceae, Rauvolfioideae) and present this high quality assembly in comparison with that of coffee (Rubiaceae, *Coffea canephora*, Gentianales) and others to investigate the evolution of genome-scale features. The annotated *Rhazya* genome was used to develop the community resource, RhaCyc, a metabolic pathway database. Gene family trees were constructed to identify homologs of MIA pathway genes and to examine their evolutionary history. We found that, unlike *Coffea*, the *Rhazya* lineage has experienced many structural rearrangements. Gene tree analyses suggest recent, lineage-specific expansion and diversification among homologs encoding MIA pathway genes in Gentianales and provide candidate sequences with the potential to close gaps in characterized pathways and support prospecting for new MIA production avenues.

The power of genomics to illuminate evolutionary hypotheses lies in the availability of sequences from both early and late branching lineages for comparative analyses. Alkaloid accumulation in plant tissues is triggered in response to the range of stress encountered by plants; for humanity the result is a pharmacopeia ranging from caffeine to heroin to some of the most important chemotherapeutic and hypertensive treatment drugs available today^1^. Given that plant metabolic networks have followed a descent with modification pattern^2^ and the unique and variable character of monoterpenoid indole alkaloid (MIA) production throughout the angiosperm order Gentianales, genome-scale comparative analyses within the order relative to more distant lineages could provide insight on the genetic factors involved in the evolution of this highly valuable aspect of plant secondary metabolism.

Alkaloid production in plants is limited in distribution and the specific alkaloid repertoire is variable across taxa. With the exception of the MIA quinolone derivatives, i.e. camptothecin, Gentianales are the exclusive plant producers of highly valuable MIA compounds, including the bisindole topoisomerase inhibitors vincristine and vinblastine. Regardless of their final form, more than 2500 identified MIAs begin with the condensation of tryptamine and secologanin to form strictosidine, the first committed molecule in the MIA pathway. The classification of MIAs into three broad groups was based on the arrangement of the fragmented cyclic monoterpene (secologanin), Iboga (catharanthine), Corynanthe-strychnos (ajmalicine) and Aspidosperma (stemmadenine). While all families of the order contain representatives that can accumulate MIA species with the Corynathe (type I) skeleton, Rauvolfioideae, a subfamily of Apocynaceae, represents a major center of structural expansion encompassing the diversity of skeletal arrangements and an astonishing number of downstream modifications yielding thousands of unique molecules^3^.

*Rhazya stricta* Decne. (2n = 22; Apocynaceae, Rauvolfioideae) is an evergreen shrub abundant across Western and South Asia and is well adapted to harsh conditions^4^. Like other Rauvolfioideae, *Rhazya* accumulates MIAs. In fact, strictosidine was first isolated from *R. stricta* in 1968^5^. However recent study has focused mainly on *Catharanthus roseus* (L.) G. Don and, to a lesser extent, *Rauvolfia serpentina* (L.) Benth. ex Kurz and a handful of others in the family. There is an extensive literature describing chemical synthetic approaches to generate the kind of molecules produced by these species as, at least in the systems that have been studied, *in vivo* production is far below human demand^6^. Historical technological challenges to high quality assembly of complete nuclear genomes have resulted in fragmented drafts of limited use due to the exclusion of repetitive sequences and the lack of information on genome structure. Furthermore, the paucity of genome level data for Apocynaceae precludes a deeper understanding of MIA pathway evolution hindering the elucidation of remaining pathway enzymes and the potential to improve MIA availability *in planta* or *in vitro*. To address this, we sequenced the nuclear genome of *Rhazya stricta* and present this high quality assembly in comparison with that of coffee (Rubiaceae, *Coffea canephora* Pierre ex A. Froehner, Gentianales) and others to investigate the evolution of genome-scale features. Using genomic and transcriptomic sequences from Rauvolfioideae along with species in the order Gentianales and beyond, we identified homologs of characterized MIA pathway genes revealing the lineage specific nature of their evolution.

## Results and Discussion

We sequenced three Illumina libraries yielding nearly 400 million reads to generate approximately 112X coverage of the *R. stricta* ~274 Mb genome. To achieve the highest quality draft with minimal fragmentation we added PacBio sequencing, (>550K reads; 10X coverage), and isolated nuclei for optical mapping (Supplementary Table S1). The final assembly contains 980 scaffolds with a maximum scaffold length of 16.4 Mb and N50 >5.5 Mb. Scaffolds of greater than 200 Kb (113) comprise 93% of the genome sequence (Supplementary Table S2, Supplementary Table S3). Comparison to assembly statistics reported for three recently published nuclear genomes demonstrates the high quality of the *Rhazya* draft (Supplementary Table S4). A comprehensive listing of statistics for 34 non-model plant nuclear genomes was recently reported by Unamaba *et al*.^7^.

The transcriptome-guided annotation of the *R. stricta* genome identified 21,164 protein-coding genes (Supplementary Table S5), 60% of which were assigned UniProt identifiers. Comparison to a set of core eukaryotic sequences (CEGMA)^8^ demonstrated that the *R. stricta* assembly captured 98% of the expected genes. The genome contains 16% transposable elements, of which 88% are long terminal repeat (LTR) retrotransposons comprising predominantly Copia and Gypsy superfamily members. Our list of repeated elements is not exhaustive and the actual value for repeat content is likely higher (Supplementary Table S6). All functionally annotated transcripts, as described in the methods section, were assigned gene names, putative functions, gene ontology (GO) terms and enzyme codes by homology search (BLAST) against the UniProt database.

A metabolic pathway database, RhaCyc, was created for *Rhazya* using the PathoLogic component of Pathway Tools (http://bioinformatics.ai.sri.com/ptools/). This program inferred metabolic pathways by comparing the annotated *Rhazya* genes to the MetaCyc 2.0 reference pathway database (http://www.metacyc.org). The RhaCyc database contains information on 3380 enzymes, 2828 compounds, and 458 pathways and is available as a community resource (http://rhacyc.icmb.utexas.edu:1555/). Links to UniProt identifiers and GO terms are embedded with each annotated sequence in RhaCyc.

**Figure 1:**
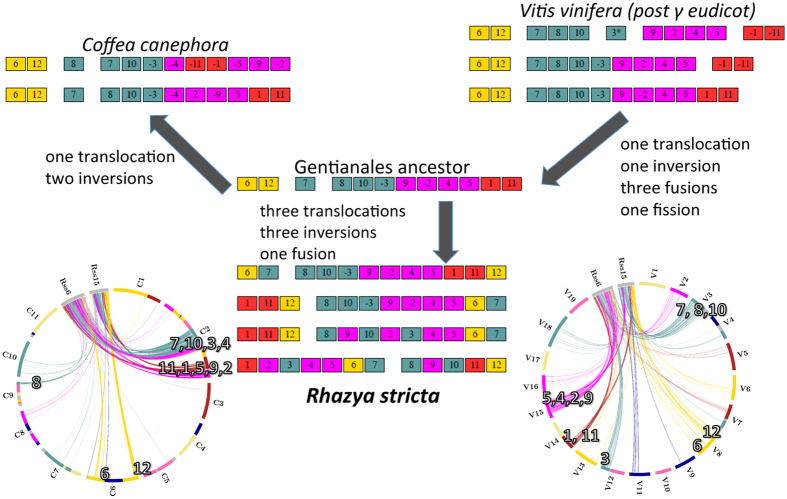
Divergent structural evolution in *Rhazya stricta*. Genome level rearrangements leading from the ancestral core eudicot, represented by *Vitis* (upper right hand corner) to *Rhazya* and *Coffea*. Each linear arrangement represents successive steps from the eudicot ancestor toward the Gentianales ancestor. An arrow leads from the last of these stages to the relevant portion of the Gentianales ancestor. From this ancestor, two arrows indicate the divergence of *Coffea* and *Rhazya*, and successive rearrangements within each lineage are again indicated by linear arrangements of colored blocks. Inferred rearrangements between lineages are given at the arrows. Extant genomes carry the species name. The circular diagrams at the lower left and right represent the current orthologies between *Rhazya* superscaffolds 6 and 15, and *Coffea* and *Vitis*, respectively. Syntenic blocks are colored according to the 21 ancestral core eudicot chromosomes^33^ and do not represent biologically significant units.

The high quality draft of the *Coffea canephora* nuclear genome^9^ was used to examine structural changes between the two Gentianales families and relative to the ancestral post-γ eudicot arrangement as represented by *Vitis*^10^ (Fig. 1; Supplementary Fig. S1). As in *Coffea*, there was no evidence to suggest a subsequent whole-genome polyploidization in the *Rhazya* lineage. Comparison of adjacencies within each of the *Rhazya* scaffolds derived from different ancestral chromosomes and those within the *Coffea* chromosomes allowed us to identify most of the major gene order changes in the extant genomes. While the remaining *Rhazya* scaffolds each tend to reflect a part of only a single ancestral chromosome, they may contain some smaller rearrangements that went undetected. With the available data we detected limited rearrangement between the core eudicot ancestor and the Gentianales ancestor, and between the latter and *Coffea*. In the *Rhazya* lineage, however, many more structural rearrangements were inferred.

**Figure 2:**
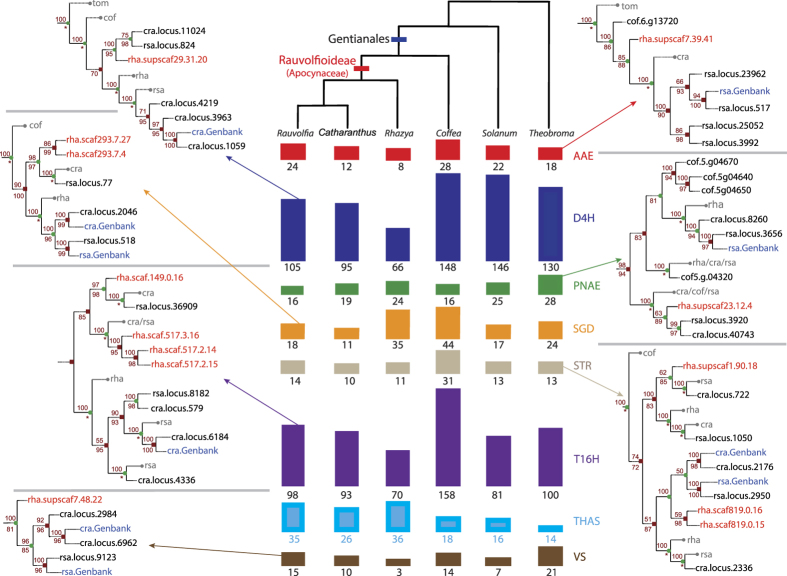
Expansion, loss and duplication of selected monoterpenoid indole alkaloid (MIA) sequences across Gentianales. The cladogram (top, center) depicts relationships among six eudicots based on the Angiosperm Phylogeny Group (http://www.mobot.org/MOBOT/research/APweb/). Histograms below each species indicate the number of sequences returned in BLAST searches to identify homologs for each of the eight gene families. The maximum likelihood trees along the left and right show subclades of reconciled gene phylogenies that include known homologs in either *Catharanthus* (cra.Genbank) or *Rauvolfia* (rsa.Genbank) for seven of the eight MIA genes (THAS is shown in Fig. 3). Complete reconciled phylogenies for each of the eight sequences are shown in Supplementary Fig. S3. cra, *Catharanthus roseus*; cof, *Coffea canephora*; rha, *Rhazya stricta*; tom, *Solanum lycopersicum*; rsa, *Rauvolfia serpentina*. Blue font indicates GenBank sequences and red font indicates *Rhazya stricta* sequences. Red square = gene duplication; green circle = speciation event; gray circles = gene loss. Values above branches indicate bootstrap support for the clade, numbers below nodes give bootstrap support for the event (duplication or speciation) at the node. Bootstrap values less than 50 are not shown; inferred loss events are indicated with an asterisk and do not have associated bootstrap values.

**Figure 3:**
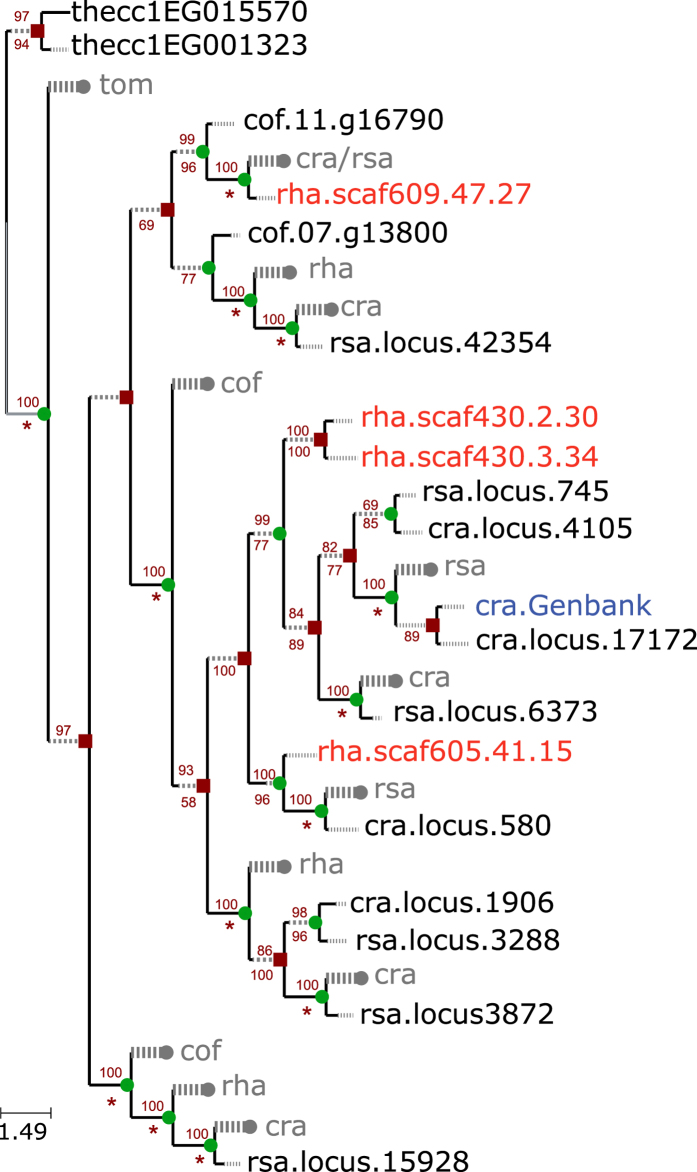
Duplication and loss of Tetrahydroalstonine synthase (THAS) across six angiosperms based on gene tree/species tree reconciliation. Internal and terminal branches in black reflect the amount of change along branch; dashed gray branch extensions are present for branches whose lengths were too short to enable uniform representation with branch support and length information. cra, *Catharanthus roseus*; cof, *Coffea canephora*; rha, *Rhazya stricta*; rsa, *Rauvolfia serpentina*; tom, *Solanum lycopersicum*; the, *Theobroma cacao*. Species indicated in blue font were downloaded from GenBank and those in red are from *Rhazya stricta*. Red square = gene duplication; green circle = speciation event; wide gray dashed line ending with a circle = gene loss. Values above branches indicate bootstrap support for the clade, numbers below nodes give bootstrap support for the event (duplication or speciation) at the node. Bootstrap values less than 50 are not shown; inferred loss events are indicated with an asterisk and do not have associated bootstrap values. Scale bar represents number of amino acid substitutions per site. The maximum likelihood (ML) tree shown is the tree with the best ML score.

To explore the evolution of MIA pathway genes we assembled a translated subject database containing transcriptomic and genomic data for sequences from *Rhazya* and five additional species: two Rauvolfioideae (C. *roseus*, *R. serpentina*), *C*. *canephora* (Rubiaceae), a non-Gentianales asterid (*Solanum lycopersicum* L.) and an alkaloid producing rosid (*Theobroma cacao* L. cv Matina). For homolog identification we queried the database using previously characterized MIA protein sequences from *C. roseus* and *R. serpentina*, which produce alkaloids currently employed in cancer chemotherapy and as antiarrhythmic drugs, respectively. Putative homologs for each protein were aligned and used to generate phylogenies (Supplementary Fig. S2) for 21 genes (Supplementary Table S7). Of these, eight trees were reduced and restricted to the relevant clades as described in methods and reconciled with the species tree, depicting the history of duplication and loss for each (Figs 2 and 3, Supplementary Fig. S3, Supplementary Table S8).

Generally, the families that include the eight characterized MIA genes underwent expansion as is seen in other gene families whose members participate in secondary metabolism^2^ (Supplementary Table S8). Where genome level data, and therefore information about genetic loci, were available gene family expansion appears to have been facilitated by localized and/or tandem duplications. The two Gentianales taxa, *Coffea* and *Rhazya*, each contain proximally arrayed homologs. Within each species some homologs are grouped in distinct clades while others are dispersed across the phylogeny suggesting different evolutionary trajectories for these sequences. One example can be seen in *Coffea.* The full tree generated from sequences extracted using *C. roseus* Strictosidine β-glucosidase (SGD) as a query contains 157 β-glucosidase superfamily members (Supplementary Fig. S2), 17 of which are proximally arrayed on *Coffea* chromosome two. Of those, eight chromosome two sequences were retained in the reconciled subtree (Supplementary Fig. S3). The duplications giving rise to five glucosidases (Cc02_g27160, g27180, g27190, g27230, g27290; highlighted in brown) likely occurred prior to the divergence of Gentianales while more recent duplications gave rise to three homologs that form a clade containing *Coffea* sequences exclusively (Cc02_g30420, g30490, g30540; highlighted in beige).

For SGD, as for all of the experimentally confirmed MIA sequences analyzed, the *C. roseus* and/or *R. serpentina* sequences downloaded from Genbank were in derived positions in the phylograms. The clades that contained the known (query) sequences were restricted to the Rauvolfioideae, however some clades also included coffee sequences following the pattern of diversification within the order and reflecting the evolution of MIA diversity and complexity.

Our analyses placed two *Rhazya* sequences in the clade adjacent to the GenBank SGD sequences, along with a single sequence extracted from the *Rauvolfia* transcriptome (Fig. 2, Supplementary Fig. S2 and S3). However, SGD was not identified and annotated in the *Rhazya* genome. Looking more closely at these homologs, we aligned the *Rhazya* and *Rauvolfia* sequences within and between the clades. The *Rauvolfia* sequence grouping with the two *Rhazya* sequences was identified as Raucaffricine-O-β-D-glucosidase (RG; EC 3.2.1.125). The two *Rhazya* sequences and RG were 76% and 77% identical whereas identities to *Rauvolfia* SGD were 58% and 57% at the amino acid level.

Thus it appears that the *R. stricta* genome may not encode SGD, nonetheless our own mass spectrometry analysis (Supplementary Table S9) along with decades of reports demonstrate the production of MIAs that derive from the reactive strictosidine aglycone, a product of SGD activity. The presence of a close homolog of RG, however, suggests another route to downstream intermediates and ultimately MIA end products. Raucaffricine, the major substrate of RG, is hydrolyzed to vomilenin, an intermediate in the ajmaline pathway^11^. While SGD is unable to hydrolyze raucaffricine, RG can catalyze the conversion of strictosidine^12^, and may fill that role in *R. stricta* cells. Identification and annotation of the SGD gene in a low quality nuclear genome draft from *C. roseus* proved challenging^13^, however, given the depth and completeness of our assembly it is unlikely that a *Rhazya* SGD sequence went undetected. Recently a similar approach used to search for MIA pathway genes in *Amsonia hubrichtii* (Apocynaceae, Rauvolfioideae) failed to detect a high identity homolog of the *C. roseus* SGD. A single transcript was designated as encoding a putative SGD with 57% amino acid identity to the *Catharanthus* sequence^14^, suggesting that another β-glucosidase may fill the role of SGD in this species.

The incredible diversity of MIAs in the Rauvolfioidae is facilitated through the hydrolysis of strictosidine to its aglycone, which may then be converted into different structural classes and elaborated by myriad reactions, many of which have been characterized, at least for *C. roseus*. However the enzymatic activities responsible for directing which structural class of MIA will ultimately derive from the aglycone moiety were largely unknown, until recently. THAS activity (Tetrahydroalstonine synthase) converts the strictosidine aglycone to tetrahydroalstonine, the gateway molecule to other heteroyohimbine type alkaloids such as cathenamine^15^,^16^. We queried our database with the THAS amino acid sequence, extracted homologs for gene phylogenies, and produced trimmed, reconciled trees. Much like the other MIA pathway genes examined, THAS family members showed a pattern of lineage specific expansion in the Rauvolfioideae and included two sequences from *R. stricta* in the reduced tree (Fig. 3, Supplementary Fig. S2). While the two proximal sequences had only 60% amino acid identity to each other, their identities to the characterized *C. roseus* THAS was greater, at 71% and 75%.

Access to the high quality draft of the *Coffea* nuclear genome, along with drafts representing other lineages outside of Gentianales, has allowed us to use the *Rhazya* nuclear genome comparatively to evaluate lineage specific evolution of MIA pathway sequences. However, limiting these analyses to a single representative from each lineage necessarily limits the inferences that can be drawn from comparative study. The highly fragmented draft of the *C. roseus* genome^13^, while providing a useful resource for certain investigations, was not suitable for inclusion in the gene order comparisons. A more recent approach using single platform nuclear genome sequencing with PacBio generated a high quality draft for the grass genus *Oropetum*^17^. As sequencing technologies continue to improve in terms of both data quality and expense, less fragmented draft genomes for other Apocynaceae should become more tenable for future analyses.

Nonetheless, the gene phylogeny analyses facilitated by the *Rhazya* sequences combined with available data indicate lineage specific expansion resulting in distinct sets of MIA enzyme homologs in the Rauvolfioideae as well as in the Gentianales. The protein sequences clustering with characterized MIA homologs provide an evolutionary insight that could guide full characterization of MIA pathways in related species. One example may be found among the homologs of *C. roseus* Reticuline oxidase (RO, EC 1.21.3.3). This cytochrome P450 flavoprotein is a member of the BBE (Berberine bridge enzyme) family (pfam 08031) that catalyzes carbon-carbon bond formation via oxidative cyclization. An enzyme possessing such activity has been characterized in extracts from *R. serpentina*, but as yet the gene for SBE (Sarpagan bridge enzyme)^18^ remains unknown. The inclusion of several *Rauvolfia* and *Rhazya* sequences in the clade containing *C. roseus* RO (Supplementary Fig. S2m) may indicate an avenue for future studies seeking to identify elusive MIA pathway genes.

As more high quality nuclear genome drafts become available we will be able to address genome scale hypotheses of evolution. The rearrangements in *Rhazya* but not *Coffea*, relative to their shared ancestor may suggest that genome level dynamics played a role in the evolution of chemical diversity in the Rauvolfioideae. A critical evaluation of this question would require more than a single representative from each lineage to draw meaningful conclusions.

## Materials and Methods

### Plant material, DNA and RNA isolation

*Rhazya stricta* seeds were obtained from natural populations collected in the Makkah Province, Saudi Arabia. Seeds were soaked in water overnight at 37 °C then transferred to Profile^®^ Field & Fairway™ inorganic ceramic particles (Buffalo Grove, IL) in a growth chamber (16 h light, 8 h dark 38 °C) for germination. Young leaves were flash frozen in liquid nitrogen for DNA and RNA isolation and stored at −80 °C. Isolation of *R. stricta* nucleic acids was described in Park *et al*.^19^. Young leaves of *R*. *stricta* were collected at the same location and shipped to INDRAS Private Limited (Hyderabad India) for mass spectrometry analysis of monoterpene indole alkaloid content.

For optical mapping, a megabase DNA isolation protocol was used to obtain intact nuclei from *R. stricta* for embedding in agarose. The protocol was downloaded from the Arizona Genomics Institute at the University of Arizona (http://www.genome.arizona.edu/modules/publisher/item.php?itemid=24).

### Genome Sequencing

Illumina (San Diego, CA) DNA libraries were sequenced on the Illumina HiSeq 2500 at the Genome Sequencing and Analysis Facility at the University of Texas at Austin (GSAF). Three different library types were prepared, as required by the AllPaths-LG assembler (http://www.broadinstitute.org/software/allpaths-lg/blog/)^20^, an overlapping library with size range of 160–220 base pairs (bp), a mate-pair library ranging from 2–3 kilobases (kb), and a 6 kb mate-pair library. In addition, eight single molecule, real time (SMRT) cells of single-end PacBio RS reads were generated from a 10 kb library at the Interdisciplinary Center for Biotechnology Research, University of Florida, Gainesville. Supplementary Table S1 provides information about the data generated.

### Transcriptome Sequencing

Total RNA isolation, library construction, and Illumina sequencing were performed according to Zhang *et al*.^21^. Duplex specific nuclease normalization (Evrogen, Moscow, Russia) of the RNA samples, Illumina RNAseq library construction, and sequencing were carried out at GSAF. Duplex specific nuclease normalization (Evrogen, Moscow, Russia) of the RNA samples, Illumina RNAseq library construction and sequencing were carried out at the GSAF. Raw read output from *R. stricta* RNAseq was deposited in the Small Read Archive (SRA) at the NCBI (accession number SRR1151604)^18^. The RNASeq data was assembled using Trinity (v2013_08_14)^22^. The assembly was annotated using Trinotate (https://trinotate.github.io). Trinotate collects annotation information into one database after running a number of annotation tools: homology search to SwissProt/Uniref90, protein domain identification using HMMER and PFAM, transmembrane domain prediction using tmHMM and comparison to functional categories such as Gene Ontology (GO) databases.

### Genome assembly

A sequential pipeline of Illumina and PacBio tools were used to generate the assembly. An initial assembly was generated by Allpaths-LG^20^ (release 44837), using reads from three Illumina libraries (fragment library and overlapping 3 kb and 6 kb libraries; described above). Allpaths-LG default parameters were used with ploidy set to two. The assembly comprised 1449 scaffolds with scaffold N50 of 1.0 Mb. PacBio reads were added to the assembly for scaffolding using AHA scaffolder v1.2.0, part of SMRT Analysis 2.0 suite (http://www.pacb.com/products-and-services/analytical-software/devnet/devnet-analysis-tools/). The AHA scaffolder uses BLASR^23^ to map long PacBio reads to the assembly without gap filling. Blasr parameters required a minimum seed match of 10 bp (-minMatch 10), minimum identity of 70% (-minPctIdentity 10), reporting 10 best matches (-bestn 10); spliiting of subreads was not permitted (-noSplitSubreads). Parameters for the AHA hybrid assembly were varied iteratively and are provided in Supplementary Text S1. Further alignment of long PacBio reads with the draft assembly to close or improve gaps was carried out in PBJelly (v12.9.14; http://sourceforge.net/projects/pb-jelly/)^24^. The protocol file used to run all PBJelly stages (setup, mapping, support, extraction, assembly, output) is provided in Supplementary Text S1.

*Rhazya* nuclei embedded in agarose and the sequence of these 232 scaffolds were provided to OpGen (Gaithersburg, MD) for optical mapping. OpGen generated 11 high density Argus^®^ MapCards using the BamHI restriction enzyme to obtain single molecule restriction maps for the *Rhazya* genome. Utilizing the initial *R. stricta* assembly and OpGen’s Genome Builder™ tool, OpGen compared the single molecule restriction data to the initial assembly to facilitate joining of scaffolds. If an overlap was detected when aligning the original *Rhazya* scaffolds to OpGen’s extension, OpGen assigned join scores. A join score of 9.0 or above indicated reliable joins. The 232 scaffolds were reduced to 113 scaffolds and the scaffold N50 increased from 1.45 Mb to 5.55 Mb. The whole genome map statistics provided by OpGen under a fee-for-service contract are available in Supplementary Table S3.

### Genome annotation

The MAKER2 annotation pipeline (v2.28b; https://www.biostars.org/p/45281/)^25^ was employed for genome annotation. RepeatMasker (v3-3-0; http://www.repeatmasker.org)^26^ was used to mask all repeat classes from Repbase (http://www.girinst.org/repbase/), and low complexity sequences. The RepeatRunner webserver (http://www.yandell-lab.org/software/repeatrunner.html)^27^ was used to identify transposable elements based on the RepeatRunner protein database.

The Trinity-assembled transcripts were used to query the UniProt database (http://www.uniprot.org). Identified ESTs and protein sequences were aligned to the *Rhazya* genome using BLASTN and BLASTX (blast 2.2.27+)^28^, respectively. Exonerate (v3-3-0; http://www.ebi.ac.uk/about/vertebrate-genomics/software/exonerate)^29^ was used to realign sequences identified by BLAST around splice sites to more accurately detect gene models. Est2genome (http://emboss.sourceforge.net/apps/release/6.6/emboss/apps/est2genome.html) was used for polishing the EST hits and protein2genome, part of Exonerate, was used for polishing the protein hits.

SNAP *ab initio* gene predictor (v.2006-07-28; http://korflab.ucdavis.edu/software.html)^30^ was used to predict gene models. SNAP is an easy to use, easy to train tool that is supported by the MAKER2 pipeline. The program was trained by running MAKER2 twice. In the first run, gene models were produced exclusively from EST and protein homology evidence. These gene models were used to train SNAP, and MAKER2 was rerun allowing for *ab initio* predictions using the newly trained gene predictor.

MAKER2 integrated all the EST alignments, protein alignments and *ab initio* predictions and updated information such as splice sites and UTR locations based on all the evidence to generate a filtered set of annotations. In addition to a final list of filtered and modified gene models, a list of rejected *ab initio* predictions were also generated for further detection of missing genes. All parameters used to run the various stages of MAKER2 annotation are provided in Supplementary Text S1

An additional 34,232 proteins were predicted by SNAP. These putative protein sequences were used to query a reference database consisting of annotated genes from *Arabidopsis thaliana*, *Solanum lycopersicum* (tomato), *Vitis vinifera* (grape), *Coffea canephora* (coffee), *Catharanthus roseus*, *Rauvolfia serpentina* and *Theobroma cacao* (>50% identity, evalue >0.000). The MAKER2 validated genes were also used to query the same reference database with the same identity and evalue requirements. If a SNAP predicted gene yielded a qualified hit to a gene in the reference database that had not been previously identified among the 17,041 validated genes, it was included in the final set of genes (4123 additional genes, Supplementary Table S5).

### Verification of genome assembly and annotation

The assembled *Rhazya* genome was checked for completeness by using CEGMA 2.5 (Core Eukaryotic Genes Mapping Approach)^8^. CEGMA consists of a core set of eukaryotic genes that are highly conserved across eukaryotic taxa. The CEGMA genes were mapped to the *Rhazya* genome and a report indicating the completeness of the genome was generated.

### Functional categorization of genome

All *Rhazya* transcripts were functionally annotated and were assigned gene names, functions, generic Gene Ontology (GO) terms and Enzyme codes (EC) by blasting to the UniProt protein database using BLAST settings (BLASTX, report 1 hit, maximum e-value 1E^−6^, percent identity greater than 40).

### Metabolic pathway prediction

A metabolic pathway database was created for *Rhazya* using the PathoLogic component of Pathway Tools (v18.0; http://brg.ai.sri.com/ptools/)^31^. This program inferred metabolic pathways by comparing the annotated *Rhazya* genes to the MetaCyc reference pathway database (v.2.0; http://www.metacyc.org)^32^. MetaCyc contains experimentally elucidated, non-redundant metabolic pathways for multiple organisms and serves as a high quality reference for predicting pathways specific to an organism of interest. It also contains information about the enzymes, chemical compounds, reactions and genes related to these metabolic pathways.

The Pathologic Pathway Predictor algorithm consists of two phases. In the reactome inference phase, the predictor evaluates every annotated gene product in the genome and infers the reactions catalyzed by that gene product. The reactions catalyzed by a gene product are inferred from three information fields present in the input: the EC number, gene product name (enzyme name), and Gene Ontology (GO) terms. In the pathway inference phase, the tool infers the metabolic pathways present in the organism based on the set of catalyzed reactions. A pathway is inferred if it is not missing more than one reaction, or if a reaction that is unique to that pathway is present in the organism of interest. Nucleotide and amino acid sequence information is not considered by Pathologic in making these inferences. RhaCyc is available as a community resource at http://rhacyc.icmb.utexas.edu:1555/.

### Structural evolution analysis

We used SynMap (with default parameters) in the CoGe platform (https://genomevolution.org/CoGe/; https://www.genomevolution.org/CoGe/SynMap.pl), to find syntenic blocks between *Rhazya* and *Coffea* (Rubiaceae, Gentianales). A whole genome triplication, discovered by Jaillon *et al*.^10^ while sequencing the *Vitis vinifera* genome, is broadly accepted to have occurred at the root of the core eudicots. This polyploidization, known as γ(gamma), produced a twenty-one chromosome genome from the seven chromosome ancestor. It is not too difficult to reconstruct the general structure of the immediate post-γ chromosomes since they differ from the nineteen-chromosome *Vitis* genome in only five or six large rearrangements. This baseline genome is the ultimate resource for deducing gene-order change in the core eudicots^33^.

Within each pair of genomes among *Rhazya*, *Vitis* and *Coffea*, SynMap synteny blocks together with overall gene similarity and Ks scores were used to establish thresholds for distinguishing duplicate gene pairs due to speciation (orthologs) which were retained for our analysis, from the older, weaker and sparser gene pairs due to the gamma triplication (“out-paralogs”), which were discarded. The coloring of the blocks was according to the 21 ancestral core eudicot chromosomes, as published by Zheng *et al*.^33^.

The circular display of homologies arrayed on the *Rhazya* scaffolds and *Coffea/Vitis* chromosomes were drawn with CIRCOS (http://circos.ca/)^34^.

The Bergeron *et al*.^35^ algorithm for double-cut-and-join (DCJ) was used to finding the shortest path between two genomes. Xu’s 2008 median solver for DCJ^36^ was employed to infer the Gentianales ancestor.

### Gene family analysis

The subject database comprised protein sequences for six taxa. The species selected for inclusion in the MIA gene family analyses were required to meet certain criteria. The Apocynaceae taxa examined were required to produce MIAs, which restricted the sampling to the subfamily Rauvolfioideae^37^. The other three taxa included have complete genomes, which provided important information about isoforms so that only one isoform would be included in the gene tree analyses. In particular, coffee was selected because it is in the Gentianales, produces MIAs and its complete genome is available^9^. Tomato was included as an asterid outgroup to the Gentianales as its transcriptome and complete nuclear genome were available^38^. Likewise, *Theobroma cacao*, which produces non-MIA alkaloids, was included as a rosid outgroup to the asterid clade and had the required data available^39^. Protein sequences were downloaded for five species and combined with *Rhazya* sequences. Sequence data was accessed from the following sites: *Theobroma cacao* (rosid; http://www.cacaogenomedb.org/Tcacao_genome_v1.1), *Solanum lycopersicum* (non-Gentianales asterid; https://solgenomics.net/gb2/gbrowse/ITAG2.3_genomic/), *Coffea canephora* (Rubiaceae, Gentianales; http://coffee-genome.org), *Catharanthus roseus* and *Rauvolfia serpentina* (Apocynaceae, Gentianales; http://medicinalplantgenomics.msu.edu). Sequences for each of 21 characterized proteins involved in the MIA pathways of *Catharanthus roseus* (L.) and/or *Rauvolfia serpentina* (Supplementary Table S7) were used to query the database using BLAST (e-value 1e^−3^) and sequences with identities ≥ 35% were extracted. A single isoform was retained for each unique genetic locus and the sequences were aligned with the GenBank homolog (query) using MUSCLE^40^ as implemented in Geneious (www.biomatters.com). Sequences covering < 50% of the length of the GenBank homolog were removed. If the database sequence extended beyond the GenBank homolog sequence, the extra sequence was trimmed. Following removal of sequences, alignments were rerun with MUSCLE for maximum likelihood (ML) analysis with PhyML^41^ in Geneious. Muscle settings included eight iterations with sequences grouped by similarity using the kmer 6_6 for the first iteration and pctid_kimura distance for all subsequent iterations and the neighbor joining clustering method. ML analyses used the Le Gascuel substitution model and Subtree Pruning and Regrafting topology search options.

A species tree was drawn in FigTree (see top Fig. 2) and follows APG (http://www.mobot.org/MOBOT/Research/APweb/) relationships. For reconciliation of gene trees with the species tree eight datasets were selected representing early and late stage enzymes in the MIA pathways of *C. roseus* and *R. serpentina*. For these eight (bold in Supplementary Table 7), an iterative series of alignment and ML tree generation was used to define a well-supported clade that included the GenBank homolog. For each gene family, sequences for the clade of interest were extracted and aligned with MUSCLE and a preliminary unrooted binary phylogenetic tree was computed using PhyML version 20131022 (GTR + Γ(4) model). PhyML parameters were set to optimize branch length, tree topology and substitution rate. A hundred non-parametric bootstraps were performed to assess clade support in the resulting tree.

The initial trees were refined, rooted and internal nodes labeled as duplication or speciation nodes for reconciliation with the species tree. Weakly supported branches, i.e. branches with bootstrap values under a given threshold, were removed leading to an unrooted non-binary tree. Different thresholds for the bootstrap value (20, 30, 40, 50, 60, 70, 80 and 90) were tested. For each obtained tree, all nodes were successively considered as potential roots. Then for each rooted non-binary tree, all binary refinements minimizing the reconciliation cost, i.e. the inferred number of duplication and loss operations according to the species tree, were computed using ProfileNJ^42^. Each tree output by ProfileNJ is a rooted binary tree with each internal node labeled as a duplication (red square) or speciation (green circle), and each lost gene reported on the tree by grafting a new branch (gray dashed branches) and leaf labeled with abbreviated names of the genomes affected by the loss. This was the set of trees used as replicates for node bootstrapping.

Per-site likelihood for all trees was computed with RaxML^43^ version 8.1.3, and SH and AU tests were performed with CONSEL^44^. Then, manual filtering was done to retain only the best trees according to both the likelihood score and the reconciliation cost (Supplementary Table S8). A consensus tree was computed from all retained best trees using *consense,* part of the PHYLIP package (http://evolution.genetics.washington.edu/phylip.html), with extended majority rule for identifying most frequent subtrees. Among the best trees, the one sharing the closest topology to the consensus tree was chosen. As branch length and bootstrap values were lost for branches contracted with ProfileNJ, they were recomputed for each tree. Finally, a node support was computed as the frequency of replicates having that node, i.e. with the same incoming edge and the same inferred event (duplication or speciation).

### Data Availability

This Whole Genome Shotgun project has been deposited at DDBJ/ENA/GenBank under the accession MEJB00000000. The version described in this paper is version MEJB01000000. All phylogenetic alignments are available upon request to TAR or RJK.

## Additional Information

**How to cite this article**: Sabir, J. S.M. *et al*. The nuclear genome of *Rhazya stricta* and the evolution of alkaloid diversity in a medically relevant clade of Apocynaceae. *Sci. Rep.* **6**, 33782; doi: 10.1038/srep33782 (2016).

## Supplementary Material

Supplementary Information
