# Supplementary material for: The nuclear genome of Rhazya stricta and the evolution of alkaloid diversity in a medically relevant clade of Apocynaceae
Source: Sci Rep. 2016 Sep 22;6:33782. doi: 10.1038/srep33782 (PMC5031960; doi:10.1038/srep33782)
Supplement: Supplementary Information [file srep33782-s1.pdf]

The nuclear genome of *Rhazya stricta* and the evolution of alkaloid diversity in a medically relevant clade of Apocynaceae

Jamal S.M. Sabir[1]†, Robert K. Jansen[1,2]†, Dhivya Arasappan[2], Virginie Calderon[3], Emmanuel Noutahi[3], Chunfang Zheng[4], Seongjun Park[2], Meshaal J. Sabir[1], Mohammed N. Baeshen[9], Nahid H Hajrah[1], Mohammad A. Khiyami[5], Nabih A. Baeshen[6], Abdullah Y. Obaid[7], Abdulrahman L. Al-Malki[8], David Sankoff[4], Nadia El-Mabrouk[3] and Tracey A. Ruhlman[2*]

Supplementary Information_SREP-16-05184

Supplementary Table S1: Data generated for genome assembly

Supplementary Table S2: Improvement of assembly with PacBio and optical mapping.

Supplementary Table S3: OpGen whole genome map statistics.

Supplementary Table S4: Comparison of assembly statistics for four plant nuclear genomes.

Supplementary Table S5: Coding sequence annotation for Rhazya nuclear genome.

Supplementary Table S6: Repeated sequences of the *Rhazya* nuclear genome.

Supplementary Table S7: Query sequences used in homolog search.

Supplementary Table S8: Results from reconciliation with different bootstraps cut-off, all possible roots. Results obtained after manual filtering considering both likelihood and reconciliation cost.

Supplementary Table S9: Monoterpenoid indole alkaloids (MIA) isolated from *Rhazya stricta*. Extracts from young *R. stricta* leaves were evaluated for MIA content by mass spectrometry.

Supplementary Text S1. Extended methods for genome assembly and annotation.

Supplementary Figures and Legends S1-S3.

Supplementary Table S1: Data generated for genome assembly

Technology	Library type	Insert size (bp)	Read length (bp)	Number of reads
Illumina	Fragment	160-220	100	291,361,582
Illumina	Mate-pair	3,000	100	78,042,292
Illumina	Mate-pair	6,000	100	26,102,232
PacBio	Continuous long read	10,000	50-22,603	579,535
Optical mapping (OpGen)			Scaffold joins	

bp=base pairs

Supplementary Table S2: Improvement of assembly with PacBio and optical mapping.

Refinement steps	Nnumber of scaffolds	N50	Max lenghth	Scaffolds >200KB	
				count	% of assembly (274Mb)
Assembled Illumina reads	1449	1,061,426	4,551,498	n.a.	n.a.
Scaffolding with PacBio (AHA), Close gaps with PacBio (PBJelly)	1099	1,445,001	6,923,550	232	93
Merge large scaffolds with optical mapping	980	5,554,218	16,403,306	113	93

Mb= megabases

Supplementary Table S3: OpGen whole genome map statistics. Data provided by OpGen.

Summary of SMRM data	Maps used in analysis
Total size (Mb)	104,229.97
Number of molecules	291,892
Average size of molecules (kb)	357.08
Minimum molecule size (kb)	250
Average size of fragments (kb)	14.94

Mb= megabases, kb= kilobases

Supplementary Table S4: Comparison of assembly statistics for four plant nuclear genomes.

Taxon	Genome size estimate (Mb)	No. of scaffolds	Assembly size	Scaffold N50 (Mb)
**Coffea**	710	13,345	568.6	1.26
**Cacao**	430	4,792	326.9	0.47
**Amborella**	870	5,745	706.0	4.9
**Rhazya**	200	980	274.0	5.5

Mb=megabases

Supplementary Table S5: Coding sequence annotation for *Rhazya* nuclear genome.

**Number of genes in filtered list**	**17,041**	
**Additional SNAP genes added to the filtered list**	**4,123**	
	**Total**	**21,164**
**Number of exons**		107,181
**Number of CDS**		104,172
**Average gene size (bp)**		4,452
**Average number of exons per gene**		6.29

bp=base pairs

Supplementary Table S6: Repeated sequence estimate for the *Rhazya* nuclear genome.

Class	Order	Superfamily	Number of elements	Sequence Occupied (bp)	Proportion of genome
Retro transposons	LTR	Copia	19,698	15,260,318	5.6%
		Gypsy	29,986	18,810,088	6.9%
		DIRS	120	7,332	>0.01%
		ERV	864	56,368	>0.01%
		Others	8,690	5,072,218	1.8%
	LINE		4,060	682,122	0.2%
	SINE		230	17,004	>0.01%
DNA transposons	TIR	Tc1-Mariner	1,126	200,788	0.1%
		hAT	2,492	877,146	0.3%
		Harbinger	676	172,602	0.1%
		Others	7,314	1524,456	0.6%
Low complexity sequence			227,112	10,959,376	4.0%
	Total			55498610	20.2%*

*This list of repeated elements is not exhaustive and the actual value for repeat content is likely higher. bp=base pairs, LTR=long terminal repeats, LINE= Long interspersed elements, SINE=Short interspersed elements, TIR= Terminal Inverted Repeats

Supplementary Table S7. Query sequences used in homolog search.

SYMBOL	FULL NAME	*IntEnz	SOURCE	ACCESSION	FIG#
**AAE**	Acetylajmaline esterase	EC 3.1.1.80	RSA	Q3MKY2	ED_2c
**SGD**	Strictosidine beta-glucosidase	EC 3.2.1.105	CRA<br>RSA	AAF28800<br>Q8GU20	ED_2n
**PNAE**	Polyneuridine aldehyde esterase	EC 3.1.1.78	RSA	Q9SE93	ED_2j
**STR**	Strictosidine synthase	EC 4.3.3.2	CRA<br>RSA	P18417<br>P68175	ED_2p
**T16H**	Tabersonine 16-hydroxylase_CYP71D12	EC 1.14.13.73	CRA	P98183	ED_2q
**THAS**	tetrahydroalstonine synthase	EC 1.1.1	CRA	AKF02528	ED_2t
**VS**	Vinorine synthase<br>70%VS homolog_cathcyc	EC 2.3.1.160	RSA<br>CRA	Q70PR7<br>GK13-15088	ED_2u
**D4H**	deacetoxyvindoline 4-hydroxylase	EC 1.14.11.20	CRA	O04847	ED_2e
7DLS	7-deoxyloganic acid hydroxylase_CYP72A224	EC 1.14.13	CRA	AHK60834	ED_2a
16OMT	Tabersonine 16-hydroxylase_CYP71D12	EC 1.14.13.73	CRA	P98183	ED_2b
DAT	Deacetylvindoline O-acetyltransferase	EC 2.3.1.107	CRA	Q9ZTK5	ED_2d
G10H	Geraniol 8-hydroxylase_CYP76B6†	EC 1.14.13.152	CRA	Q8VWZ7	ED_2f
LAMT	Loganate O-methyltransferase	EC 2.1.1.50	CRA	AGX93063	ED_2g
MAT	Minovincinine 19-hydroxy-O-acetyltransferase	EC 2.3.1	CRA	AAO13736	ED_2h
NMT	16-hydroxy-2,3-dihydro-3-hydroxytabersonine n-methyltransferase	EC 2.1.1.99	CRA	AHH33092	ED_2i
PR	Perakine reductase	EC 1.1.1.317	RSA	AAX11684	ED_2k
PRX1	Peroxidase 1	EC 1.11.1	CRA	CAJ84723	ED_2l
RO	Reticuline oxidase	EC 1.21.3.3	CRA	AKF02526	ED_2v
TDC	Tryptophan decarboxylase	EC 4.1.1.27	CRA	P17770	ED_2s
SLS	Secologanin synthase_CYP72A1	EC 1.3.3.9	CRA	Q05047	ED_2o
T19H	Tabersonine/lochnericine 19-hydroxylase _CYP71BJ1	EC 1.14.13	CRA	ADZ48681	ED_2r

*http://www.ebi.ac.uk/intenz/query?cmd=SearchEC&ec=3.2.1.105.
†Gene CYP76B6 encodes a protein with alternate names (Geraniol 8-hydroxylase and 10-hydroxylase).
CRA=*Catharanthus roseus*, RSA=*Rauvolfia serpentina*

Supplementary Table S8: Results from reconciliation with different bootstrap cut-offs and all possible roots. Results obtained after manual filtering considering both likelihood and reconciliation cost.

Query	All possible roots		Number of solutions	Lowest reconciliation cost	Reference figure
	Dup	loss			
**AAE**	26	7-11	11	33	ED_3a
**D4E**	20-21	14-17	10	34	ED_3b
**PNAE**	16-18	3-11	12	23	ED_3c
**SGD**	27	25-29	12	52	ED_3d
**STR**	28-29	20-25	13	48	ED_3e
**T16H**	26	14-21	9	40	ED_3f
**THAS**	11	13-20	13	24	FIG 3
**VS**	11	6-13	11	19	ED_3g

Dup=duplications, query abbreviations are expanded in Supplementary Table S7.

Supplementary Table S9: Monoterpenoid indole alkaloids isolated from *Rhazya stricta* leaves

#ID	Structure	Name	FW
1	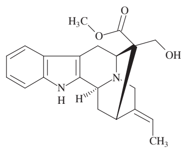	Akuammidine	352.4269
2	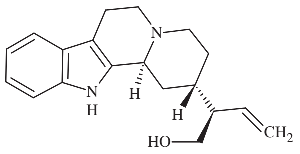	Antirhine	296.4067
3	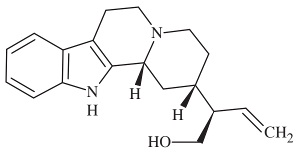	3-epi-Antirhine	296.4067
4	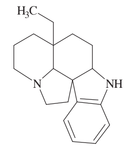	Aspidosespermidine	282.4231
5	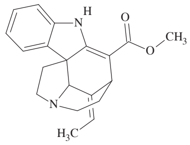	Condylocarpine	322.4009
6	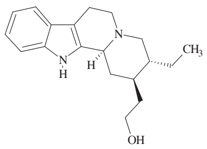	dihydrocorynantheol	298.4225
7	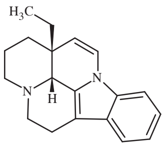	eburnamenine	278.3914
8	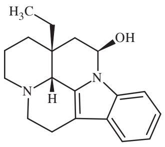	eburnamine	296.4067
9	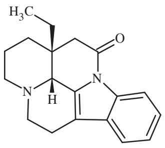	Eburnamonine	294.3908
10	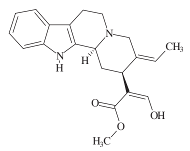	geissoschizine	352.4269
11	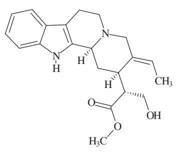	Isositsirikine	354.4427

#ID	Structure	Name	FW
12	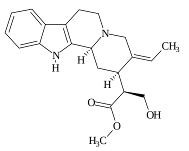	16-epi-Z-isositsirikine	354.4427
13	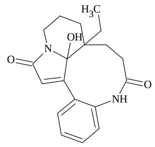	leuconolam	326.3896
14	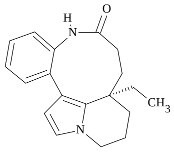	rhazinilam	294.3908
15	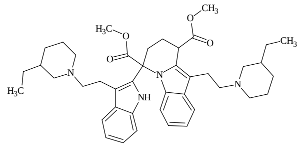	tetrahydrosecamine	680.9184
16	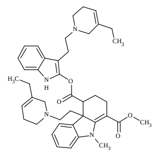	presecamine	676.8867
17	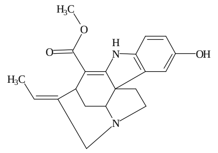	sewarine	338.4003
18	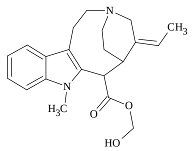	Stemmadenine	354.4427
19	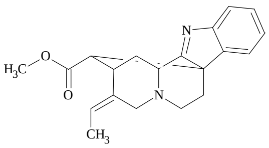	strictamine	322.4009
20	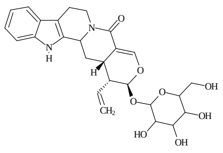	strictosamide	498.5250
21	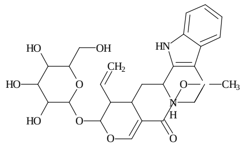	strictosidine	530.5669
22	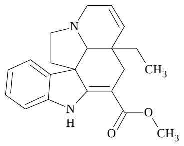	tabersonine	336.4275

#ID	Structure	Name	FW
23	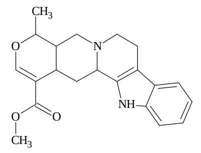	Tetrahydroalstonine	352.4269
24	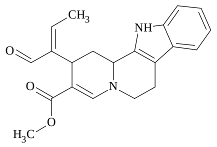	vallesiachotamine	350.4110
25	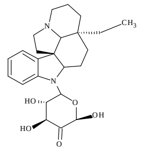	Aspidospermiose	428.5213
26	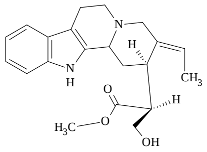	Bhimberine	354.4427
27	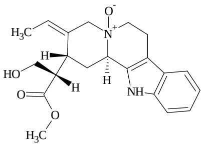	Bhimbrine N-Oxide	370.4421
28	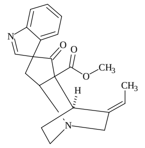	Rhazimine	350.4110
29	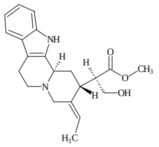	Rhazimanine	354.4427
30	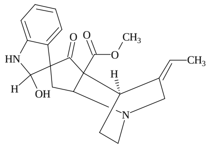	Rhazicine	368.4263
31	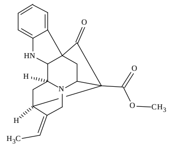	Leepacine	350.4110
32	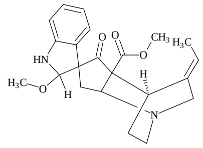	2-Methoxy-1,2-Dihydrorhazimine	382.4528
33	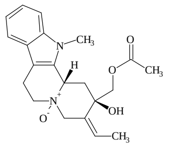	HR-1	370.4421

#ID	Structure	Name	FW
34	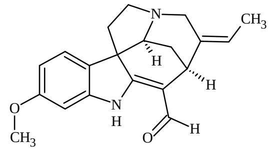	Vincanicine	322.4009
35	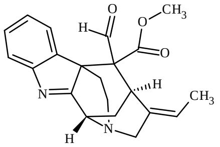	Rhazinaline	350.4110
36	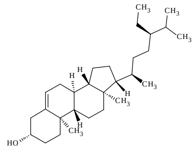	beta-Sitosterol	414.7067
37	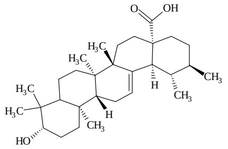	Ursolic acid	456.7003
38	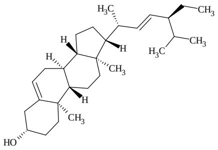	Stigmasterol	412.6908
39	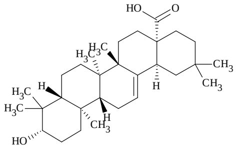	Oleanolic acid	456.7003
40	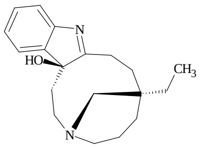	Rhazidigenine (Rhazidine,Stric tanol)	298.4225
41	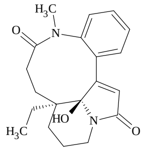	N-methylleuconolam	340.4162
42	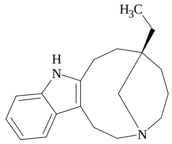	(+)-Quebrachamine	282.4231
43	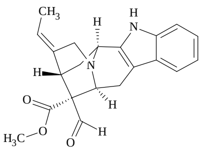	Polyneuridine	350.4110
44	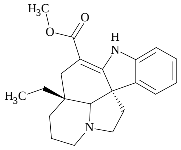	(+)-Vincadiformine	338.4433

#ID	Structure	Name	FW
45	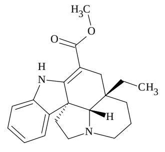	(-)-Vincadifformine	338.4433
46	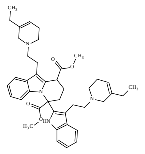	Secamine	676.8867
47	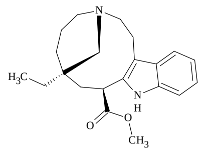	Vincadine	340.4592
48	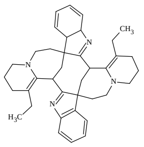	bis-strictidine	558.7986
49	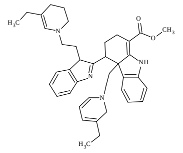	3,14-dehydrorhazigine	616.8347
50	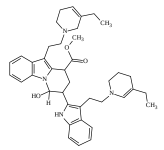	16-hydrorhazisidine	634.8500
51	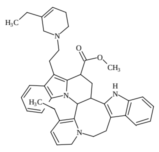	rhazisidine	614.8188
52	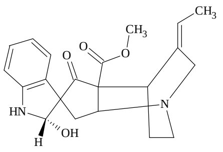	Isorhazicine	368.4263
53	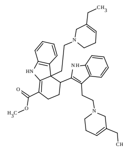	rhazigine	618.8506
54	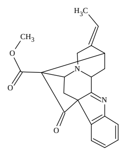	strictisidine	348.3951
55	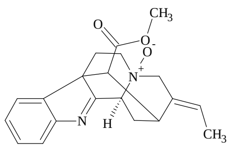	strictamine-N-oxide	338.4003

#ID	Structure	Name	FW
56	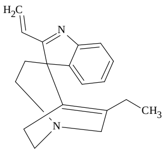	strictigine	278.3914
57	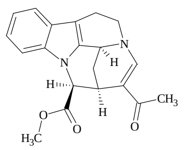	strictine	336.3844
58	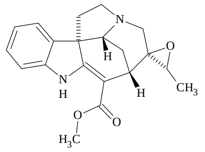	stricticine	338.4003
59	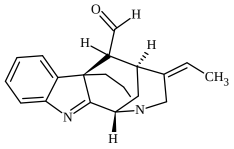	strictalamine	292.3749
60	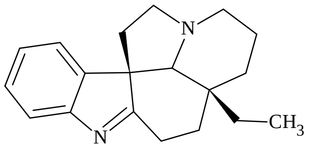	1,2-Dehydroaspidospermidine(eburenine)	280.4073
61	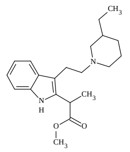	Tetrahydrosecodine	342.4751
62	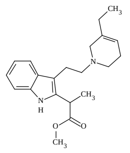	Dihydrosecodine	340.4592
63	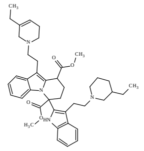	Dihydrosecamine	678.9026
64	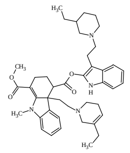	Dihydropresecamine	678.9026
65	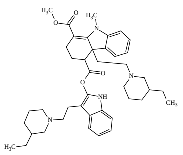	tetrahydropresecamine	680.9184
66	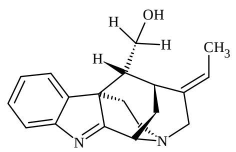	rhazinol	294.3908

#ID	Structure	Name	FW
67	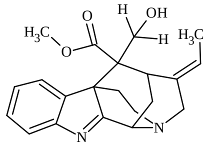	Rhazimol	352.4269
68	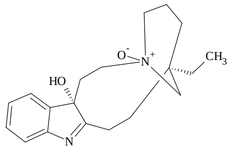	Rhazidigenine-N-oxide	314.4219
69	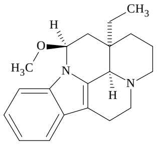	(-)16R,21R-omethyleburnamine	310.4332
70	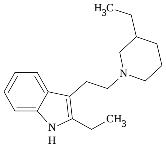	Decarbomethoxy-15,20,16,17-te trahydrosecodine	284.4390
71	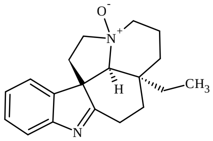	1-2-dehydroasidospermidine-N -oxide	296.4067
72	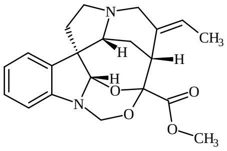	rhazizine	368.4263
74	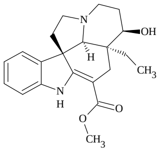	15-hydroxyvincadifformine	354.4427
76	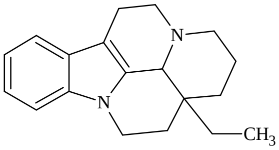	Dihydroeburnamenine	280.4073
77	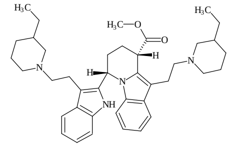	16s,16'-decarboxytetra-hydros ecamine	622.8824
78	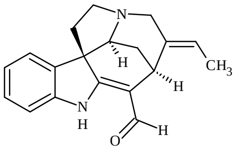	Nor-C-fluorocurarine	292.3749
79	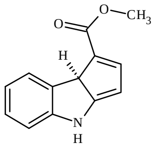	strictibine	213.2319

Supplementary Text S1

Iteratively varied parameters for AHA assembly

For running the iterative hybrid assembly, these parameters were varied iteratively.
Comma separates out the values per iteration.
- Minimum alignment score (aka Z-score) 6,6,6,6
- Minimum number of reads needed to link two contigs. 3,3,2,2
- Minimum subread length to participate in alignment. 75,75,75,75

Protocol file for PBJelly

<jellyProtocol>

<reference>/scratch/01184/daras/jansen/RHA/pbjelly/RHA_small_75per_overlapping/data/reference/final.assembly.fasta</reference>

<outputDir>/scratch/01184/daras/jansen/RHA/pbjelly/RHA_small_75per_overlapping/</outputDir>
<blasr>-minMatch 8 -minPctIdentity 70 -bestn 8 -nCandidates 30 -maxScore -500 -nproc 8 -noSplitSubreads</blasr>
<input baseDir="/scratch/01184/daras/jansen/RHA/pbjelly/RHA_small_75per_overlapping/data/reads/">
<job>Jansen_Pacbio.cat.fasta</job>
</input>
</jellyProtocol>

Run parameters for MAKER2

Maker_bopts.ctl

#-----BLAST and Exonerate Statistics Thresholds
blast_type=ncbi+ #set to 'ncbi+', 'ncbi' or 'wublast'

pcov_blastn=0.8 #Blastn Percent Coverage Threhold EST-Genome Alignments
pid_blastn=0.85 #Blastn Percent Identity Threshold EST-Genome Aligments
eval_blastn=1e-10 #Blastn eval cutoff
bit_blastn=40 #Blastn bit cutoff
depth_blastn=0 #Blastn depth cutoff (0 to disable cutoff)

pcov_blastx=0.5 #Blastx Percent Coverage Threhold Protein-Genome Alignments
pid_blastx=0.4 #Blastx Percent Identity Threshold Protein-Genome Aligments

eval_blastx=1e-06 #Blastx eval cutoff
bit_blastx=30 #Blastx bit cutoff
depth_blastx=0 #Blastx depth cutoff (0 to disable cutoff)

pcov_tblastx=0.8 #tBlastx Percent Coverage Threhold alt-EST-Genome Alignments
pid_tblastx=0.85 #tBlastx Percent Identity Threshold alt-EST-Genome Aligments
eval_tblastx=1e-10 #tBlastx eval cutoff
bit_tblastx=40 #tBlastx bit cutoff
depth_tblastx=0 #tBlastx depth cutoff (0 to disable cutoff)

pcov_rm_blastx=0.5 #Blastx Percent Coverage Threhold For Transposable Element Masking
pid_rm_blastx=0.4 #Blastx Percent Identity Threshold For Transposbale Element Masking
eval_rm_blastx=1e-06 #Blastx eval cutoff for transposable element masking
bit_rm_blastx=30 #Blastx bit cutoff for transposable element masking

ep_score_limit=20 #Exonerate protein percent of maximal score threshold
en_score_limit=20 #Exonerate nucleotide percent of maximal score threshold

Maker_exe.ctl

#-----Location of Executables Used by MAKER/EVALUATOR
makeblastdb=/scratch/projects/tacc/bio/maker/2.28b/blast/bin/makeblastdb #location of NCBI+ makeblastdb executable
blastn=/scratch/projects/tacc/bio/maker/2.28b/blast/bin/blastn #location of NCBI+ blastn executable
blastx=/scratch/projects/tacc/bio/maker/2.28b/blast/bin/blastx #location of NCBI+ blastx executable
tblastx=/scratch/projects/tacc/bio/maker/2.28b/blast/bin/tblastx #location of NCBI+ tblastx executable
formatdb= #location of NCBI formatdb executable
blastall= #location of NCBI blastall executable
xdformat= #location of WUBLAST xdformat executable
blasta= #location of WUBLAST blasta executable
RepeatMasker=/scratch/projects/tacc/bio/maker/2.28b/RepeatMasker/RepeatMasker #location of RepeatMasker executable
exonerate=/scratch/projects/tacc/bio/maker/2.28b/exonerate/bin/exonerate #location of exonerate executable

#-----Ab-initio Gene Prediction Algorithms
snap=/scratch/projects/tacc/bio/maker/2.28b/snap/snap #location of snap executable
gmhmme3= #location of eukaryotic genemark executable
gmhmmp= #location of prokaryotic genemark executable

augustus=/scratch/projects/tacc/bio/maker/2.28b/augustus/bin/augustus #location of augustus executable
fgenesh= #location of fgenesh executable

#-----Other Algorithms
probuild= #location of probuild executable (required for genemark)

Maker_opts.ctl

#-----Genome (these are always required)
genome=opgenResult+scaffoldsLengthsLess200.fasta #genome sequence (fasta file or fasta embeded in GFF3 file)
organism_type=eukaryotic #eukaryotic or prokaryotic. Default is eukaryotic

#-----Re-annotation Using MAKER Derived GFF3
maker_gff= #MAKER derived GFF3 file
est_pass=0 #use ESTs in maker_gff: 1 = yes, 0 = no
altest_pass=0 #use alternate organism ESTs in maker_gff: 1 = yes, 0 = no
protein_pass=0 #use protein alignments in maker_gff: 1 = yes, 0 = no
rm_pass=0 #use repeats in maker_gff: 1 = yes, 0 = no
model_pass=0 #use gene models in maker_gff: 1 = yes, 0 = no
pred_pass=0 #use ab-initio predictions in maker_gff: 1 = yes, 0 = no
other_pass=0 #passthrough anyything else in maker_gff: 1 = yes, 0 = no

#-----EST Evidence (for best results provide a file for at least one)
est=trinityOut.Trinity.fasta #set of ESTs or assembled mRNA-seq in fasta format
altest= #EST/cDNA sequence file in fasta format from an alternate organism
est_gff= #aligned ESTs or mRNA-seq from an external GFF3 file
altest_gff= #aligned ESTs from a closly relate species in GFF3 format

#-----Protein Homology Evidence (for best results provide a file for at least one)
protein=uniprot_sprot.fasta #protein sequence file in fasta format (i.e. from mutiple oransisms)
protein_gff= #aligned protein homology evidence from an external GFF3 file

#-----Repeat Masking (leave values blank to skip repeat masking)
model_org=all #select a model organism for RepBase masking in RepeatMasker
rmlib= #provide an organism specific repeat library in fasta format for RepeatMasker
repeat_protein=/opt/apps/maker/2.30/data/te_proteins.fasta #provide a fasta file of transposable element proteins for RepeatRunner
rm_gff= #pre-identified repeat elements from an external GFF3 file
prok_rm=0 #forces MAKER to repeatmask prokaryotes (no reason to change this), 1 = yes, 0 = no

softmask=1 #use soft-masking rather than hard-masking in BLAST (i.e. seg and dust filtering)

#-----Gene Prediction
snaphmm=RHA.hmm #SNAP HMM file
gmhmm= #GeneMark HMM file
augustus_species= #Augustus gene prediction species model
fgenesh_par_file= #FGENESH parameter file
pred_gff= #ab-initio predictions from an external GFF3 file
model_gff= #annotated gene models from an external GFF3 file (annotation pass-through)
est2genome=0 #infer gene predictions directly from ESTs, 1 = yes, 0 = no
protein2genome=1 #infer predictions from protein homology, 1 = yes, 0 = no
unmask=0 #also run ab-initio prediction programs on unmasked sequence, 1 = yes, 0 = no

#-----Other Annotation Feature Types (features MAKER doesn't recognize)
other_gff= #extra features to pass-through to final MAKER generated GFF3 file

#-----External Application Behavior Options
alt_peptide=C #amino acid used to replace non-standard amino acids in BLAST databases
cpus=24 #max number of cpus to use in BLAST and RepeatMasker (not for MPI, leave 1 when using MPI)

#-----MAKER Behavior Options
max_dna_len=100000 #length for dividing up contigs into chunks (increases/decreases memory usage)
min_contig=1 #skip genome contigs below this length (under 10kb are often useless)

pred_flank=200 #flank for extending evidence clusters sent to gene predictors
pred_stats=0 #report AED and QI statistics for all predictions as well as models
AED_threshold=1 #Maximum Annotation Edit Distance allowed (bound by 0 and 1)
min_protein=0 #require at least this many amino acids in predicted proteins
alt_splice=0 #Take extra steps to try and find alternative splicing, 1 = yes, 0 = no
always_complete=0 #extra steps to force start and stop codons, 1 = yes, 0 = no
map_forward=0 #map names and attributes forward from old GFF3 genes, 1 = yes, 0 = no
keep_preds=0 #Concordance threshold to add unsupported gene prediction (bound by 0 and 1)

split_hit=10000 #length for the splitting of hits (expected max intron size for evidence alignments)
single_exon=0 #consider single exon EST evidence when generating annotations, 1 = yes, 0 = no

single_length=250 #min length required for single exon ESTs if 'single_exon is enabled'
correct_est_fusion=0 #limits use of ESTs in annotation to avoid fusion genes

tries=2 #number of times to try a contig if there is a failure for some reason
clean_try=0 #remove all data from previous run before retrying, 1 = yes, 0 = no
clean_up=0 #removes theVoid directory with individual analysis files, 1 = yes, 0 = no
TMP= #specify a directory other than the system default temporary directory for temporary files

Supplementary Figure S1: Divergent structural evolution in *Rhazya stricta*. Genome level rearrangements leading from the ancestral core eudicot, represented by *Vitis* (upper right hand corner) to *Rhazya* and *Coffea*. Each linear arrangement represents successive steps from the eudicot ancestor toward the Gentianales ancestor. An arrow leads from the last of these stages to the relevant portion of the Gentianales ancestor. From this ancestor, two arrows indicate the divergence of *Coffea* and *Rhazya*, and successive rearrangements within each lineage are again indicated by linear arrangements of colored blocks. Inferred rearrangements between lineages are given at the arrows. Extant genomes carry the species name. The circular diagrams at the lower left and right represent the current orthologies between *Rhazya* and *Coffea* and *Vitis*, respectively. Syntenic blocks are colored according to the 21 ancestral core eudicot chromosomes[33] and do not represent biologically significant units. a) inferred orthologies for *Rhazya* superscaffolds 7, 9 and 10; b) superscaffolds 12, 13 and 28.

Supplementary Figure S2: Maximum likelihood phylogenies for 21 monoterpenoid indole alkaloid (MIA) sequences. Sequences of experimentally verified genes from GenBank for *Catharanthus* and *Rauvolfia* are indicated with a red arrow and accession numbers for these sequences are in Supplementary Table S7. Cra, *C. roseus*; Cc, *Coffea canephora*; rha, *Rhazya stricta*; rsa, *R. serpentina*; tom, *Solanum lycopersicum*; thec, *Theobroma cacao*. Full names for protein abbreviations in upper left corner of each tree are in Supplementary Table S7.

Supplementary Figure S3: Duplication and loss of selected monoterpenoid indole alkaloid (MIA) enzymes across six angiosperms. Gene phylogenies were reconciled

with the species phylogeny to infer duplication and loss history for each enzyme family. Internal and terminal branches in black reflect the amount of change along branch; dashed gray branch extensions are present for branches whose lengths were too short to enable uniform representation with branch support and length information. cra, *Catharanthus roseus*; cof, *Coffea canephora*; rha, *Rhazya stricta*; rsa, *Rauvolfia serpentina*; tom, *Solanum lycopersicum*; the, *Theobroma cacao*. Species indicated in blue font were downloaded from GenBank and those in red are from *Rhazya stricta*. Red square = gene duplication; green circle = speciation event; wide gray dashed line ending with a circle = gene loss. Full names for protein abbreviations in upper left corner of each tree are in Supplementary Table S7. Values above branches indicate bootstrap support for the clade, numbers below nodes give bootstrap support for the event (duplication or speciation) at the node. Bootstrap values less than 50 are not shown; inferred loss events are indicated with an asterisk and do not have associated bootstrap values. Scale bar represents number of amino acid substitutions per site. The maximum likelihood (ML) tree shown is the tree with the best ML score.

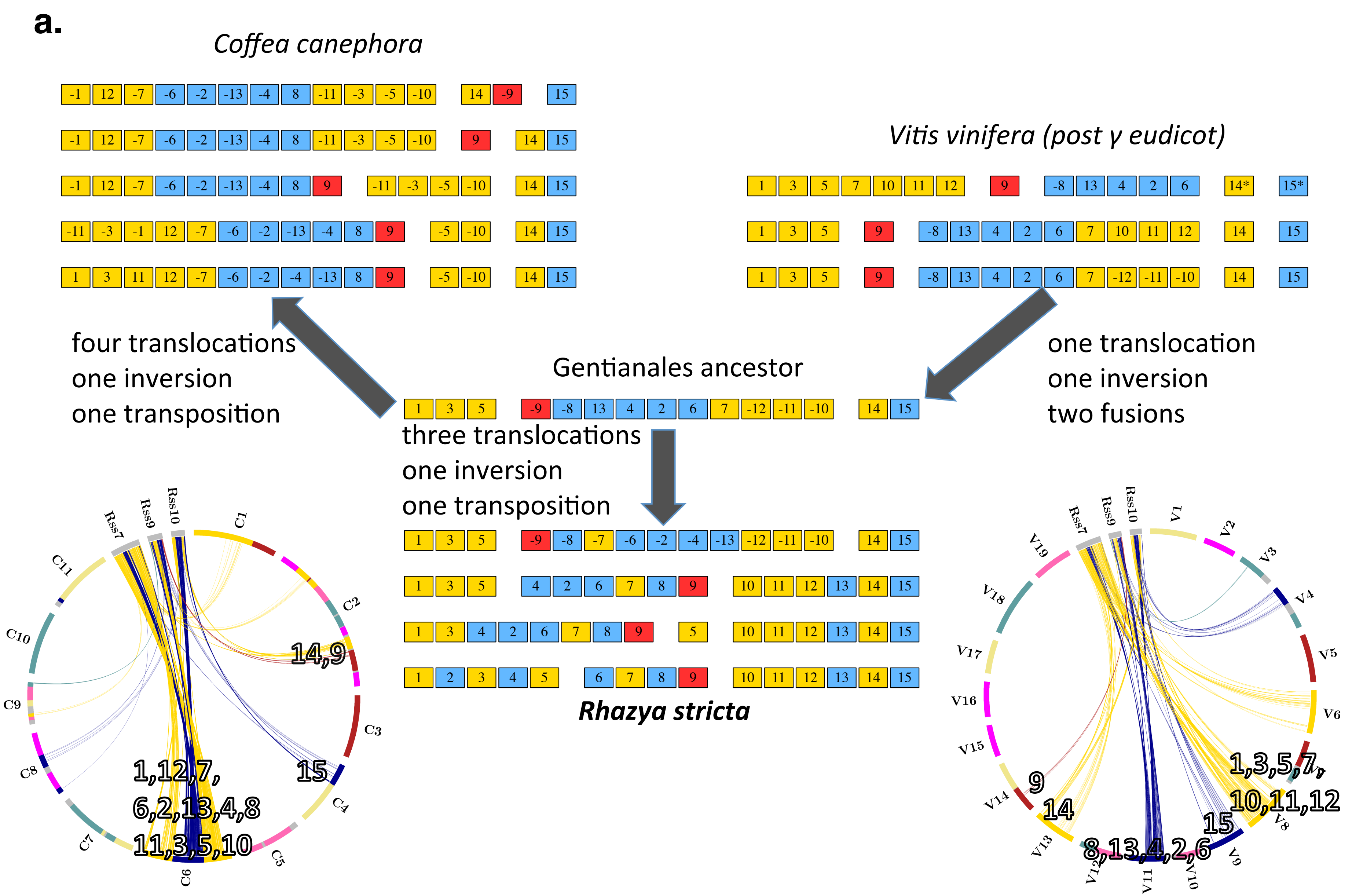

a.
Coffea canephora
-1 12 -7 -6 -2 -13 -4 8 -11 -3 -5 -10 14 -9 15
-1 12 -7 -6 -2 -13 -4 8 -11 -3 -5 -10 9 14 15
-1 12 -7 -6 -2 -13 -4 8 9 -11 -3 -5 -10 14 15
-11 -3 -1 12 -7 -6 -2 -13 -4 8 9 -5 -10 14 15
1 3 11 12 -7 -6 -2 -4 -13 8 9 -5 -10 14 15
Vitis vinifera (post γ eudicot)
1 3 5 7 10 11 12 9 -8 13 4 2 6 14* 15*
1 3 5 9 -8 13 4 2 6 7 10 11 12 14 15
1 3 5 9 -8 13 4 2 6 7 -12 -11 -10 14 15
four translocations
one inversion
one transposition
Gentianales ancestor
1 3 5 -9 -8 13 4 2 6 7 -12 -11 -10 14 15
one translocation
one inversion
two fusions
three translocations
one inversion
one transposition
1 3 5 -9 -8 -7 -6 -2 -4 -13 -12 -11 -10 14 15
1 3 5 4 2 6 7 8 9 10 11 12 13 14 15
1 3 4 2 6 7 8 9 5 10 11 12 13 14 15
1 2 3 4 5 6 7 8 9 10 11 12 13 14 15
Rhazya stricta
Rss7 Rss9 Rss10 C1 C2 C3 C4 C5 C6 C7 C8 C9 C10 C11
14,9
15
1,12,7,
6,2,13,4,8
11,3,5,10
Rss7 Rss9 Rss10 V1 V2 V3 V4 V5 V6 V8 V9 V10 V11 V12 V13 V14 V15 V16 V17 V18 V19
1,3,5,7,
10,11,12
9
14
15
8,13,4,2,6

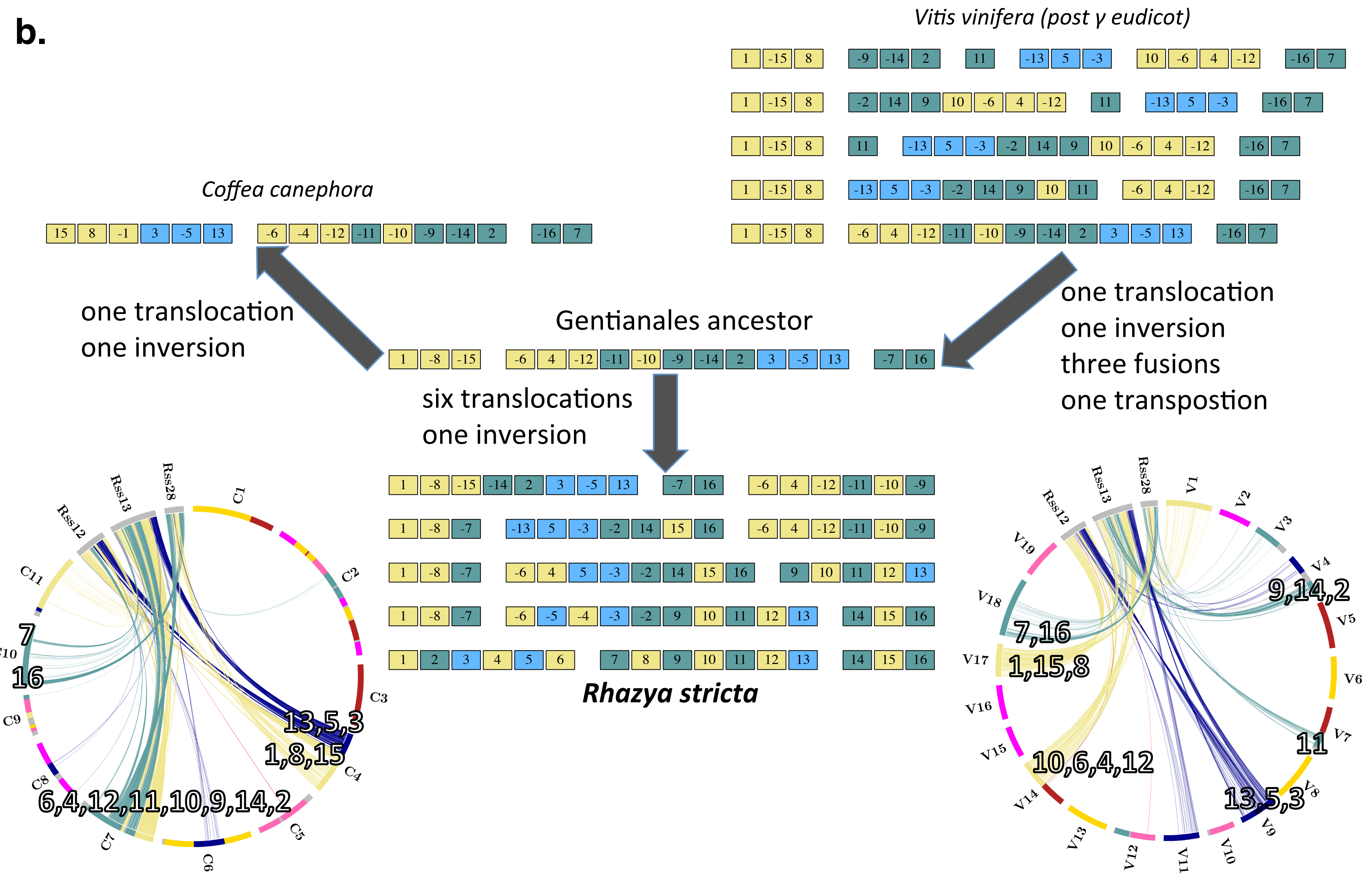

b.
Vitis vinifera (post γ eudicot)
1 -15 8 -9 -14 2 11 -13 5 -3 10 -6 4 -12 -16 7
1 -15 8 -2 14 9 10 -6 4 -12 11 -13 5 -3 -16 7
1 -15 8 11 -13 5 -3 -2 14 9 10 -6 4 -12 -16 7
1 -15 8 -13 5 -3 -2 14 9 10 11 -6 4 -12 -16 7
1 -15 8 -6 4 -12 -11 -10 -9 -14 2 3 -5 13 -16 7
Coffea canephora
15 8 -1 3 -5 13 -6 -4 -12 -11 -10 -9 -14 2 -16 7
one translocation
one inversion
Gentianales ancestor
1 -8 -15 -6 4 -12 -11 -10 -9 -14 2 3 -5 13 -7 16
one translocation
one inversion
three fusions
one transpostion
six translocations
one inversion
1 -8 -15 -14 2 3 -5 13 -7 16 -6 4 -12 -11 -10 -9
1 -8 -7 -13 5 -3 -2 14 15 16 -6 4 -12 -11 -10 -9
1 -8 -7 -6 4 5 -3 -2 14 15 16 9 10 11 12 13
1 -8 -7 -6 -5 -4 -3 -2 9 10 11 12 13 14 15 16
1 2 3 4 5 6 7 8 9 10 11 12 13 14 15 16
Rhazya stricta
Rss12 Rss13 Rss28 C1 C2 C3 C4 C5 C6 C7 C8 C9 C10 C11
7
16
13,5,3
1,8,15
6,4,12,11,10,9,14,2
Rss12 Rss13 Rss28 V1 V2 V3 V4 V5 V6 V7 V8 V9 V10 V11 V12 V13 V14 V15 V16 V17 V18 V19
9,14,2
7,16
1,15,8
11
10,6,4,12
13,5,3

a. 7DLS

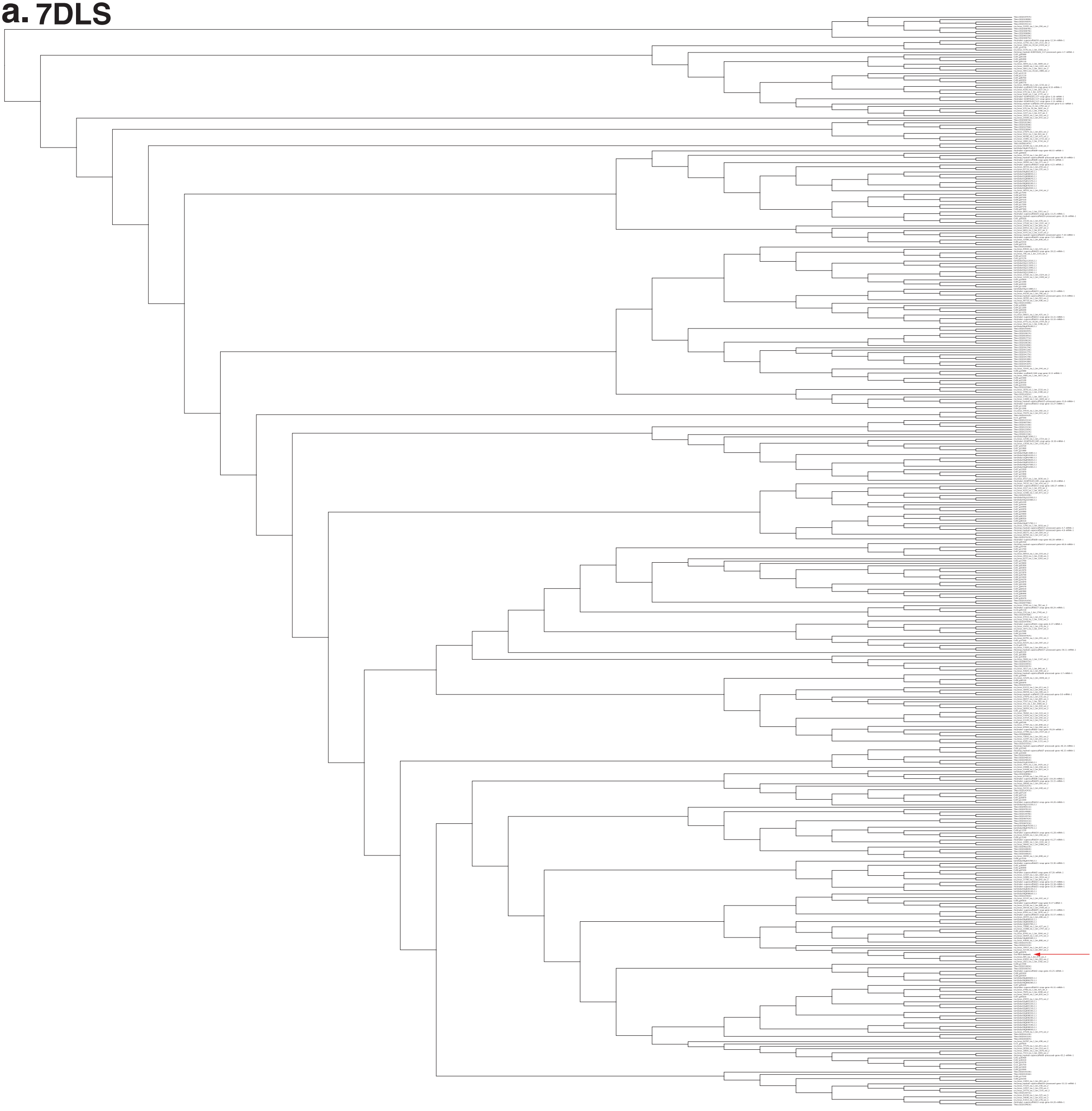

b. 160MT

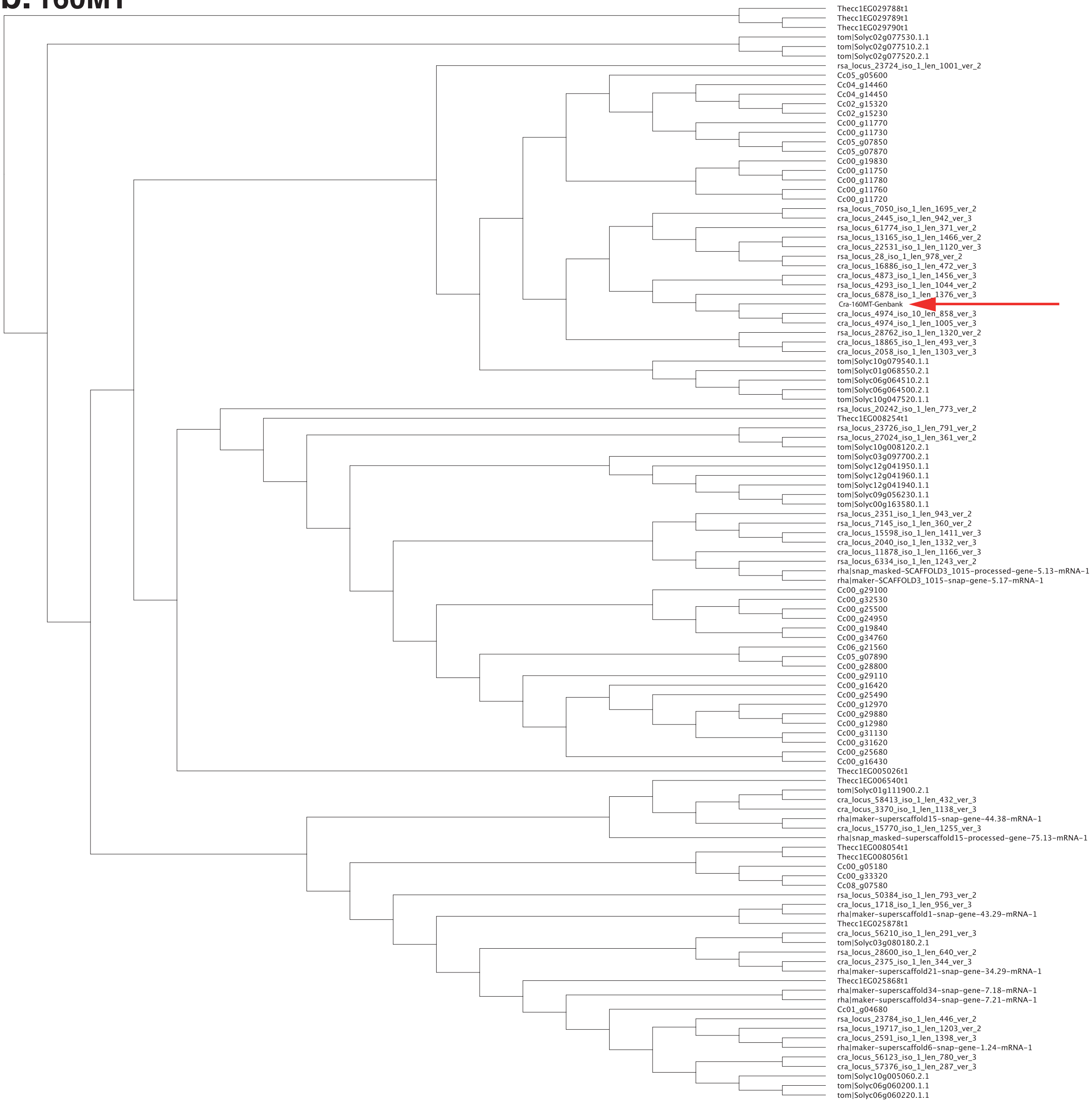
Thecc1EG029788t1
Thecc1EG029789t1
Thecc1EG029790t1
tom|Solyc02g077530.1.1
tom|Solyc02g077510.2.1
tom|Solyc02g077520.2.1
rsa_locus_23724_iso_1_len_1001_ver_2
Cc05_g05600
Cc04_g14460
Cc04_g14450
Cc02_g15320
Cc02_g15230
Cc00_g11770
Cc00_g11730
Cc05_g07850
Cc05_g07870
Cc00_g19830
Cc00_g11750
Cc00_g11780
Cc00_g11760
Cc00_g11720
rsa_locus_7050_iso_1_len_1695_ver_2
cra_locus_2445_iso_1_len_942_ver_3
rsa_locus_61774_iso_1_len_371_ver_2
rsa_locus_13165_iso_1_len_1466_ver_2
cra_locus_22531_iso_1_len_1120_ver_3
rsa_locus_28_iso_1_len_978_ver_2
cra_locus_16886_iso_1_len_472_ver_3
cra_locus_4873_iso_1_len_1456_ver_3
rsa_locus_4293_iso_1_len_1044_ver_2
cra_locus_6878_iso_1_len_1376_ver_3
Cra-160MT-Genbank
cra_locus_4974_iso_10_len_858_ver_3
cra_locus_4974_iso_1_len_1005_ver_3
rsa_locus_28762_iso_1_len_1320_ver_2
cra_locus_18865_iso_1_len_493_ver_3
cra_locus_2058_iso_1_len_1303_ver_3
tom|Solyc10g079540.1.1
tom|Solyc01g068550.2.1
tom|Solyc06g064510.2.1
tom|Solyc06g064500.2.1
tom|Solyc10g047520.1.1
rsa_locus_20242_iso_1_len_773_ver_2
Thecc1EG008254t1
rsa_locus_23726_iso_1_len_791_ver_2
rsa_locus_27024_iso_1_len_361_ver_2
tom|Solyc10g008120.2.1
tom|Solyc03g097700.2.1
tom|Solyc12g041950.1.1
tom|Solyc12g041960.1.1
tom|Solyc12g041940.1.1
tom|Solyc09g056230.1.1
tom|Solyc00g163580.1.1
rsa_locus_2351_iso_1_len_943_ver_2
rsa_locus_7145_iso_1_len_360_ver_2
cra_locus_15598_iso_1_len_1411_ver_3
cra_locus_2040_iso_1_len_1332_ver_3
cra_locus_11878_iso_1_len_1166_ver_3
rsa_locus_6334_iso_1_len_1243_ver_2
rha|snap_masked-SCAFFOLD3_1015-processed-gene-5.13-mRNA-1
rha|maker-SCAFFOLD3_1015-snap-gene-5.17-mRNA-1
Cc00_g29100
Cc00_g32530
Cc00_g25500
Cc00_g24950
Cc00_g19840
Cc00_g34760
Cc06_g21560
Cc05_g07890
Cc00_g28800
Cc00_g29110
Cc00_g16420
Cc00_g25490
Cc00_g12970
Cc00_g29880
Cc00_g12980
Cc00_g31130
Cc00_g31620
Cc00_g25680
Cc00_g16430
Thecc1EG005026t1
Thecc1EG006540t1
tom|Solyc01g111900.2.1
cra_locus_58413_iso_1_len_432_ver_3
cra_locus_3370_iso_1_len_1138_ver_3
rha|maker-superscaffold15-snap-gene-44.38-mRNA-1
cra_locus_15770_iso_1_len_1255_ver_3
rha|snap_masked-superscaffold15-processed-gene-75.13-mRNA-1
Thecc1EG008054t1
Thecc1EG008056t1
Cc00_g05180
Cc00_g33320
Cc08_g07580
rsa_locus_50384_iso_1_len_793_ver_2
cra_locus_1718_iso_1_len_956_ver_3
rha|maker-superscaffold1-snap-gene-43.29-mRNA-1
Thecc1EG025878t1
cra_locus_56210_iso_1_len_291_ver_3
tom|Solyc03g080180.2.1
rsa_locus_28600_iso_1_len_640_ver_2
cra_locus_2375_iso_1_len_344_ver_3
rha|maker-superscaffold21-snap-gene-34.29-mRNA-1
Thecc1EG025868t1
rha|maker-superscaffold34-snap-gene-7.18-mRNA-1
rha|maker-superscaffold34-snap-gene-7.21-mRNA-1
Cc01_g04680
rsa_locus_23784_iso_1_len_446_ver_2
rsa_locus_19717_iso_1_len_1203_ver_2
cra_locus_2591_iso_1_len_1398_ver_3
rha|maker-superscaffold6-snap-gene-1.24-mRNA-1
cra_locus_56123_iso_1_len_780_ver_3
cra_locus_57376_iso_1_len_287_ver_3
tom|Solyc10g005060.2.1
tom|Solyc06g060200.1.1
tom|Solyc06g060220.1.1

c. AAE

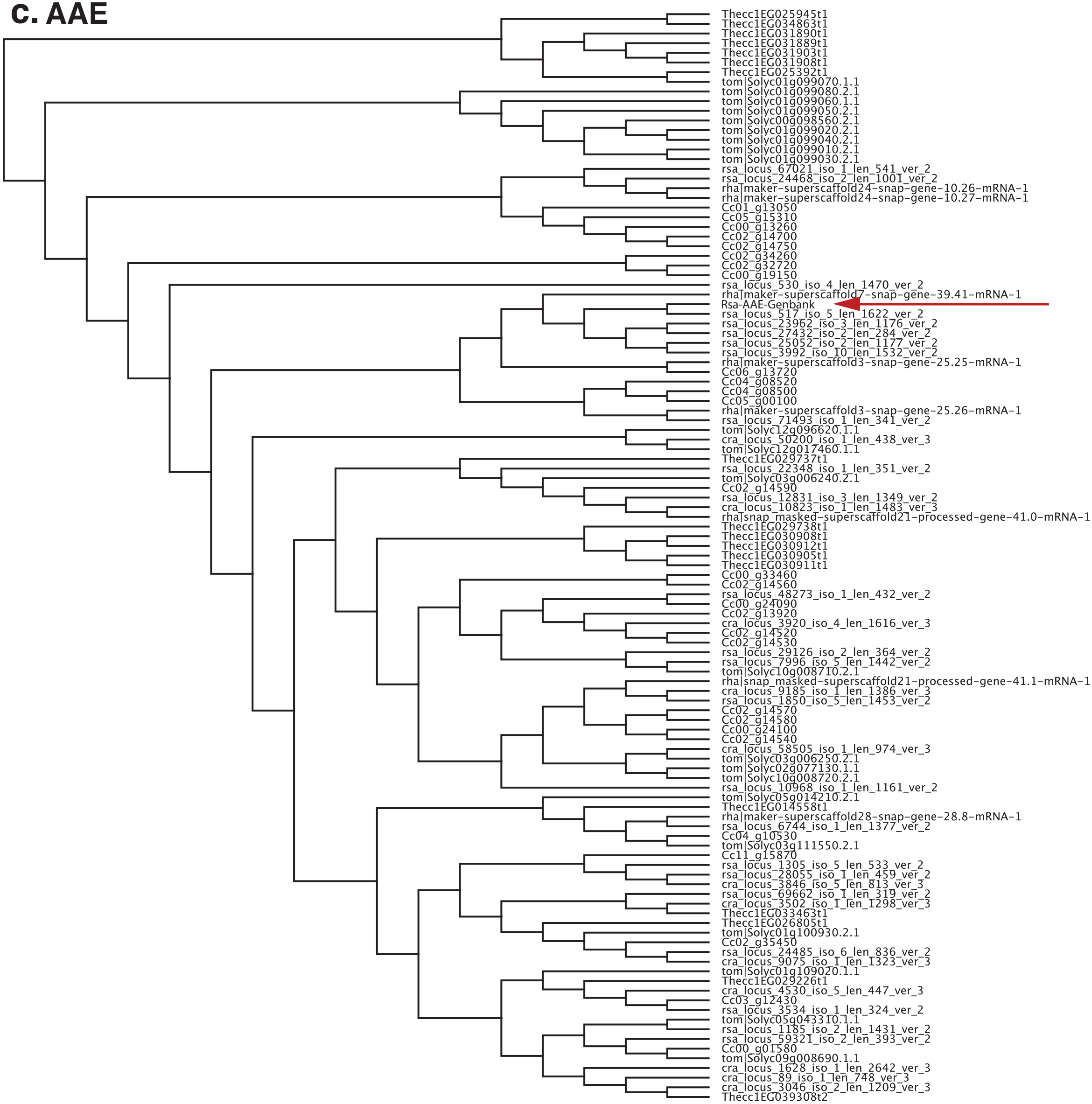
Thecc1EG025945t1
Thecc1EG034863t1
Thecc1EG031890t1
Thecc1EG031889t1
Thecc1EG031903t1
Thecc1EG031908t1
Thecc1EG025392t1
tom|Solyc01g099070.1.1
tom|Solyc01g099080.2.1
tom|Solyc01g099060.1.1
tom|Solyc01g099050.2.1
tom|Solyc00g098560.2.1
tom|Solyc01g099020.2.1
tom|Solyc01g099040.2.1
tom|Solyc01g099010.2.1
tom|Solyc01g099030.2.1
rsa_locus_67021_iso_1_len_541_ver_2
rsa_locus_24468_iso_2_len_1001_ver_2
rha|maker-superscaffold24-snap-gene-10.26-mRNA-1
rha|maker-superscaffold24-snap-gene-10.27-mRNA-1
Cc01_g13050
Cc05_g15310
Cc00_g13260
Cc02_g14700
Cc02_g14750
Cc02_g34260
Cc02_g32720
Cc00_g19150
rsa_locus_530_iso_4_len_1470_ver_2
rha|maker-superscaffold7-snap-gene-39.41-mRNA-1
Rsa-AAE-Genbank
rsa_locus_517_iso_5_len_1622_ver_2
rsa_locus_23962_iso_3_len_1176_ver_2
rsa_locus_27432_iso_2_len_284_ver_2
rsa_locus_25052_iso_2_len_1177_ver_2
rsa_locus_3992_iso_10_len_1532_ver_2
rha|maker-superscaffold3-snap-gene-25.25-mRNA-1
Cc06_g13720
Cc04_g08520
Cc04_g08500
Cc05_g00100
rha|maker-superscaffold3-snap-gene-25.26-mRNA-1
rsa_locus_71493_iso_1_len_341_ver_2
tom|Solyc12g096620.1.1
cra_locus_50200_iso_1_len_438_ver_3
tom|Solyc12g017460.1.1
Thecc1EG029737t1
rsa_locus_22348_iso_1_len_351_ver_2
tom|Solyc03g006240.2.1
Cc02_g14590
rsa_locus_12831_iso_3_len_1349_ver_2
cra_locus_10823_iso_1_len_1483_ver_3
rha|snap_masked-superscaffold21-processed-gene-41.0-mRNA-1
Thecc1EG029738t1
Thecc1EG030908t1
Thecc1EG030912t1
Thecc1EG030905t1
Thecc1EG030911t1
Cc00_g33460
Cc02_g14560
rsa_locus_48273_iso_1_len_432_ver_2
Cc00_g24090
Cc02_g13920
cra_locus_3920_iso_4_len_1616_ver_3
Cc02_g14520
Cc02_g14530
rsa_locus_29126_iso_2_len_364_ver_2
rsa_locus_7996_iso_5_len_1442_ver_2
tom|Solyc10g008710.2.1
rha|snap_masked-superscaffold21-processed-gene-41.1-mRNA-1
cra_locus_9185_iso_1_len_1386_ver_3
rsa_locus_1850_iso_5_len_1453_ver_2
Cc02_g14570
Cc02_g14580
Cc00_g24100
Cc02_g14540
cra_locus_58505_iso_1_len_974_ver_3
tom|Solyc03g006250.2.1
tom|Solyc02g077130.1.1
tom|Solyc10g008720.2.1
rsa_locus_10968_iso_1_len_1161_ver_2
tom|Solyc05g014210.2.1
Thecc1EG014558t1
rha|maker-superscaffold28-snap-gene-28.8-mRNA-1
rsa_locus_6744_iso_1_len_1377_ver_2
Cc04_g10530
tom|Solyc03g111550.2.1
Cc11_g15870
rsa_locus_1305_iso_5_len_533_ver_2
rsa_locus_28055_iso_1_len_459_ver_2
cra_locus_3846_iso_5_len_813_ver_3
rsa_locus_69662_iso_1_len_319_ver_2
cra_locus_3502_iso_1_len_1298_ver_3
Thecc1EG033463t1
Thecc1EG026805t1
tom|Solyc01g100930.2.1
Cc02_g35450
rsa_locus_24485_iso_6_len_836_ver_2
cra_locus_9075_iso_1_len_1323_ver_3
tom|Solyc01g109020.1.1
Thecc1EG029226t1
cra_locus_4530_iso_5_len_447_ver_3
Cc03_g12430
rsa_locus_3534_iso_1_len_324_ver_2
tom|Solyc05g043310.1.1
rsa_locus_1185_iso_2_len_1431_ver_2
rsa_locus_59321_iso_2_len_393_ver_2
Cc00_g01580
tom|Solyc09g008690.1.1
cra_locus_1628_iso_1_len_2642_ver_3
cra_locus_89_iso_1_len_748_ver_3
cra_locus_3046_iso_2_len_1209_ver_3
Thecc1EG039308t2

d. DAT

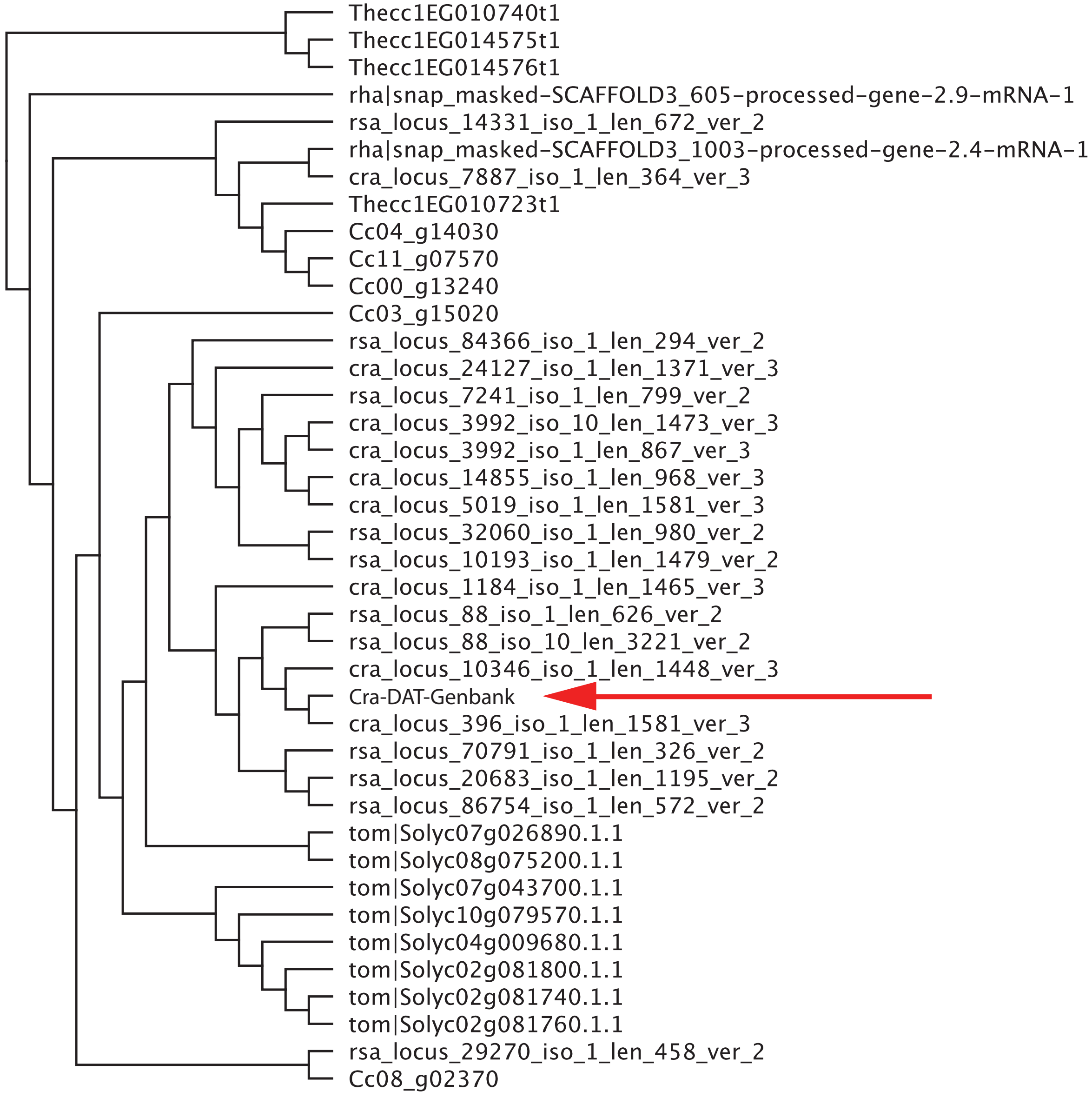
Thecc1EG010740t1
Thecc1EG014575t1
Thecc1EG014576t1
rha|snap_masked-SCAFFOLD3_605-processed-gene-2.9-mRNA-1
rsa_locus_14331_iso_1_len_672_ver_2
rha|snap_masked-SCAFFOLD3_1003-processed-gene-2.4-mRNA-1
cra_locus_7887_iso_1_len_364_ver_3
Thecc1EG010723t1
Cc04_g14030
Cc11_g07570
Cc00_g13240
Cc03_g15020
rsa_locus_84366_iso_1_len_294_ver_2
cra_locus_24127_iso_1_len_1371_ver_3
rsa_locus_7241_iso_1_len_799_ver_2
cra_locus_3992_iso_10_len_1473_ver_3
cra_locus_3992_iso_1_len_867_ver_3
cra_locus_14855_iso_1_len_968_ver_3
cra_locus_5019_iso_1_len_1581_ver_3
rsa_locus_32060_iso_1_len_980_ver_2
rsa_locus_10193_iso_1_len_1479_ver_2
cra_locus_1184_iso_1_len_1465_ver_3
rsa_locus_88_iso_1_len_626_ver_2
rsa_locus_88_iso_10_len_3221_ver_2
cra_locus_10346_iso_1_len_1448_ver_3
Cra-DAT-Genbank
cra_locus_396_iso_1_len_1581_ver_3
rsa_locus_70791_iso_1_len_326_ver_2
rsa_locus_20683_iso_1_len_1195_ver_2
rsa_locus_86754_iso_1_len_572_ver_2
tom|Solyc07g026890.1.1
tom|Solyc08g075200.1.1
tom|Solyc07g043700.1.1
tom|Solyc10g079570.1.1
tom|Solyc04g009680.1.1
tom|Solyc02g081800.1.1
tom|Solyc02g081740.1.1
tom|Solyc02g081760.1.1
rsa_locus_29270_iso_1_len_458_ver_2
Cc08_g02370

e. D4H

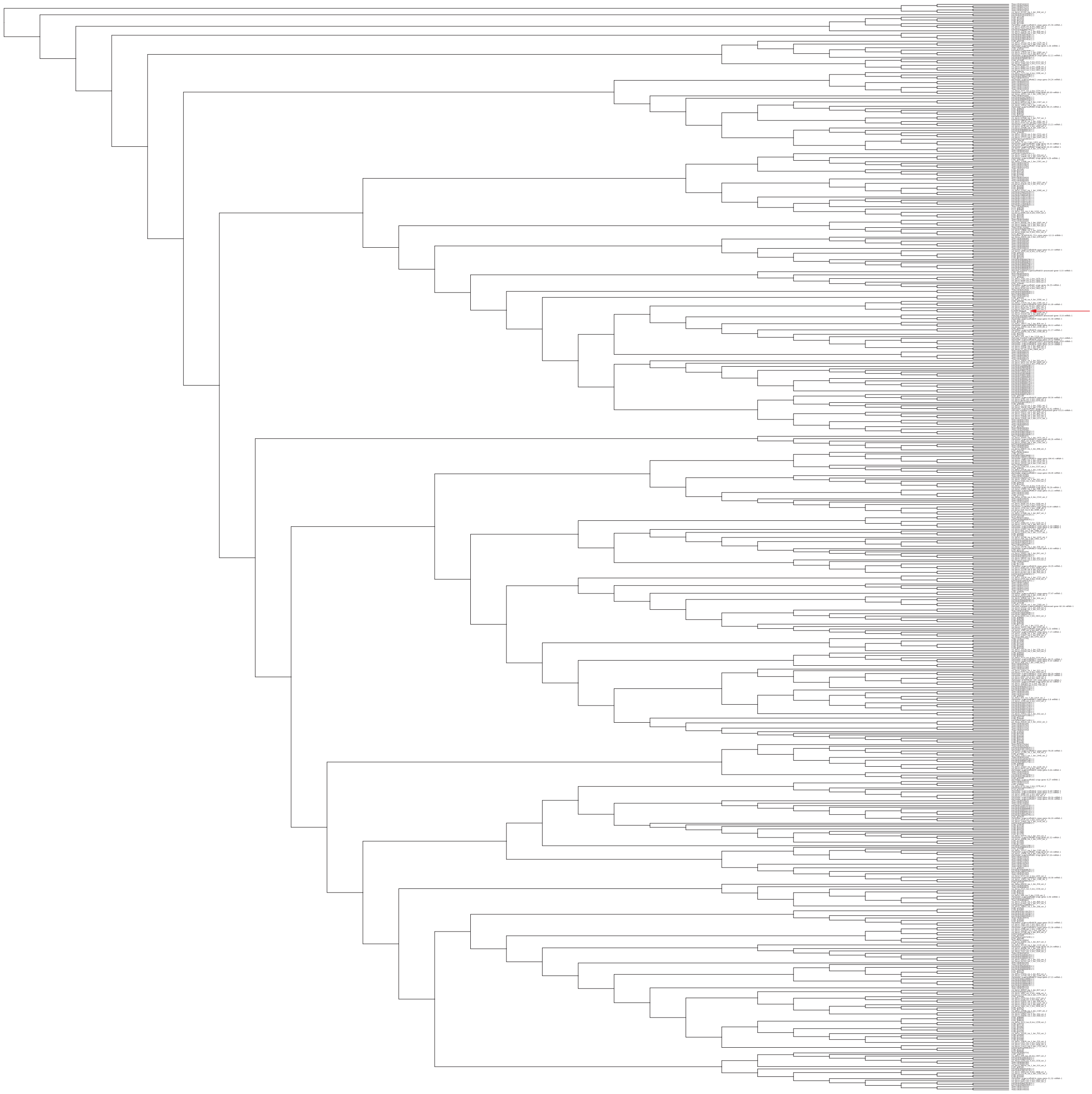

f. G10H

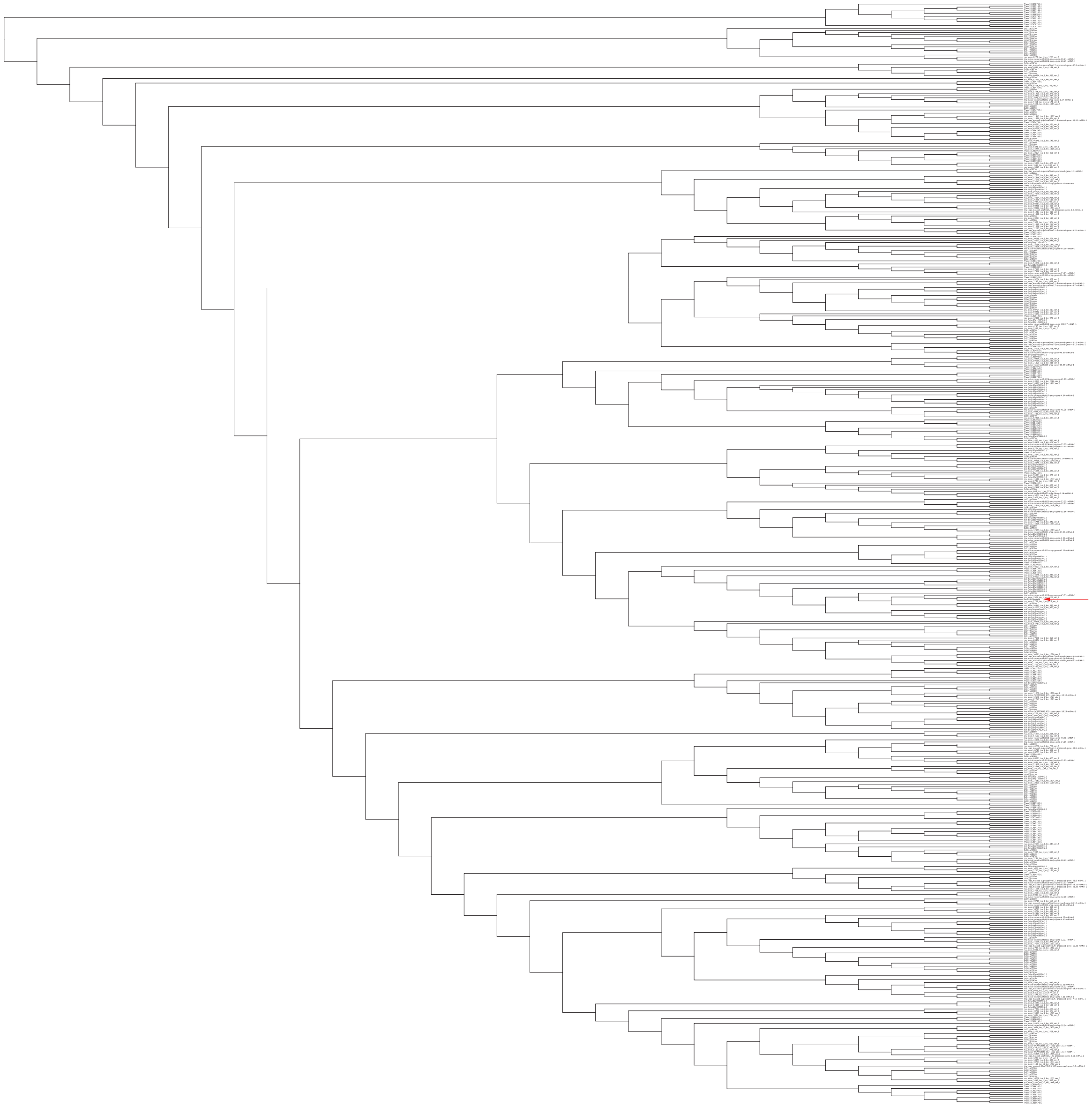

g. LAMT

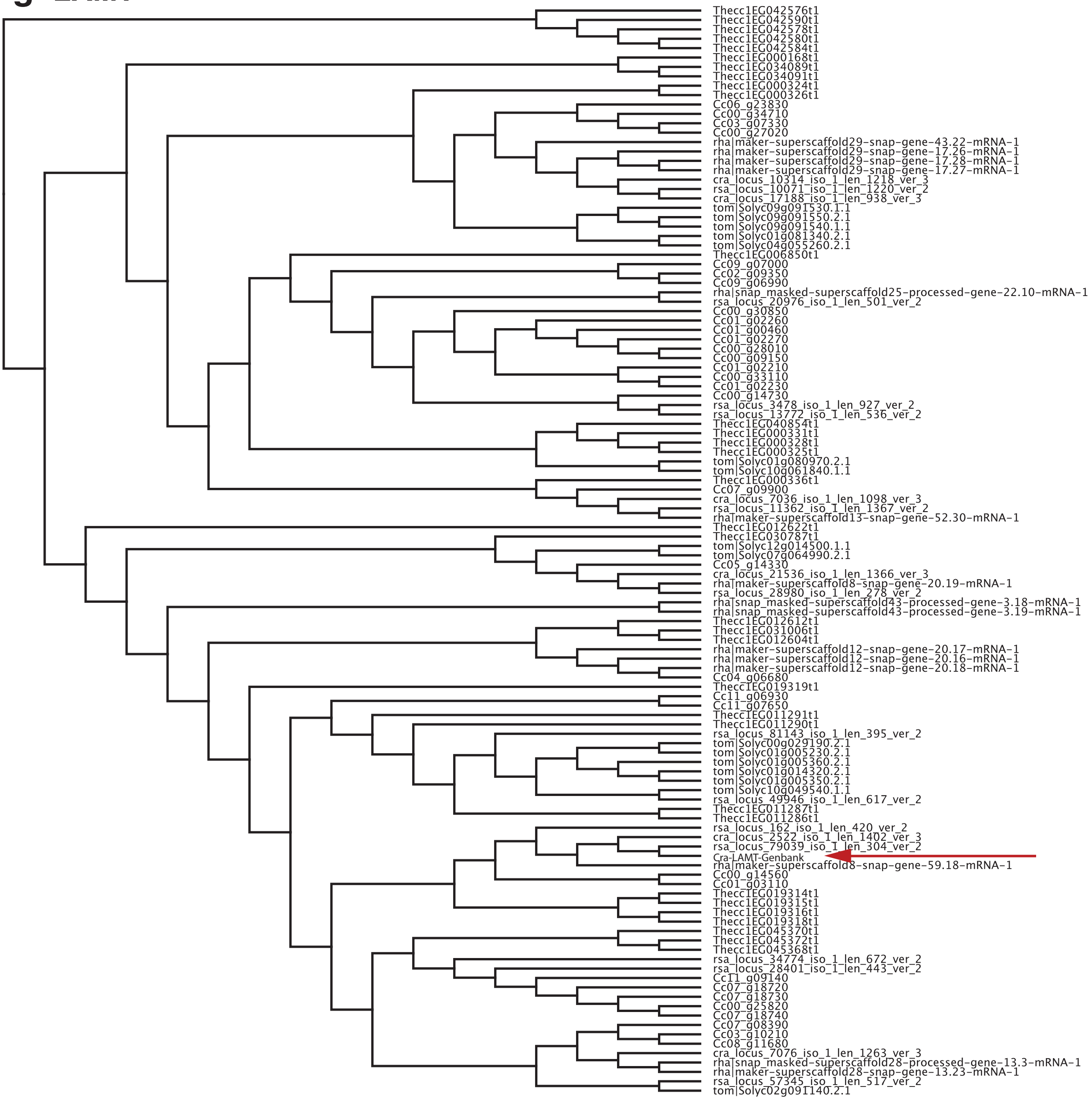
Thecc1EG042576t1
Thecc1EG042590t1
Thecc1EG042578t1
Thecc1EG042580t1
Thecc1EG042584t1
Thecc1EG000168t1
Thecc1EG034089t1
Thecc1EG034091t1
Thecc1EG000324t1
Thecc1EG000326t1
Cc06_g23830
Cc00_g34710
Cc03_g07330
Cc00_g27020
rha|maker-superscaffold29-snap-gene-43.22-mRNA-1
rha|maker-superscaffold29-snap-gene-17.26-mRNA-1
rha|maker-superscaffold29-snap-gene-17.28-mRNA-1
rha|maker-superscaffold29-snap-gene-17.27-mRNA-1
cra_locus_10314_iso_1_len_1218_ver_3
rsa_locus_10071_iso_1_len_1220_ver_2
cra_locus_17188_iso_1_len_938_ver_3
tom|Solyc09g091530.1.1
tom|Solyc09g091550.2.1
tom|Solyc09g091540.1.1
tom|Solyc01g081340.2.1
tom|Solyc04g055260.2.1
Thecc1EG006850t1
Cc09_g07000
Cc02_g09350
Cc09_g06990
rha|snap_masked-superscaffold25-processed-gene-22.10-mRNA-1
rsa_locus_20976_iso_1_len_501_ver_2
Cc00_g30850
Cc01_g02260
Cc01_g00460
Cc01_g02270
Cc00_g28010
Cc00_g09150
Cc01_g02210
Cc00_g33110
Cc01_g02230
Cc00_g14730
rsa_locus_3478_iso_1_len_927_ver_2
rsa_locus_13772_iso_1_len_536_ver_2
Thecc1EG040854t1
Thecc1EG000331t1
Thecc1EG000328t1
Thecc1EG000325t1
tom|Solyc01g080970.2.1
tom|Solyc10g061840.1.1
Thecc1EG000336t1
Cc07_g09900
cra_locus_7036_iso_1_len_1098_ver_3
rsa_locus_11362_iso_1_len_1367_ver_2
rha|maker-superscaffold13-snap-gene-52.30-mRNA-1
Thecc1EG012622t1
Thecc1EG030787t1
tom|Solyc12g014500.1.1
tom|Solyc07g064990.2.1
Cc05_g14330
cra_locus_21536_iso_1_len_1366_ver_3
rha|maker-superscaffold8-snap-gene-20.19-mRNA-1
rsa_locus_28980_iso_1_len_278_ver_2
rha|snap_masked-superscaffold43-processed-gene-3.18-mRNA-1
rha|snap_masked-superscaffold43-processed-gene-3.19-mRNA-1
Thecc1EG012612t1
Thecc1EG031006t1
Thecc1EG012604t1
rha|maker-superscaffold12-snap-gene-20.17-mRNA-1
rha|maker-superscaffold12-snap-gene-20.16-mRNA-1
rha|maker-superscaffold12-snap-gene-20.18-mRNA-1
Cc04_g06680
Thecc1EG019319t1
Cc11_g06930
Cc11_g07650
Thecc1EG011291t1
Thecc1EG011290t1
rsa_locus_81143_iso_1_len_395_ver_2
tom|Solyc00g029190.2.1
tom|Solyc01g005230.2.1
tom|Solyc01g005360.2.1
tom|Solyc01g014320.2.1
tom|Solyc01g005350.2.1
tom|Solyc10g049540.1.1
rsa_locus_49946_iso_1_len_617_ver_2
Thecc1EG011287t1
Thecc1EG011286t1
rsa_locus_162_iso_1_len_420_ver_2
cra_locus_2522_iso_1_len_1402_ver_3
rsa_locus_79039_iso_1_len_304_ver_2
Cra-LAMT-Genbank
rha|maker-superscaffold8-snap-gene-59.18-mRNA-1
Cc00_g14560
Cc01_g03110
Thecc1EG019314t1
Thecc1EG019315t1
Thecc1EG019316t1
Thecc1EG019318t1
Thecc1EG045370t1
Thecc1EG045372t1
Thecc1EG045368t1
rsa_locus_34774_iso_1_len_672_ver_2
rsa_locus_28401_iso_1_len_443_ver_2
Cc11_g09140
Cc07_g18720
Cc07_g18730
Cc00_g25820
Cc07_g18740
Cc07_g08390
Cc03_g10210
Cc08_g11680
cra_locus_7076_iso_1_len_1263_ver_3
rha|snap_masked-superscaffold28-processed-gene-13.3-mRNA-1
rha|maker-superscaffold28-snap-gene-13.23-mRNA-1
rsa_locus_57345_iso_1_len_517_ver_2
tom|Solyc02g091140.2.1

h. MAT

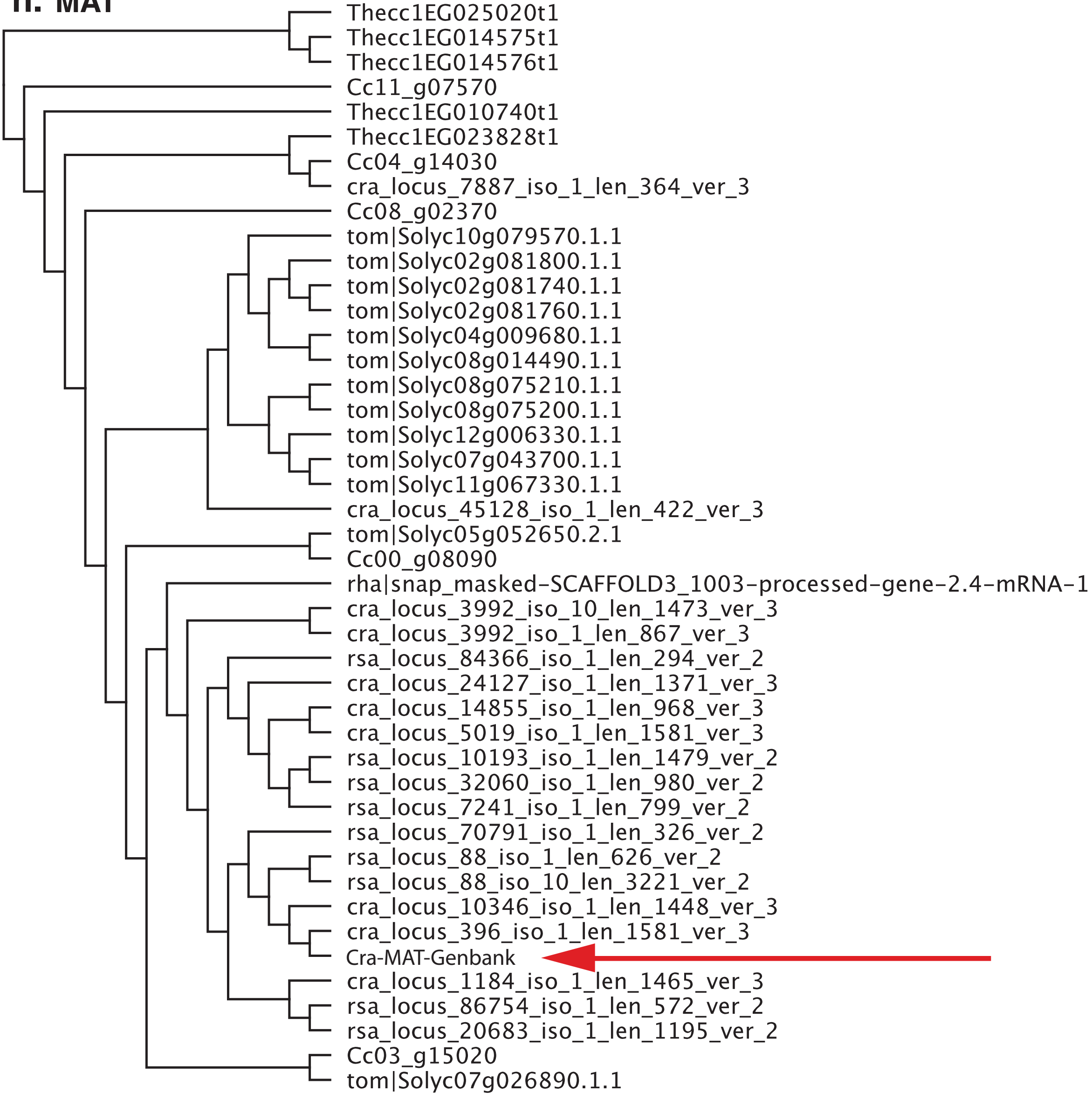

Thecc1EG025020t1
Thecc1EG014575t1
Thecc1EG014576t1
Cc11_g07570
Thecc1EG010740t1
Thecc1EG023828t1
Cc04_g14030
cra_locus_7887_iso_1_len_364_ver_3
Cc08_g02370
tom|Solyc10g079570.1.1
tom|Solyc02g081800.1.1
tom|Solyc02g081740.1.1
tom|Solyc02g081760.1.1
tom|Solyc04g009680.1.1
tom|Solyc08g014490.1.1
tom|Solyc08g075210.1.1
tom|Solyc08g075200.1.1
tom|Solyc12g006330.1.1
tom|Solyc07g043700.1.1
tom|Solyc11g067330.1.1
cra_locus_45128_iso_1_len_422_ver_3
tom|Solyc05g052650.2.1
Cc00_g08090
rha|snap_masked-SCAFFOLD3_1003-processed-gene-2.4-mRNA-1
cra_locus_3992_iso_10_len_1473_ver_3
cra_locus_3992_iso_1_len_867_ver_3
rsa_locus_84366_iso_1_len_294_ver_2
cra_locus_24127_iso_1_len_1371_ver_3
cra_locus_14855_iso_1_len_968_ver_3
cra_locus_5019_iso_1_len_1581_ver_3
rsa_locus_10193_iso_1_len_1479_ver_2
rsa_locus_32060_iso_1_len_980_ver_2
rsa_locus_7241_iso_1_len_799_ver_2
rsa_locus_70791_iso_1_len_326_ver_2
rsa_locus_88_iso_1_len_626_ver_2
rsa_locus_88_iso_10_len_3221_ver_2
cra_locus_10346_iso_1_len_1448_ver_3
cra_locus_396_iso_1_len_1581_ver_3
Cra-MAT-Genbank
cra_locus_1184_iso_1_len_1465_ver_3
rsa_locus_86754_iso_1_len_572_ver_2
rsa_locus_20683_iso_1_len_1195_ver_2
Cc03_g15020
tom|Solyc07g026890.1.1

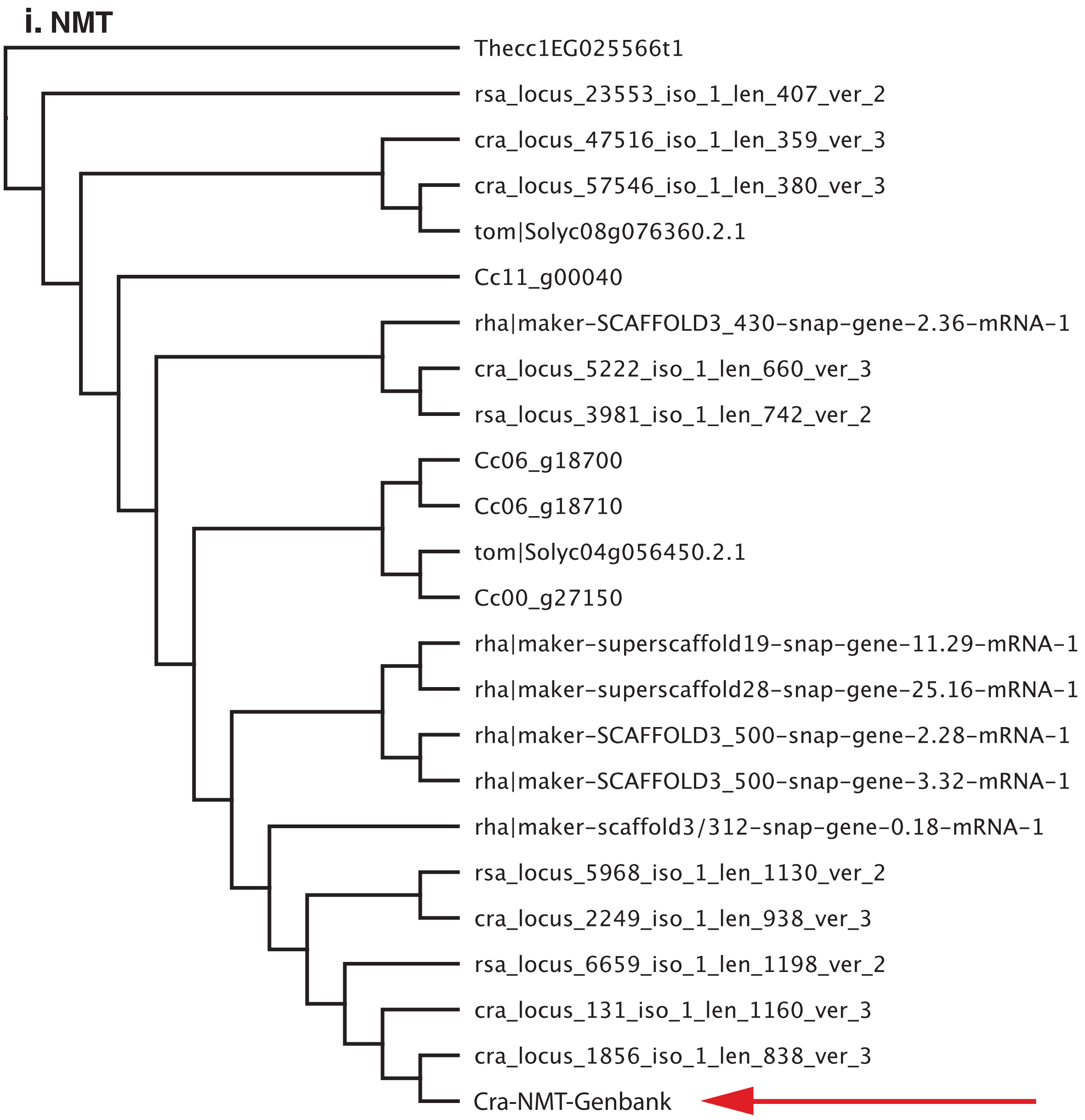
i. NMT
Thecc1EG025566t1
rsa_locus_23553_iso_1_len_407_ver_2
cra_locus_47516_iso_1_len_359_ver_3
cra_locus_57546_iso_1_len_380_ver_3
tom|Solyc08g076360.2.1
Cc11_g00040
rha|maker-SCAFFOLD3_430-snap-gene-2.36-mRNA-1
cra_locus_5222_iso_1_len_660_ver_3
rsa_locus_3981_iso_1_len_742_ver_2
Cc06_g18700
Cc06_g18710
tom|Solyc04g056450.2.1
Cc00_g27150
rha|maker-superscaffold19-snap-gene-11.29-mRNA-1
rha|maker-superscaffold28-snap-gene-25.16-mRNA-1
rha|maker-SCAFFOLD3_500-snap-gene-2.28-mRNA-1
rha|maker-SCAFFOLD3_500-snap-gene-3.32-mRNA-1
rha|maker-scaffold3/312-snap-gene-0.18-mRNA-1
rsa_locus_5968_iso_1_len_1130_ver_2
cra_locus_2249_iso_1_len_938_ver_3
rsa_locus_6659_iso_1_len_1198_ver_2
cra_locus_131_iso_1_len_1160_ver_3
cra_locus_1856_iso_1_len_838_ver_3
Cra-NMT-Genbank

j. PNAE

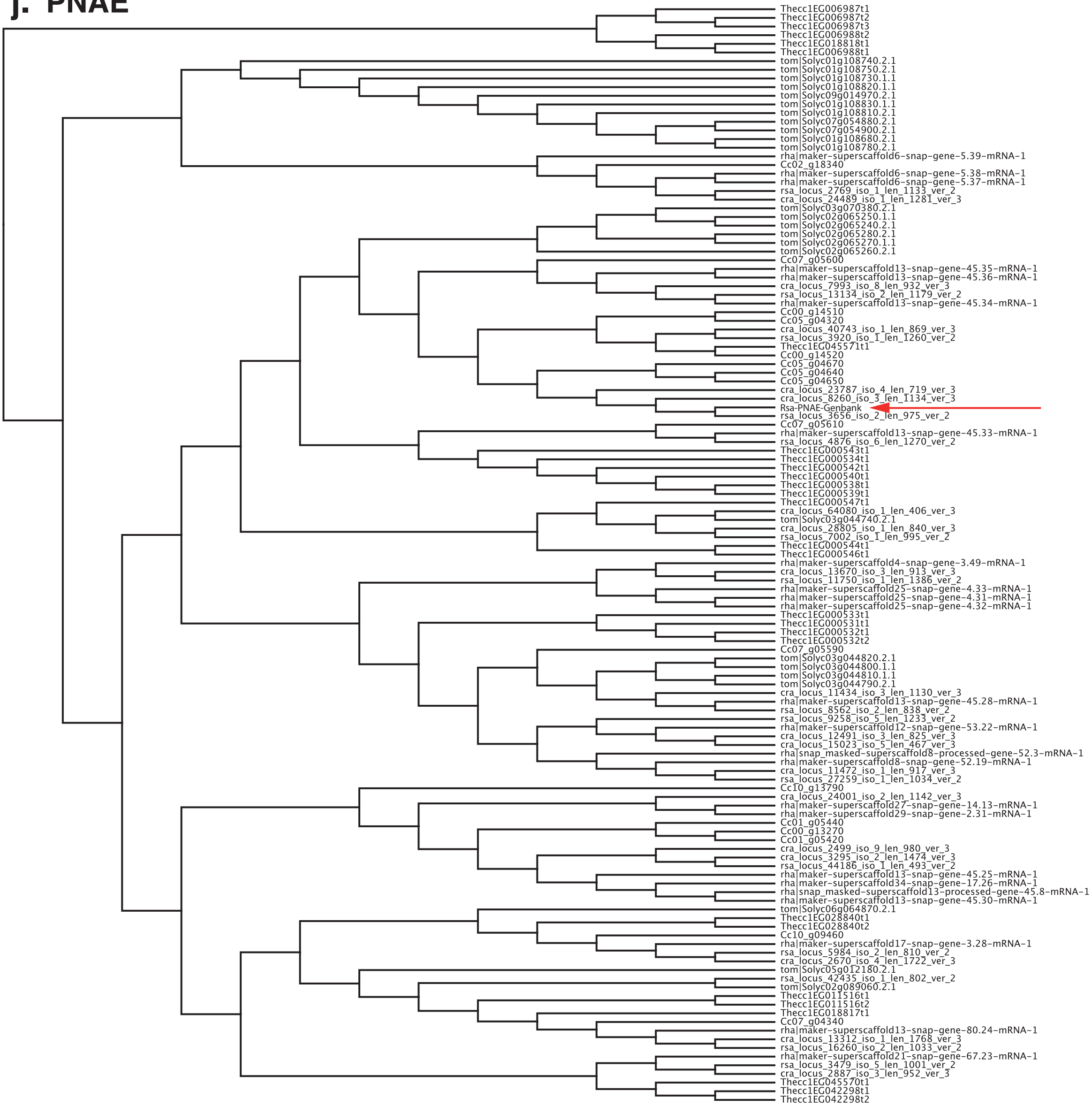
Thecc1EG006987t1
Thecc1EG006987t2
Thecc1EG006987t3
Thecc1EG006988t2
Thecc1EG018818t1
Thecc1EG006988t1
tom|Solyc01g108740.2.1
tom|Solyc01g108750.2.1
tom|Solyc01g108730.1.1
tom|Solyc01g108820.1.1
tom|Solyc09g014970.2.1
tom|Solyc01g108830.1.1
tom|Solyc01g108810.2.1
tom|Solyc07g054880.2.1
tom|Solyc07g054900.2.1
tom|Solyc01g108680.2.1
tom|Solyc01g108780.2.1
rha|maker-superscaffold6-snap-gene-5.39-mRNA-1
Cc02_g18340
rha|maker-superscaffold6-snap-gene-5.38-mRNA-1
rha|maker-superscaffold6-snap-gene-5.37-mRNA-1
rsa_locus_2769_iso_1_len_1133_ver_2
cra_locus_24489_iso_1_len_1281_ver_3
tom|Solyc03g070380.2.1
tom|Solyc02g065250.1.1
tom|Solyc02g065240.2.1
tom|Solyc02g065280.2.1
tom|Solyc02g065270.1.1
tom|Solyc02g065260.2.1
Cc07_g05600
rha|maker-superscaffold13-snap-gene-45.35-mRNA-1
rha|maker-superscaffold13-snap-gene-45.36-mRNA-1
cra_locus_7993_iso_8_len_932_ver_3
rsa_locus_13134_iso_2_len_1179_ver_2
rha|maker-superscaffold13-snap-gene-45.34-mRNA-1
Cc00_g14510
Cc05_g04320
cra_locus_40743_iso_1_len_869_ver_3
rsa_locus_3920_iso_1_len_1260_ver_2
Thecc1EG045571t1
Cc00_g14520
Cc05_g04670
Cc05_g04640
Cc05_g04650
cra_locus_23787_iso_4_len_719_ver_3
cra_locus_8260_iso_3_len_1134_ver_3
Rsa-PNAE-Genbank
rsa_locus_3656_iso_2_len_975_ver_2
Cc07_g05610
rha|maker-superscaffold13-snap-gene-45.33-mRNA-1
rsa_locus_4876_iso_6_len_1270_ver_2
Thecc1EG000543t1
Thecc1EG000534t1
Thecc1EG000542t1
Thecc1EG000540t1
Thecc1EG000538t1
Thecc1EG000539t1
Thecc1EG000547t1
cra_locus_64080_iso_1_len_406_ver_3
tom|Solyc03g044740.2.1
cra_locus_28805_iso_1_len_840_ver_3
rsa_locus_7002_iso_1_len_995_ver_2
Thecc1EG000544t1
Thecc1EG000546t1
rha|maker-superscaffold4-snap-gene-3.49-mRNA-1
cra_locus_13670_iso_3_len_913_ver_3
rsa_locus_11750_iso_1_len_1386_ver_2
rha|maker-superscaffold25-snap-gene-4.33-mRNA-1
rha|maker-superscaffold25-snap-gene-4.31-mRNA-1
rha|maker-superscaffold25-snap-gene-4.32-mRNA-1
Thecc1EG000533t1
Thecc1EG000531t1
Thecc1EG000532t1
Thecc1EG000532t2
Cc07_g05590
tom|Solyc03g044820.2.1
tom|Solyc03g044800.1.1
tom|Solyc03g044810.1.1
tom|Solyc03g044790.2.1
cra_locus_11434_iso_3_len_1130_ver_3
rha|maker-superscaffold13-snap-gene-45.28-mRNA-1
rsa_locus_8562_iso_2_len_838_ver_2
rsa_locus_9258_iso_5_len_1233_ver_2
rha|maker-superscaffold12-snap-gene-53.22-mRNA-1
cra_locus_12491_iso_3_len_825_ver_3
cra_locus_15023_iso_5_len_467_ver_3
rha|snap_masked-superscaffold8-processed-gene-52.3-mRNA-1
rha|maker-superscaffold8-snap-gene-52.19-mRNA-1
cra_locus_11472_iso_1_len_917_ver_3
rsa_locus_27259_iso_1_len_1034_ver_2
Cc10_g13790
cra_locus_24001_iso_2_len_1142_ver_3
rha|maker-superscaffold27-snap-gene-14.13-mRNA-1
rha|maker-superscaffold29-snap-gene-2.31-mRNA-1
Cc01_g05440
Cc00_g13270
Cc01_g05420
cra_locus_2499_iso_9_len_980_ver_3
cra_locus_3295_iso_2_len_1474_ver_3
rsa_locus_44186_iso_1_len_493_ver_2
rha|maker-superscaffold13-snap-gene-45.25-mRNA-1
rha|maker-superscaffold34-snap-gene-17.26-mRNA-1
rha|snap_masked-superscaffold13-processed-gene-45.8-mRNA-1
rha|maker-superscaffold13-snap-gene-45.30-mRNA-1
tom|Solyc06g064870.2.1
Thecc1EG028840t1
Thecc1EG028840t2
Cc10_g09460
rha|maker-superscaffold17-snap-gene-3.28-mRNA-1
rsa_locus_5984_iso_2_len_810_ver_2
cra_locus_2670_iso_4_len_1722_ver_3
tom|Solyc05g012180.2.1
rsa_locus_42435_iso_1_len_802_ver_2
tom|Solyc02g089060.2.1
Thecc1EG011516t1
Thecc1EG011516t2
Thecc1EG018817t1
Cc07_g04340
rha|maker-superscaffold13-snap-gene-80.24-mRNA-1
cra_locus_13312_iso_1_len_1768_ver_3
rsa_locus_16260_iso_2_len_1033_ver_2
rha|maker-superscaffold21-snap-gene-67.23-mRNA-1
rsa_locus_3479_iso_5_len_1001_ver_2
cra_locus_2887_iso_3_len_952_ver_3
Thecc1EG045570t1
Thecc1EG042298t1
Thecc1EG042298t2

k. PR

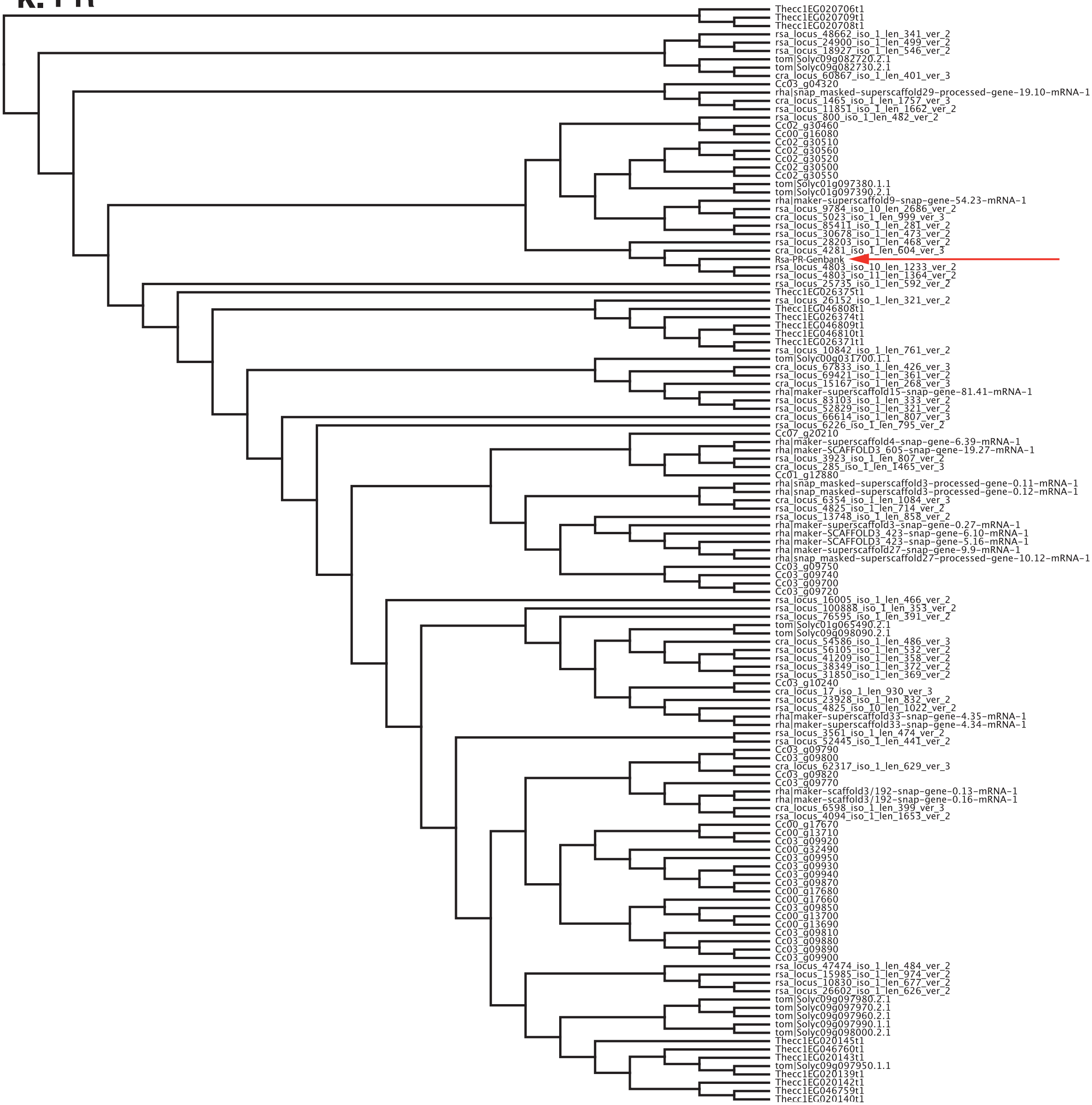
Thecc1EG020706t1
Thecc1EG020709t1
Thecc1EG020708t1
rsa_locus_48662_iso_1_len_341_ver_2
rsa_locus_24900_iso_1_len_499_ver_2
rsa_locus_18927_iso_1_len_546_ver_2
tom|Solyc09g082720.2.1
tom|Solyc09g082730.2.1
cra_locus_60867_iso_1_len_401_ver_3
Cc03_g04320
rha|snap_masked-superscaffold29-processed-gene-19.10-mRNA-1
cra_locus_1465_iso_1_len_1757_ver_3
rsa_locus_11851_iso_1_len_1662_ver_2
rsa_locus_800_iso_1_len_482_ver_2
Cc02_g30460
Cc00_g16080
Cc02_g30510
Cc02_g30560
Cc02_g30520
Cc02_g30500
Cc02_g30550
tom|Solyc01g097380.1.1
tom|Solyc01g097390.2.1
rha|maker-superscaffold9-snap-gene-54.23-mRNA-1
rsa_locus_9784_iso_10_len_2686_ver_2
cra_locus_5023_iso_1_len_999_ver_3
rsa_locus_85411_iso_1_len_281_ver_2
rsa_locus_30678_iso_1_len_473_ver_2
rsa_locus_28203_iso_1_len_468_ver_2
cra_locus_4281_iso_1_len_604_ver_3
Rsa-PR-Genbank
rsa_locus_4803_iso_10_len_1233_ver_2
rsa_locus_4803_iso_11_len_1364_ver_2
rsa_locus_25735_iso_1_len_592_ver_2
Thecc1EG026375t1
rsa_locus_26152_iso_1_len_321_ver_2
Thecc1EG046808t1
Thecc1EG026374t1
Thecc1EG046809t1
Thecc1EG046810t1
Thecc1EG026371t1
rsa_locus_10842_iso_1_len_761_ver_2
tom|Solyc00g031700.1.1
cra_locus_67833_iso_1_len_426_ver_3
rsa_locus_69421_iso_1_len_361_ver_2
cra_locus_15167_iso_1_len_268_ver_3
rha|maker-superscaffold15-snap-gene-81.41-mRNA-1
rsa_locus_83103_iso_1_len_333_ver_2
rsa_locus_52829_iso_1_len_321_ver_2
cra_locus_66614_iso_1_len_807_ver_3
rsa_locus_6226_iso_1_len_795_ver_2
Cc07_g20210
rha|maker-superscaffold4-snap-gene-6.39-mRNA-1
rha|maker-SCAFFOLD3_605-snap-gene-19.27-mRNA-1
rsa_locus_3923_iso_1_len_807_ver_2
cra_locus_285_iso_1_len_1465_ver_3
Cc01_g12880
rha|snap_masked-superscaffold3-processed-gene-0.11-mRNA-1
rha|snap_masked-superscaffold3-processed-gene-0.12-mRNA-1
cra_locus_6354_iso_1_len_1084_ver_3
rsa_locus_4825_iso_1_len_714_ver_2
rsa_locus_13748_iso_1_len_858_ver_2
rha|maker-superscaffold3-snap-gene-0.27-mRNA-1
rha|maker-SCAFFOLD3_423-snap-gene-6.10-mRNA-1
rha|maker-SCAFFOLD3_423-snap-gene-5.16-mRNA-1
rha|maker-superscaffold27-snap-gene-9.9-mRNA-1
rha|snap_masked-superscaffold27-processed-gene-10.12-mRNA-1
Cc03_g09750
Cc03_g09740
Cc03_g09700
Cc03_g09720
rsa_locus_16005_iso_1_len_466_ver_2
rsa_locus_100888_iso_1_len_353_ver_2
rsa_locus_76595_iso_1_len_391_ver_2
tom|Solyc01g065490.2.1
tom|Solyc09g098090.2.1
cra_locus_54586_iso_1_len_486_ver_3
rsa_locus_56105_iso_1_len_532_ver_2
rsa_locus_41209_iso_1_len_358_ver_2
rsa_locus_38349_iso_1_len_372_ver_2
rsa_locus_31850_iso_1_len_369_ver_2
Cc03_g10240
cra_locus_17_iso_1_len_930_ver_3
rsa_locus_23928_iso_1_len_832_ver_2
rsa_locus_4825_iso_10_len_1022_ver_2
rha|maker-superscaffold33-snap-gene-4.35-mRNA-1
rha|maker-superscaffold33-snap-gene-4.34-mRNA-1
rsa_locus_3561_iso_1_len_474_ver_2
rsa_locus_52445_iso_1_len_441_ver_2
Cc03_g09790
Cc03_g09800
cra_locus_62317_iso_1_len_629_ver_3
Cc03_g09820
Cc03_g09770
rha|maker-scaffold3/192-snap-gene-0.13-mRNA-1
rha|maker-scaffold3/192-snap-gene-0.16-mRNA-1
cra_locus_6598_iso_1_len_399_ver_3
rsa_locus_4094_iso_1_len_1653_ver_2
Cc00_g17670
Cc00_g13710
Cc03_g09920
Cc00_g32490
Cc03_g09950
Cc03_g09930
Cc03_g09940
Cc03_g09870
Cc00_g17680
Cc00_g17660
Cc03_g09850
Cc00_g13700
Cc00_g13690
Cc03_g09810
Cc03_g09880
Cc03_g09890
Cc03_g09900
rsa_locus_47474_iso_1_len_484_ver_2
rsa_locus_15985_iso_1_len_974_ver_2
rsa_locus_10830_iso_1_len_677_ver_2
rsa_locus_26602_iso_1_len_626_ver_2
tom|Solyc09g097980.2.1
tom|Solyc09g097970.2.1
tom|Solyc09g097960.2.1
tom|Solyc09g097990.1.1
tom|Solyc09g098000.2.1
Thecc1EG020145t1
Thecc1EG046760t1
Thecc1EG020143t1
tom|Solyc09g097950.1.1
Thecc1EG020139t1
Thecc1EG020142t1
Thecc1EG046759t1
Thecc1EG020140t1

I. PRX1

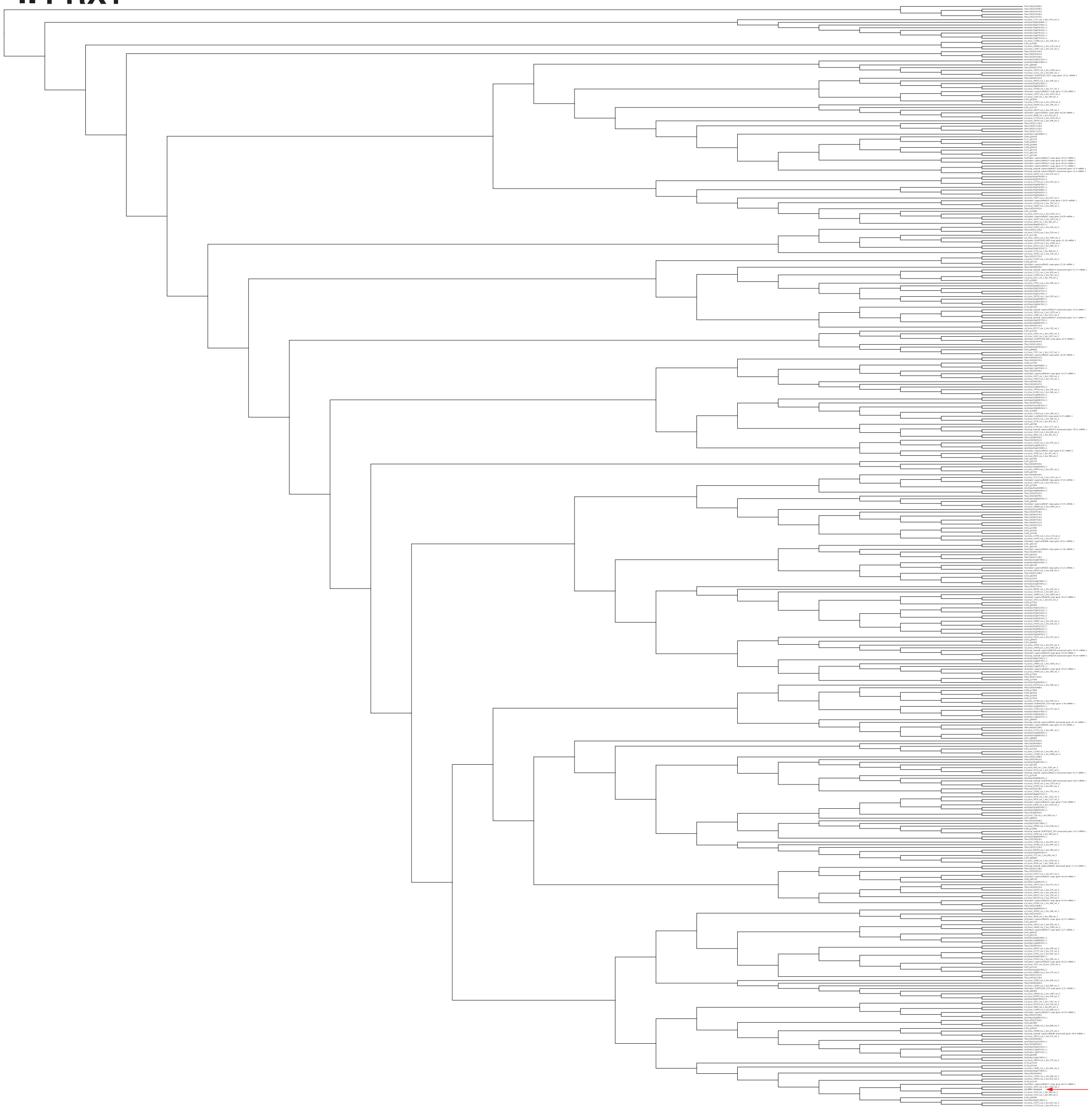

m. RO

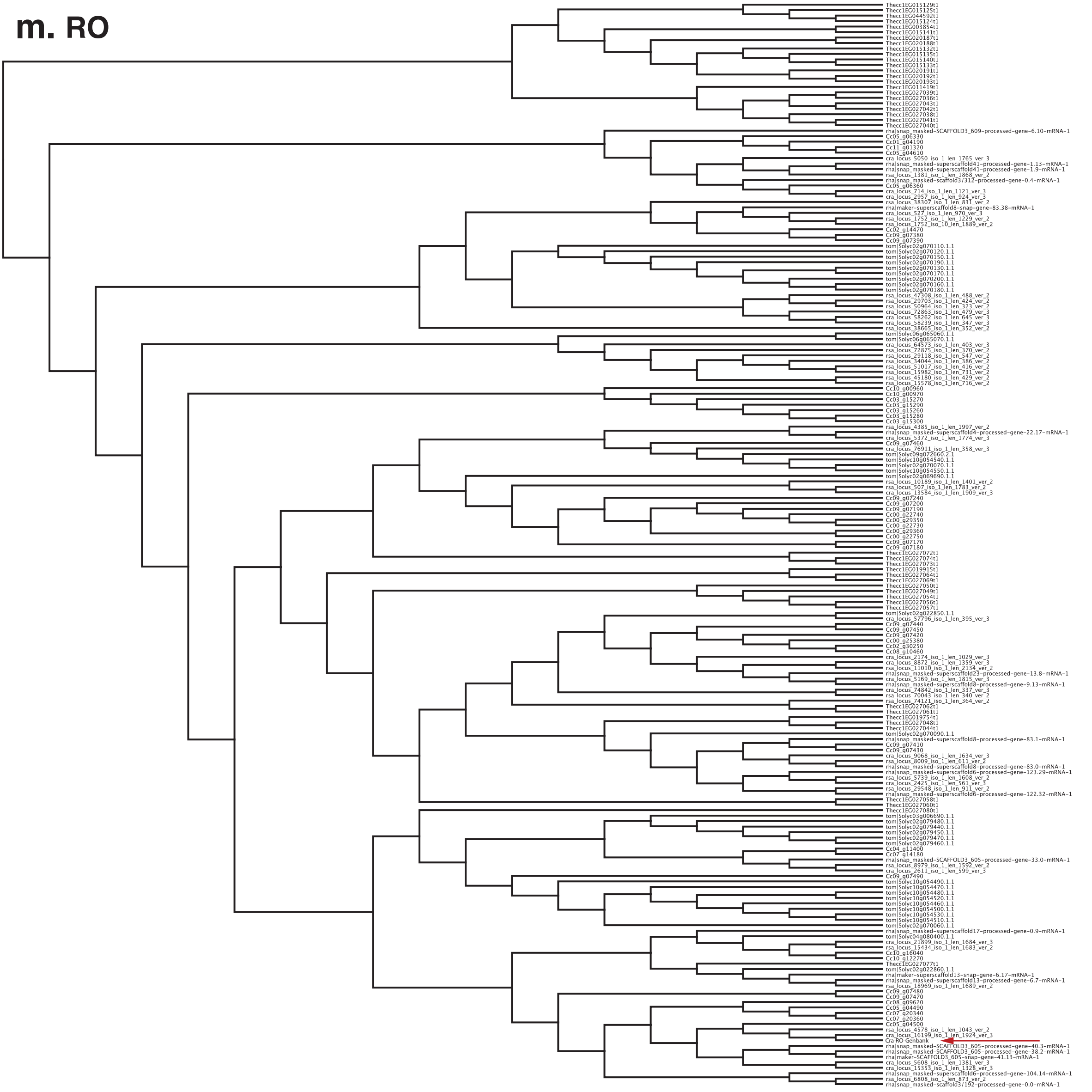
Thecc1EG015129t1
Thecc1EG015125t1
Thecc1EG044592t1
Thecc1EG015124t1
Thecc1EG003854t1
Thecc1EG015141t1
Thecc1EG020187t1
Thecc1EG020188t1
Thecc1EG015132t1
Thecc1EG015135t1
Thecc1EG015140t1
Thecc1EG015133t1
Thecc1EG020191t1
Thecc1EG020192t1
Thecc1EG020193t1
Thecc1EG011419t1
Thecc1EG027039t1
Thecc1EG027036t1
Thecc1EG027043t1
Thecc1EG027042t1
Thecc1EG027038t1
Thecc1EG027041t1
Thecc1EG027040t1
rha|snap_masked-SCAFFOLD3_609-processed-gene-6.10-mRNA-1
Cc05_g06330
Cc01_g04190
Cc11_g01320
Cc05_g04610
cra_locus_5050_iso_1_len_1765_ver_3
rha|snap_masked-superscaffold41-processed-gene-1.13-mRNA-1
rha|snap_masked-superscaffold41-processed-gene-1.9-mRNA-1
rsa_locus_1381_iso_1_len_1868_ver_2
rha|snap_masked-scaffold3/312-processed-gene-0.4-mRNA-1
Cc05_g06360
cra_locus_714_iso_1_len_1121_ver_3
cra_locus_2957_iso_1_len_924_ver_3
rsa_locus_38307_iso_1_len_831_ver_2
rha|maker-superscaffold8-snap-gene-83.38-mRNA-1
cra_locus_527_iso_1_len_970_ver_3
rsa_locus_1752_iso_1_len_1229_ver_2
rsa_locus_1752_iso_10_len_1889_ver_2
Cc02_g14470
Cc09_g07380
Cc09_g07390
tom|Solyc02g070110.1.1
tom|Solyc02g070120.1.1
tom|Solyc02g070150.1.1
tom|Solyc02g070190.1.1
tom|Solyc02g070130.1.1
tom|Solyc02g070170.1.1
tom|Solyc02g070200.1.1
tom|Solyc02g070160.1.1
tom|Solyc02g070180.1.1
rsa_locus_47308_iso_1_len_488_ver_2
rsa_locus_29703_iso_1_len_424_ver_2
rsa_locus_50964_iso_1_len_323_ver_2
cra_locus_72863_iso_1_len_479_ver_3
cra_locus_58262_iso_1_len_645_ver_3
cra_locus_58239_iso_1_len_347_ver_3
rsa_locus_38665_iso_1_len_352_ver_2
tom|Solyc06g065060.1.1
tom|Solyc06g065070.1.1
cra_locus_64573_iso_1_len_403_ver_3
rsa_locus_72875_iso_1_len_370_ver_2
rsa_locus_29118_iso_1_len_547_ver_2
rsa_locus_34044_iso_1_len_386_ver_2
rsa_locus_51017_iso_1_len_416_ver_2
rsa_locus_15982_iso_1_len_731_ver_2
rsa_locus_45180_iso_1_len_429_ver_2
rsa_locus_15578_iso_1_len_716_ver_2
Cc10_g00960
Cc10_g00970
Cc03_g15270
Cc03_g15290
Cc03_g15260
Cc03_g15280
Cc03_g15300
rsa_locus_4385_iso_1_len_1997_ver_2
rha|snap_masked-superscaffold4-processed-gene-22.17-mRNA-1
cra_locus_5372_iso_1_len_1774_ver_3
Cc09_g07460
cra_locus_76911_iso_1_len_358_ver_3
tom|Solyc09g072660.2.1
tom|Solyc10g054540.1.1
tom|Solyc02g070070.1.1
tom|Solyc10g054550.1.1
tom|Solyc02g069690.1.1
rsa_locus_10189_iso_1_len_1401_ver_2
rsa_locus_507_iso_1_len_1783_ver_2
cra_locus_13584_iso_1_len_1909_ver_3
Cc09_g07240
Cc09_g07200
Cc09_g07190
Cc00_g22740
Cc00_g29350
Cc00_g22730
Cc00_g29360
Cc00_g22750
Cc09_g07170
Cc09_g07180
Thecc1EG027072t1
Thecc1EG027074t1
Thecc1EG027073t1
Thecc1EG019915t1
Thecc1EG027064t1
Thecc1EG027069t1
Thecc1EG027050t1
Thecc1EG027049t1
Thecc1EG027054t1
Thecc1EG027056t1
Thecc1EG027057t1
tom|Solyc02g022850.1.1
cra_locus_57796_iso_1_len_395_ver_3
Cc09_g07440
Cc09_g07450
Cc09_g07420
Cc00_g25380
Cc02_g30250
Cc08_g10460
cra_locus_2174_iso_1_len_1029_ver_3
cra_locus_8872_iso_1_len_1359_ver_3
rsa_locus_11010_iso_1_len_2134_ver_2
rha|snap_masked-superscaffold23-processed-gene-13.8-mRNA-1
cra_locus_5169_iso_1_len_1815_ver_3
rha|snap_masked-superscaffold8-processed-gene-9.13-mRNA-1
cra_locus_74842_iso_1_len_337_ver_3
rsa_locus_70043_iso_1_len_340_ver_2
rsa_locus_74121_iso_1_len_364_ver_2
Thecc1EG027062t1
Thecc1EG027061t1
Thecc1EG019754t1
Thecc1EG027048t1
Thecc1EG027044t1
tom|Solyc02g070090.1.1
rha|snap_masked-superscaffold8-processed-gene-83.1-mRNA-1
Cc09_g07410
Cc09_g07430
cra_locus_9068_iso_1_len_1634_ver_3
rsa_locus_8009_iso_1_len_611_ver_2
rha|snap_masked-superscaffold8-processed-gene-83.0-mRNA-1
rha|snap_masked-superscaffold6-processed-gene-123.29-mRNA-1
rsa_locus_5739_iso_1_len_1608_ver_2
cra_locus_2425_iso_1_len_561_ver_3
rsa_locus_29548_iso_1_len_911_ver_2
rha|snap_masked-superscaffold6-processed-gene-122.32-mRNA-1
Thecc1EG027058t1
Thecc1EG027060t1
Thecc1EG027080t1
tom|Solyc03g006690.1.1
tom|Solyc02g079480.1.1
tom|Solyc02g079440.1.1
tom|Solyc02g079450.1.1
tom|Solyc02g079470.1.1
tom|Solyc02g079460.1.1
Cc04_g11400
Cc07_g14180
rha|snap_masked-SCAFFOLD3_605-processed-gene-33.0-mRNA-1
rsa_locus_8979_iso_1_len_1592_ver_2
cra_locus_2611_iso_1_len_599_ver_3
Cc09_g07490
tom|Solyc10g054490.1.1
tom|Solyc10g054470.1.1
tom|Solyc10g054480.1.1
tom|Solyc10g054520.1.1
tom|Solyc10g054460.1.1
tom|Solyc10g054500.1.1
tom|Solyc10g054530.1.1
tom|Solyc10g054510.1.1
tom|Solyc02g070060.1.1
rha|snap_masked-superscaffold17-processed-gene-0.9-mRNA-1
tom|Solyc04g080400.1.1
cra_locus_21899_iso_1_len_1684_ver_3
rsa_locus_15434_iso_1_len_1683_ver_2
Cc10_g16040
Cc10_g12270
Thecc1EG027077t1
tom|Solyc02g022860.1.1
rha|maker-superscaffold13-snap-gene-6.17-mRNA-1
rha|snap_masked-superscaffold13-processed-gene-6.7-mRNA-1
rsa_locus_18969_iso_1_len_1689_ver_2
Cc09_g07480
Cc09_g07470
Cc08_g09620
Cc05_g04490
Cc07_g20340
Cc07_g20360
Cc05_g04500
rsa_locus_4578_iso_1_len_1043_ver_2
cra_locus_16199_iso_1_len_1924_ver_3
Cra-RO-Genbank
rha|snap_masked-SCAFFOLD3_605-processed-gene-40.3-mRNA-1
rha|snap_masked-SCAFFOLD3_605-processed-gene-38.2-mRNA-1
rha|maker-SCAFFOLD3_605-snap-gene-41.13-mRNA-1
cra_locus_5608_iso_1_len_1381_ver_3
cra_locus_15353_iso_1_len_1328_ver_3
rha|snap_masked-superscaffold6-processed-gene-104.14-mRNA-1
rsa_locus_6808_iso_1_len_873_ver_2
rha|snap_masked-scaffold3/192-processed-gene-0.0-mRNA-1

n. SGD

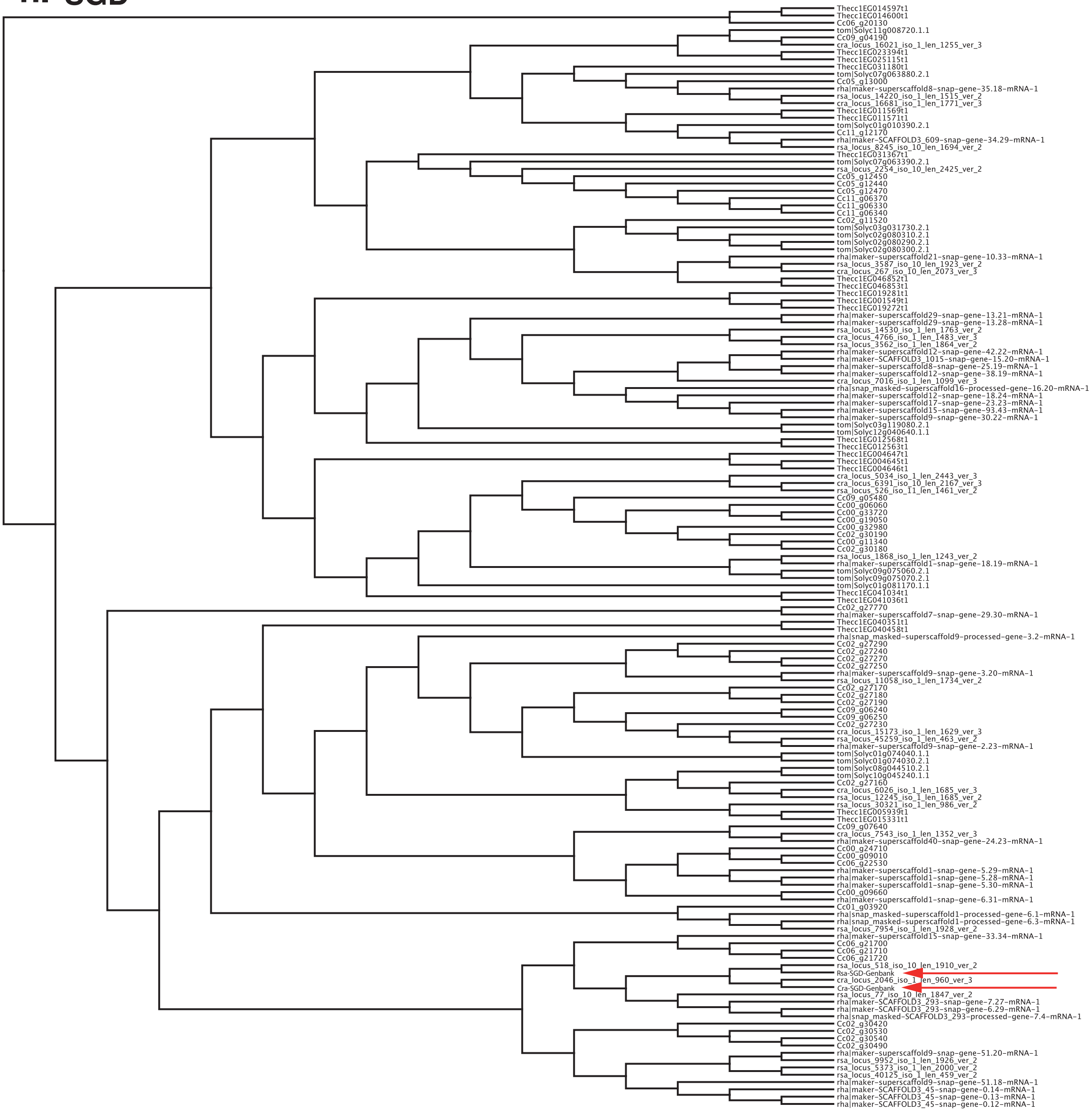
Thecc1EG014597t1
Thecc1EG014600t1
Cc06_g20130
tom|Solyc11g008720.1.1
Cc09_g04190
cra_locus_16021_iso_1_len_1255_ver_3
Thecc1EG023394t1
Thecc1EG025115t1
Thecc1EG031180t1
tom|Solyc07g063880.2.1
Cc05_g13000
rha|maker-superscaffold8-snap-gene-35.18-mRNA-1
rsa_locus_14220_iso_1_len_1515_ver_2
cra_locus_16681_iso_1_len_1771_ver_3
Thecc1EG011569t1
Thecc1EG011571t1
tom|Solyc01g010390.2.1
Cc11_g12170
rha|maker-SCAFFOLD3_609-snap-gene-34.29-mRNA-1
rsa_locus_8245_iso_10_len_1694_ver_2
Thecc1EG031367t1
tom|Solyc07g063390.2.1
rsa_locus_2254_iso_10_len_2425_ver_2
Cc05_g12450
Cc05_g12440
Cc05_g12470
Cc11_g06370
Cc11_g06330
Cc11_g06340
Cc02_g11520
tom|Solyc03g031730.2.1
tom|Solyc02g080310.2.1
tom|Solyc02g080290.2.1
tom|Solyc02g080300.2.1
rha|maker-superscaffold21-snap-gene-10.33-mRNA-1
rsa_locus_3587_iso_10_len_1923_ver_2
cra_locus_267_iso_10_len_2073_ver_3
Thecc1EG046852t1
Thecc1EG046853t1
Thecc1EG019281t1
Thecc1EG001549t1
Thecc1EG019272t1
rha|maker-superscaffold29-snap-gene-13.21-mRNA-1
rha|maker-superscaffold29-snap-gene-13.28-mRNA-1
rsa_locus_14530_iso_1_len_1763_ver_2
cra_locus_4766_iso_1_len_1483_ver_3
rsa_locus_3562_iso_1_len_1864_ver_2
rha|maker-superscaffold12-snap-gene-42.22-mRNA-1
rha|maker-SCAFFOLD3_1015-snap-gene-15.20-mRNA-1
rha|maker-superscaffold8-snap-gene-25.19-mRNA-1
rha|maker-superscaffold12-snap-gene-38.19-mRNA-1
cra_locus_7016_iso_1_len_1099_ver_3
rha|snap_masked-superscaffold16-processed-gene-16.20-mRNA-1
rha|maker-superscaffold12-snap-gene-18.24-mRNA-1
rha|maker-superscaffold17-snap-gene-23.23-mRNA-1
rha|maker-superscaffold15-snap-gene-93.43-mRNA-1
rha|maker-superscaffold9-snap-gene-30.22-mRNA-1
tom|Solyc03g119080.2.1
tom|Solyc12g040640.1.1
Thecc1EG012568t1
Thecc1EG012563t1
Thecc1EG004647t1
Thecc1EG004645t1
Thecc1EG004646t1
cra_locus_5034_iso_1_len_2443_ver_3
cra_locus_6391_iso_10_len_2167_ver_3
rsa_locus_526_iso_11_len_1461_ver_2
Cc09_g05480
Cc00_g06060
Cc00_g33720
Cc00_g19050
Cc00_g32980
Cc02_g30190
Cc00_g11340
Cc02_g30180
rsa_locus_1868_iso_1_len_1243_ver_2
rha|maker-superscaffold1-snap-gene-18.19-mRNA-1
tom|Solyc09g075060.2.1
tom|Solyc09g075070.2.1
tom|Solyc01g081170.1.1
Thecc1EG041034t1
Thecc1EG041036t1
Cc02_g27770
rha|maker-superscaffold7-snap-gene-29.30-mRNA-1
Thecc1EG040351t1
Thecc1EG040458t1
rha|snap_masked-superscaffold9-processed-gene-3.2-mRNA-1
Cc02_g27290
Cc02_g27240
Cc02_g27270
Cc02_g27250
rha|maker-superscaffold9-snap-gene-3.20-mRNA-1
rsa_locus_11058_iso_1_len_1734_ver_2
Cc02_g27170
Cc02_g27180
Cc02_g27190
Cc09_g06240
Cc09_g06250
Cc02_g27230
cra_locus_15173_iso_1_len_1629_ver_3
rsa_locus_45259_iso_1_len_463_ver_2
rha|maker-superscaffold9-snap-gene-2.23-mRNA-1
tom|Solyc01g074040.1.1
tom|Solyc01g074030.2.1
tom|Solyc08g044510.2.1
tom|Solyc10g045240.1.1
Cc02_g27160
cra_locus_6026_iso_1_len_1685_ver_3
rsa_locus_12245_iso_1_len_1685_ver_2
rsa_locus_30321_iso_1_len_986_ver_2
Thecc1EG005939t1
Thecc1EG015331t1
Cc09_g07640
cra_locus_7543_iso_1_len_1352_ver_3
rha|maker-superscaffold40-snap-gene-24.23-mRNA-1
Cc00_g24710
Cc00_g09010
Cc06_g22530
rha|maker-superscaffold1-snap-gene-5.29-mRNA-1
rha|maker-superscaffold1-snap-gene-5.28-mRNA-1
rha|maker-superscaffold1-snap-gene-5.30-mRNA-1
Cc00_g09660
rha|maker-superscaffold1-snap-gene-6.31-mRNA-1
Cc01_g03920
rha|snap_masked-superscaffold1-processed-gene-6.1-mRNA-1
rha|snap_masked-superscaffold1-processed-gene-6.3-mRNA-1
rsa_locus_7954_iso_1_len_1928_ver_2
rha|maker-superscaffold15-snap-gene-33.34-mRNA-1
Cc06_g21700
Cc06_g21710
Cc06_g21720
rsa_locus_518_iso_10_len_1910_ver_2
Rsa-SGD-Genbank
cra_locus_2046_iso_1_len_960_ver_3
Cra-SGD-Genbank
rsa_locus_77_iso_10_len_1847_ver_2
rha|maker-SCAFFOLD3_293-snap-gene-7.27-mRNA-1
rha|maker-SCAFFOLD3_293-snap-gene-6.29-mRNA-1
rha|snap_masked-SCAFFOLD3_293-processed-gene-7.4-mRNA-1
Cc02_g30420
Cc02_g30530
Cc02_g30540
Cc02_g30490
rha|maker-superscaffold9-snap-gene-51.20-mRNA-1
rsa_locus_9952_iso_1_len_1926_ver_2
rsa_locus_5373_iso_1_len_2000_ver_2
rsa_locus_40125_iso_1_len_459_ver_2
rha|maker-superscaffold9-snap-gene-51.18-mRNA-1
rha|maker-SCAFFOLD3_45-snap-gene-0.14-mRNA-1
rha|maker-SCAFFOLD3_45-snap-gene-0.13-mRNA-1
rha|maker-SCAFFOLD3_45-snap-gene-0.12-mRNA-1

o. SLS

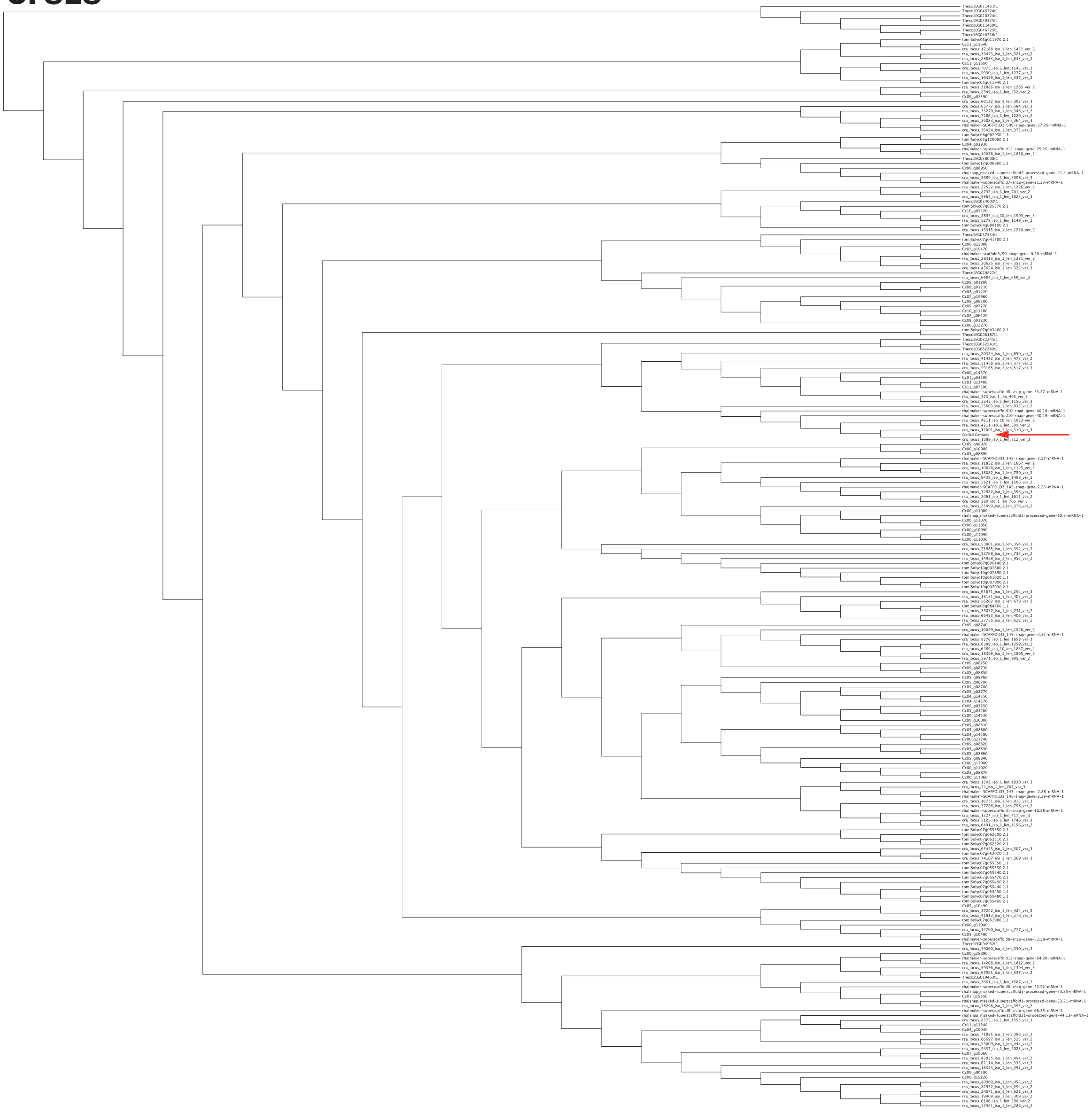

p. STR

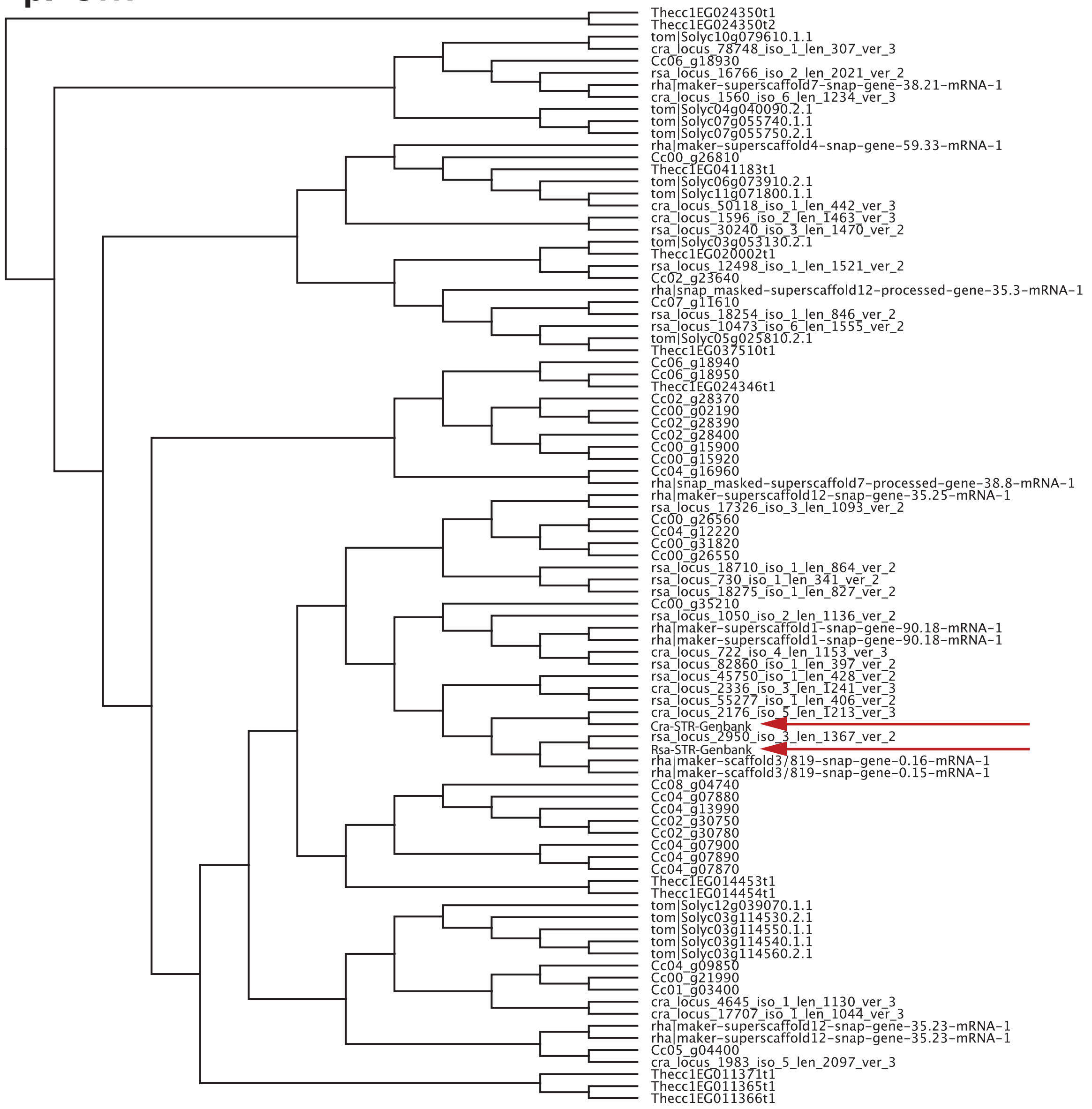
Thecc1EG024350t1
Thecc1EG024350t2
tom|Solyc10g079610.1.1
cra_locus_78748_iso_1_len_307_ver_3
Cc06_g18930
rsa_locus_16766_iso_2_len_2021_ver_2
rha|maker-superscaffold7-snap-gene-38.21-mRNA-1
cra_locus_1560_iso_6_len_1234_ver_3
tom|Solyc04g040090.2.1
tom|Solyc07g055740.1.1
tom|Solyc07g055750.2.1
rha|maker-superscaffold4-snap-gene-59.33-mRNA-1
Cc00_g26810
Thecc1EG041183t1
tom|Solyc06g073910.2.1
tom|Solyc11g071800.1.1
cra_locus_50118_iso_1_len_442_ver_3
cra_locus_1596_iso_2_len_1463_ver_3
rsa_locus_30240_iso_3_len_1470_ver_2
tom|Solyc03g053130.2.1
Thecc1EG020002t1
rsa_locus_12498_iso_1_len_1521_ver_2
Cc02_g23640
rha|snap_masked-superscaffold12-processed-gene-35.3-mRNA-1
Cc07_g11610
rsa_locus_18254_iso_1_len_846_ver_2
rsa_locus_10473_iso_6_len_1555_ver_2
tom|Solyc05g025810.2.1
Thecc1EG037510t1
Cc06_g18940
Cc06_g18950
Thecc1EG024346t1
Cc02_g28370
Cc00_g02190
Cc02_g28390
Cc02_g28400
Cc00_g15900
Cc00_g15920
Cc04_g16960
rha|snap_masked-superscaffold7-processed-gene-38.8-mRNA-1
rha|maker-superscaffold12-snap-gene-35.25-mRNA-1
rsa_locus_17326_iso_3_len_1093_ver_2
Cc00_g26560
Cc04_g12220
Cc00_g31820
Cc00_g26550
rsa_locus_18710_iso_1_len_864_ver_2
rsa_locus_730_iso_1_len_341_ver_2
rsa_locus_18275_iso_1_len_827_ver_2
Cc00_g35210
rsa_locus_1050_iso_2_len_1136_ver_2
rha|maker-superscaffold1-snap-gene-90.18-mRNA-1
rha|maker-superscaffold1-snap-gene-90.18-mRNA-1
cra_locus_722_iso_4_len_1153_ver_3
rsa_locus_82860_iso_1_len_397_ver_2
rsa_locus_45750_iso_1_len_428_ver_2
cra_locus_2336_iso_3_len_1241_ver_3
rsa_locus_55277_iso_1_len_406_ver_2
cra_locus_2176_iso_5_len_1213_ver_3
Cra-STR-Genbank
rsa_locus_2950_iso_3_len_1367_ver_2
Rsa-STR-Genbank
rha|maker-scaffold3/819-snap-gene-0.16-mRNA-1
rha|maker-scaffold3/819-snap-gene-0.15-mRNA-1
Cc08_g04740
Cc04_g07880
Cc04_g13990
Cc02_g30750
Cc02_g30780
Cc04_g07900
Cc04_g07890
Cc04_g07870
Thecc1EG014453t1
Thecc1EG014454t1
tom|Solyc12g039070.1.1
tom|Solyc03g114530.2.1
tom|Solyc03g114550.1.1
tom|Solyc03g114540.1.1
tom|Solyc03g114560.2.1
Cc04_g09850
Cc00_g21990
Cc01_g03400
cra_locus_4645_iso_1_len_1130_ver_3
cra_locus_17707_iso_1_len_1044_ver_3
rha|maker-superscaffold12-snap-gene-35.23-mRNA-1
rha|maker-superscaffold12-snap-gene-35.23-mRNA-1
Cc05_g04400
cra_locus_1983_iso_5_len_2097_ver_3
Thecc1EG011371t1
Thecc1EG011365t1
Thecc1EG011366t1

q. T16H

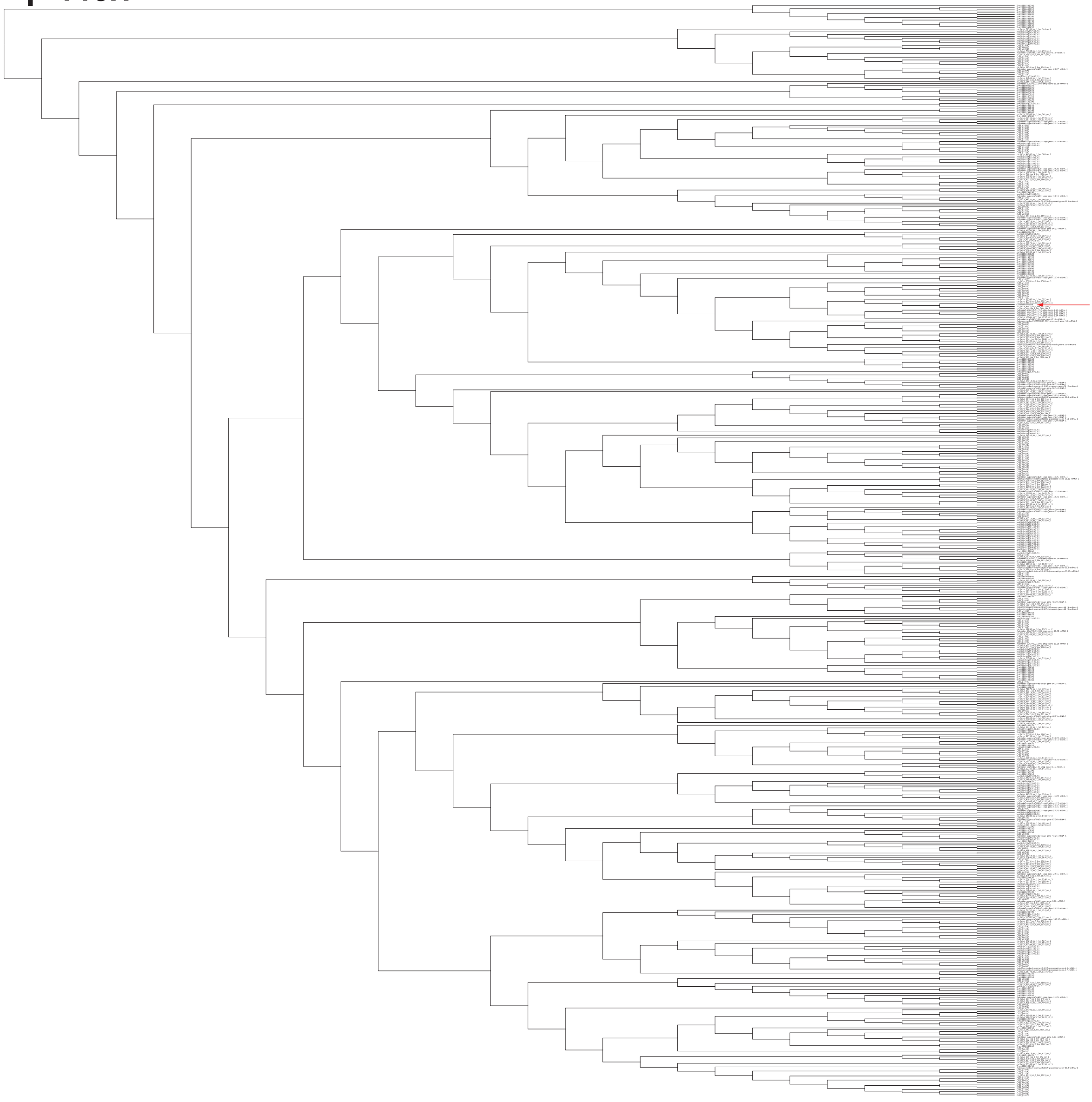

r. T19H

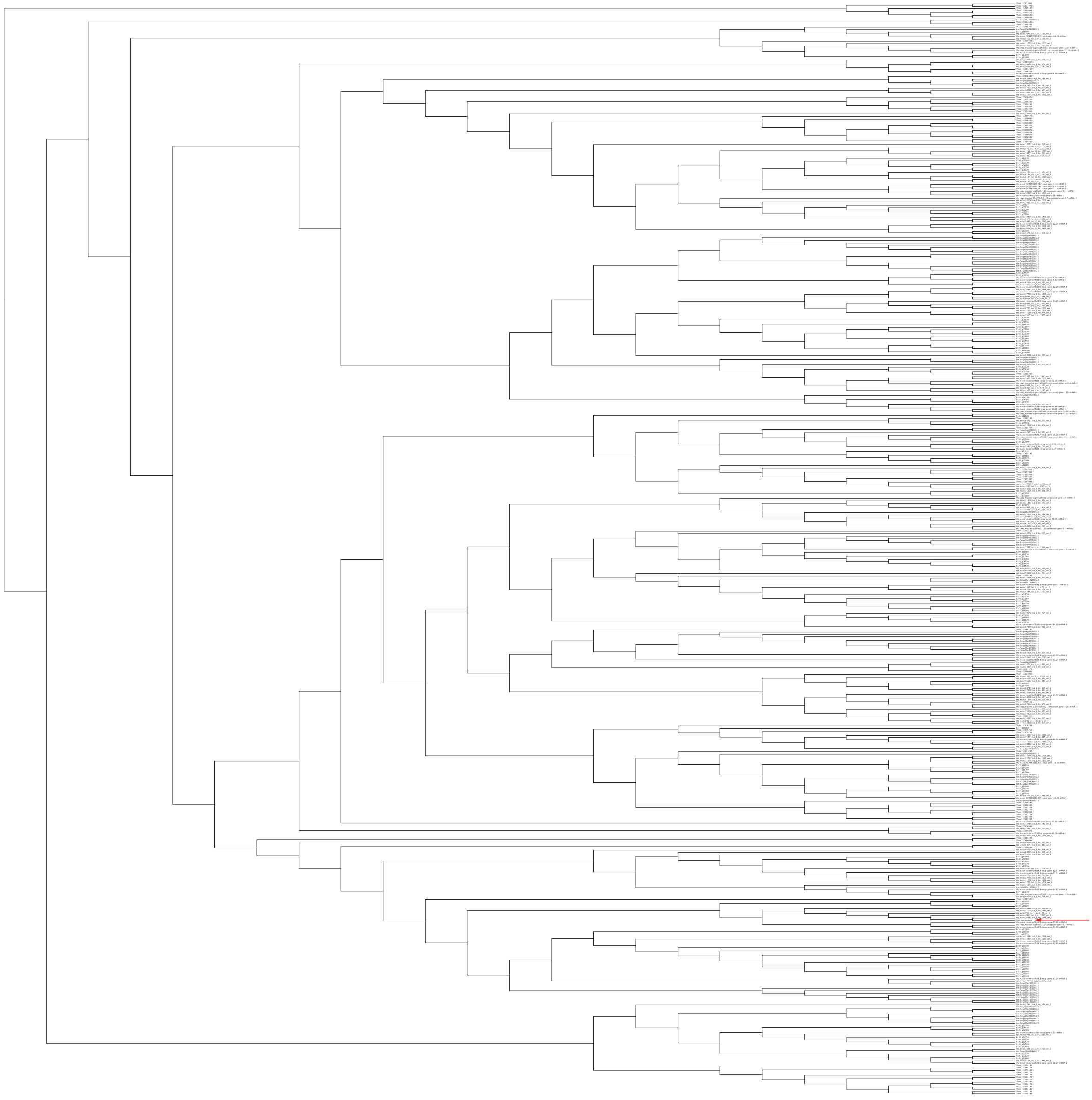

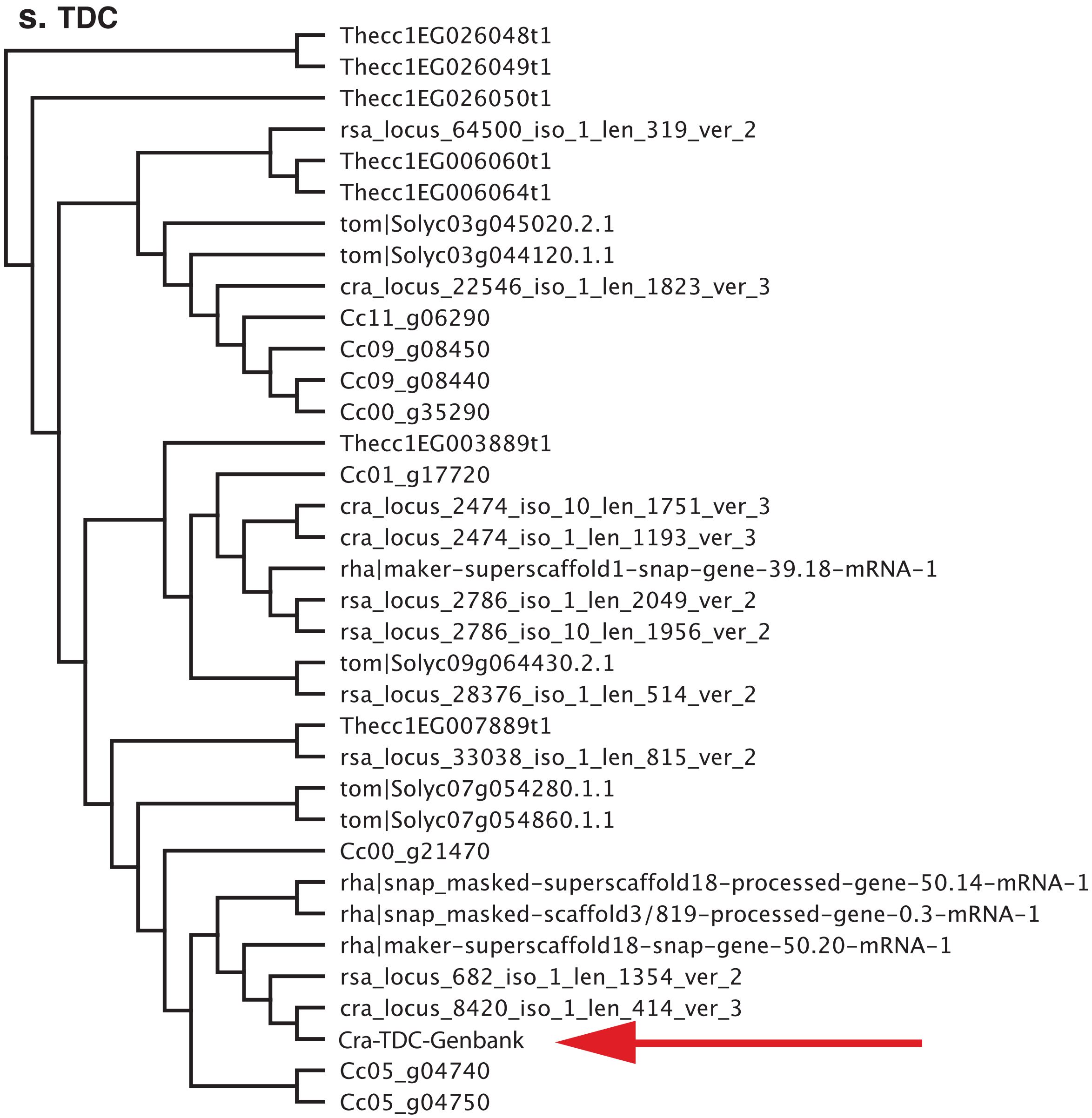
s. TDC
Thecc1EG026048t1
Thecc1EG026049t1
Thecc1EG026050t1
rsa_locus_64500_iso_1_len_319_ver_2
Thecc1EG006060t1
Thecc1EG006064t1
tom|Solyc03g045020.2.1
tom|Solyc03g044120.1.1
cra_locus_22546_iso_1_len_1823_ver_3
Cc11_g06290
Cc09_g08450
Cc09_g08440
Cc00_g35290
Thecc1EG003889t1
Cc01_g17720
cra_locus_2474_iso_10_len_1751_ver_3
cra_locus_2474_iso_1_len_1193_ver_3
rha|maker-superscaffold1-snap-gene-39.18-mRNA-1
rsa_locus_2786_iso_1_len_2049_ver_2
rsa_locus_2786_iso_10_len_1956_ver_2
tom|Solyc09g064430.2.1
rsa_locus_28376_iso_1_len_514_ver_2
Thecc1EG007889t1
rsa_locus_33038_iso_1_len_815_ver_2
tom|Solyc07g054280.1.1
tom|Solyc07g054860.1.1
Cc00_g21470
rha|snap_masked-superscaffold18-processed-gene-50.14-mRNA-1
rha|snap_masked-scaffold3/819-processed-gene-0.3-mRNA-1
rha|maker-superscaffold18-snap-gene-50.20-mRNA-1
rsa_locus_682_iso_1_len_1354_ver_2
cra_locus_8420_iso_1_len_414_ver_3
Cra-TDC-Genbank
Cc05_g04740
Cc05_g04750

t. THAS

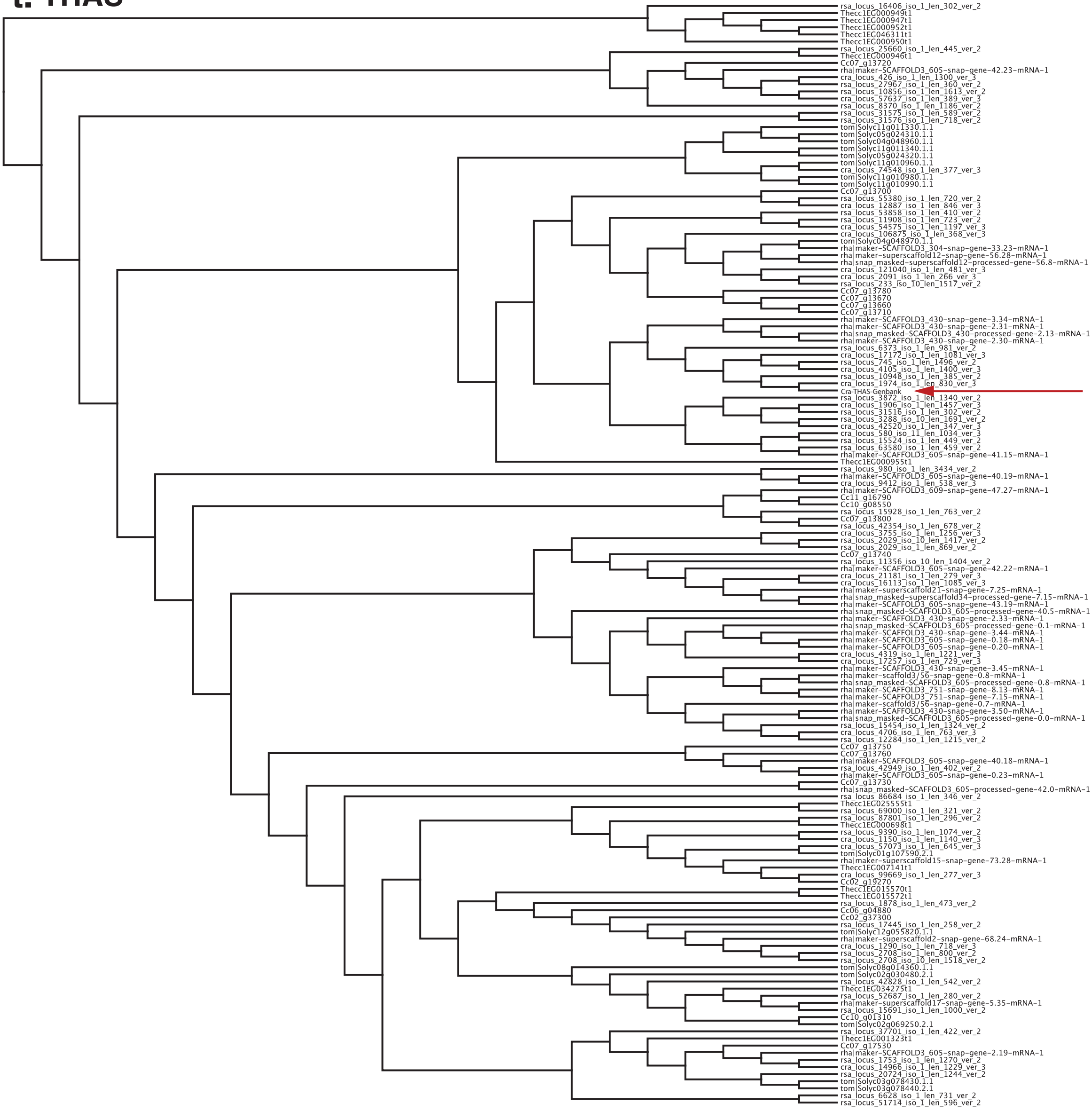
rsa_locus_16406_iso_1_len_302_ver_2
Thecc1EG000949t1
Thecc1EG000947t1
Thecc1EG000952t1
Thecc1EG046311t1
Thecc1EG000950t1
rsa_locus_25660_iso_1_len_445_ver_2
Thecc1EG000946t1
Cc07_g13720
rha|maker-SCAFFOLD3_605-snap-gene-42.23-mRNA-1
cra_locus_426_iso_1_len_1300_ver_3
rsa_locus_27967_iso_1_len_360_ver_2
rsa_locus_10856_iso_1_len_1613_ver_2
cra_locus_57637_iso_1_len_389_ver_3
rsa_locus_8370_iso_1_len_1186_ver_2
rsa_locus_31575_iso_1_len_589_ver_2
rsa_locus_31576_iso_1_len_718_ver_2
tom|Solyc11g011330.1.1
tom|Solyc05g024310.1.1
tom|Solyc04g048960.1.1
tom|Solyc11g011340.1.1
tom|Solyc05g024320.1.1
tom|Solyc11g010960.1.1
cra_locus_74548_iso_1_len_377_ver_3
tom|Solyc11g010980.1.1
tom|Solyc11g010990.1.1
Cc07_g13700
rsa_locus_55380_iso_1_len_720_ver_2
cra_locus_12887_iso_1_len_846_ver_3
rsa_locus_53858_iso_1_len_410_ver_2
rsa_locus_11908_iso_1_len_723_ver_2
cra_locus_54575_iso_1_len_1197_ver_3
cra_locus_106875_iso_1_len_368_ver_3
tom|Solyc04g048970.1.1
rha|maker-SCAFFOLD3_304-snap-gene-33.23-mRNA-1
rha|maker-superscaffold12-snap-gene-56.28-mRNA-1
rha|snap_masked-superscaffold12-processed-gene-56.8-mRNA-1
cra_locus_121040_iso_1_len_481_ver_3
cra_locus_2091_iso_1_len_266_ver_3
rsa_locus_233_iso_10_len_1517_ver_2
Cc07_g13780
Cc07_g13670
Cc07_g13660
Cc07_g13710
rha|maker-SCAFFOLD3_430-snap-gene-3.34-mRNA-1
rha|maker-SCAFFOLD3_430-snap-gene-2.31-mRNA-1
rha|snap_masked-SCAFFOLD3_430-processed-gene-2.13-mRNA-1
rha|maker-SCAFFOLD3_430-snap-gene-2.30-mRNA-1
rsa_locus_6373_iso_1_len_981_ver_2
cra_locus_17172_iso_1_len_1081_ver_3
rsa_locus_745_iso_1_len_1496_ver_2
cra_locus_4105_iso_1_len_1400_ver_3
rsa_locus_10948_iso_1_len_385_ver_2
cra_locus_1974_iso_1_len_830_ver_3
Cra-THAS-Genbank
rsa_locus_3872_iso_1_len_1340_ver_2
cra_locus_1906_iso_1_len_1457_ver_3
rsa_locus_31516_iso_1_len_302_ver_2
rsa_locus_3288_iso_10_len_1691_ver_2
cra_locus_42520_iso_1_len_347_ver_3
cra_locus_580_iso_11_len_1034_ver_3
rsa_locus_15524_iso_1_len_449_ver_2
rsa_locus_63580_iso_1_len_459_ver_2
rha|maker-SCAFFOLD3_605-snap-gene-41.15-mRNA-1
Thecc1EG000955t1
rsa_locus_980_iso_1_len_3434_ver_2
rha|maker-SCAFFOLD3_605-snap-gene-40.19-mRNA-1
cra_locus_9412_iso_1_len_538_ver_3
rha|maker-SCAFFOLD3_609-snap-gene-47.27-mRNA-1
Cc11_g16790
Cc10_g08550
rsa_locus_15928_iso_1_len_763_ver_2
Cc07_g13800
rsa_locus_42354_iso_1_len_678_ver_2
cra_locus_3755_iso_1_len_1256_ver_3
rsa_locus_2029_iso_10_len_1417_ver_2
rsa_locus_2029_iso_1_len_869_ver_2
Cc07_g13740
rsa_locus_11356_iso_10_len_1404_ver_2
rha|maker-SCAFFOLD3_605-snap-gene-42.22-mRNA-1
cra_locus_21181_iso_1_len_279_ver_3
cra_locus_16113_iso_1_len_1085_ver_3
rha|maker-superscaffold21-snap-gene-7.25-mRNA-1
rha|snap_masked-superscaffold34-processed-gene-7.15-mRNA-1
rha|maker-SCAFFOLD3_605-snap-gene-43.19-mRNA-1
rha|snap_masked-SCAFFOLD3_605-processed-gene-40.5-mRNA-1
rha|maker-SCAFFOLD3_430-snap-gene-2.33-mRNA-1
rha|snap_masked-SCAFFOLD3_605-processed-gene-0.1-mRNA-1
rha|maker-SCAFFOLD3_430-snap-gene-3.44-mRNA-1
rha|maker-SCAFFOLD3_605-snap-gene-0.18-mRNA-1
rha|maker-SCAFFOLD3_605-snap-gene-0.20-mRNA-1
cra_locus_4319_iso_1_len_1221_ver_3
cra_locus_17257_iso_1_len_729_ver_3
rha|maker-SCAFFOLD3_430-snap-gene-3.45-mRNA-1
rha|maker-scaffold3/56-snap-gene-0.8-mRNA-1
rha|snap_masked-SCAFFOLD3_605-processed-gene-0.8-mRNA-1
rha|maker-SCAFFOLD3_751-snap-gene-8.13-mRNA-1
rha|maker-SCAFFOLD3_751-snap-gene-7.15-mRNA-1
rha|maker-scaffold3/56-snap-gene-0.7-mRNA-1
rha|maker-SCAFFOLD3_430-snap-gene-3.50-mRNA-1
rha|snap_masked-SCAFFOLD3_605-processed-gene-0.0-mRNA-1
rsa_locus_15454_iso_1_len_1324_ver_2
cra_locus_4706_iso_1_len_763_ver_3
rsa_locus_12284_iso_1_len_1215_ver_2
Cc07_g13750
Cc07_g13760
rha|maker-SCAFFOLD3_605-snap-gene-40.18-mRNA-1
rsa_locus_42949_iso_1_len_402_ver_2
rha|maker-SCAFFOLD3_605-snap-gene-0.23-mRNA-1
Cc07_g13730
rha|snap_masked-SCAFFOLD3_605-processed-gene-42.0-mRNA-1
rsa_locus_86684_iso_1_len_346_ver_2
Thecc1EG025555t1
rsa_locus_69000_iso_1_len_321_ver_2
rsa_locus_87801_iso_1_len_296_ver_2
Thecc1EG000698t1
rsa_locus_9390_iso_1_len_1074_ver_2
cra_locus_1150_iso_1_len_1140_ver_3
cra_locus_57073_iso_1_len_645_ver_3
tom|Solyc01g107590.2.1
rha|maker-superscaffold15-snap-gene-73.28-mRNA-1
Thecc1EG007141t1
cra_locus_99669_iso_1_len_277_ver_3
Cc02_g19270
Thecc1EG015570t1
Thecc1EG015572t1
rsa_locus_1878_iso_1_len_473_ver_2
Cc06_g04880
Cc02_g37300
rsa_locus_17445_iso_1_len_258_ver_2
tom|Solyc12g055820.1.1
rha|maker-superscaffold2-snap-gene-68.24-mRNA-1
cra_locus_1290_iso_1_len_718_ver_3
rsa_locus_2708_iso_1_len_800_ver_2
rsa_locus_2708_iso_10_len_1518_ver_2
tom|Solyc08g014360.1.1
tom|Solyc02g030480.2.1
rsa_locus_42828_iso_1_len_542_ver_2
Thecc1EG034275t1
rsa_locus_52687_iso_1_len_280_ver_2
rha|maker-superscaffold17-snap-gene-5.35-mRNA-1
rsa_locus_15691_iso_1_len_1000_ver_2
Cc10_g01310
tom|Solyc02g069250.2.1
rsa_locus_37701_iso_1_len_422_ver_2
Thecc1EG001323t1
Cc07_g17530
rha|maker-SCAFFOLD3_605-snap-gene-2.19-mRNA-1
rsa_locus_1753_iso_1_len_1270_ver_2
cra_locus_14966_iso_1_len_1229_ver_3
rsa_locus_20724_iso_1_len_1244_ver_2
tom|Solyc03g078430.1.1
tom|Solyc03g078440.2.1
rsa_locus_6628_iso_1_len_731_ver_2
rsa_locus_51714_iso_1_len_596_ver_2

u. VS

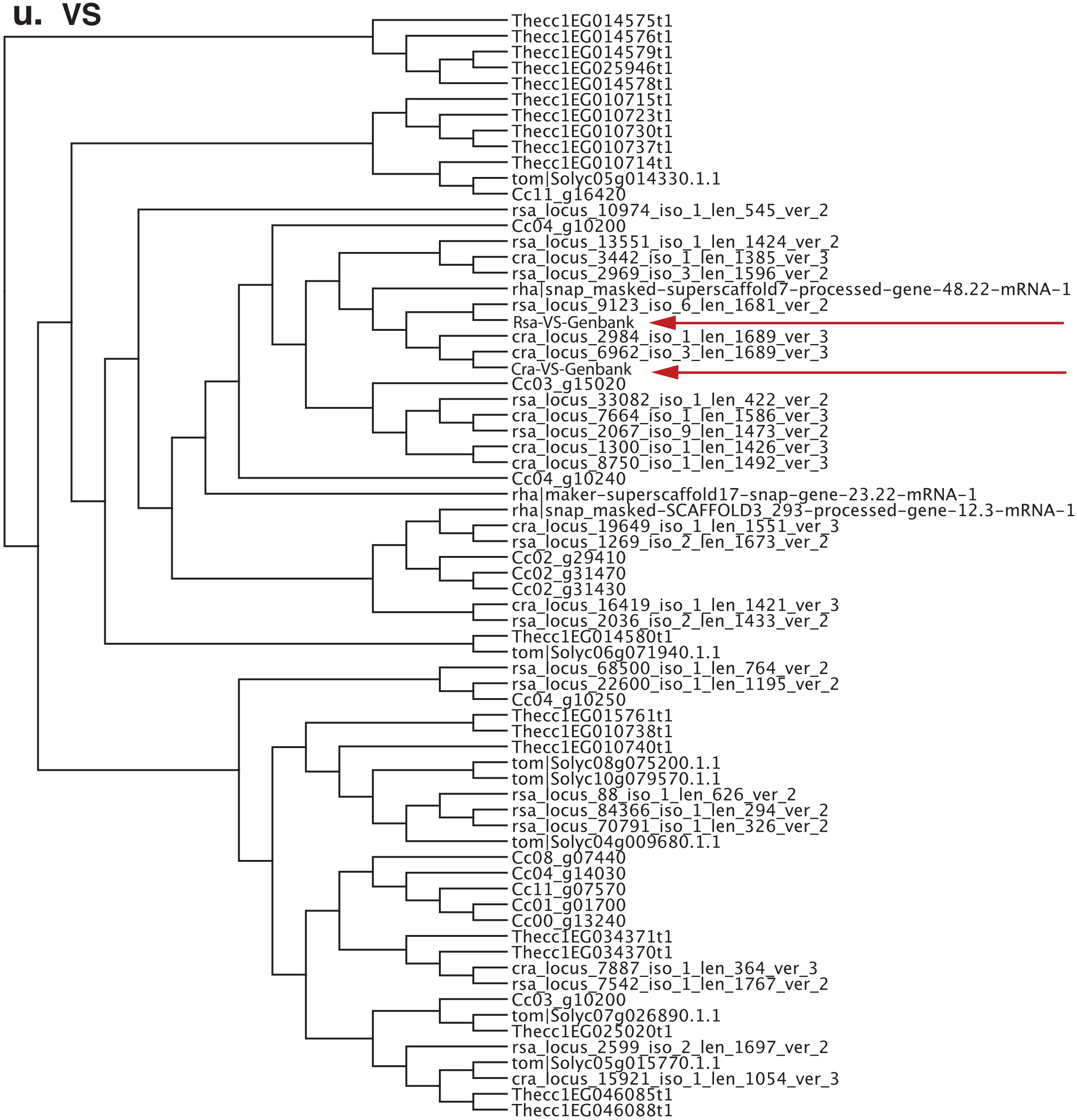

Thecc1EG014575t1
Thecc1EG014576t1
Thecc1EG014579t1
Thecc1EG025946t1
Thecc1EG014578t1
Thecc1EG010715t1
Thecc1EG010723t1
Thecc1EG010730t1
Thecc1EG010737t1
Thecc1EG010714t1
tom|Solyc05g014330.1.1
Cc11_g16420
rsa_locus_10974_iso_1_len_545_ver_2
Cc04_g10200
rsa_locus_13551_iso_1_len_1424_ver_2
cra_locus_3442_iso_1_len_1385_ver_3
rsa_locus_2969_iso_3_len_1596_ver_2
rha|snap_masked-superscaffold7-processed-gene-48.22-mRNA-1
rsa_locus_9123_iso_6_len_1681_ver_2
Rsa-VS-Genbank
cra_locus_2984_iso_1_len_1689_ver_3
cra_locus_6962_iso_3_len_1689_ver_3
Cra-VS-Genbank
Cc03_g15020
rsa_locus_33082_iso_1_len_422_ver_2
cra_locus_7664_iso_1_len_1586_ver_3
rsa_locus_2067_iso_9_len_1473_ver_2
cra_locus_1300_iso_1_len_1426_ver_3
cra_locus_8750_iso_1_len_1492_ver_3
Cc04_g10240
rha|maker-superscaffold17-snap-gene-23.22-mRNA-1
rha|snap_masked-SCAFFOLD3_293-processed-gene-12.3-mRNA-1
cra_locus_19649_iso_1_len_1551_ver_3
rsa_locus_1269_iso_2_len_1673_ver_2
Cc02_g29410
Cc02_g31470
Cc02_g31430
cra_locus_16419_iso_1_len_1421_ver_3
rsa_locus_2036_iso_2_len_1433_ver_2
Thecc1EG014580t1
tom|Solyc06g071940.1.1
rsa_locus_68500_iso_1_len_764_ver_2
rsa_locus_22600_iso_1_len_1195_ver_2
Cc04_g10250
Thecc1EG015761t1
Thecc1EG010738t1
Thecc1EG010740t1
tom|Solyc08g075200.1.1
tom|Solyc10g079570.1.1
rsa_locus_88_iso_1_len_626_ver_2
rsa_locus_84366_iso_1_len_294_ver_2
rsa_locus_70791_iso_1_len_326_ver_2
tom|Solyc04g009680.1.1
Cc08_g07440
Cc04_g14030
Cc11_g07570
Cc01_g01700
Cc00_g13240
Thecc1EG034371t1
Thecc1EG034370t1
cra_locus_7887_iso_1_len_364_ver_3
rsa_locus_7542_iso_1_len_1767_ver_2
Cc03_g10200
tom|Solyc07g026890.1.1
Thecc1EG025020t1
rsa_locus_2599_iso_2_len_1697_ver_2
tom|Solyc05g015770.1.1
cra_locus_15921_iso_1_len_1054_ver_3
Thecc1EG046085t1
Thecc1EG046088t1

a. AAE

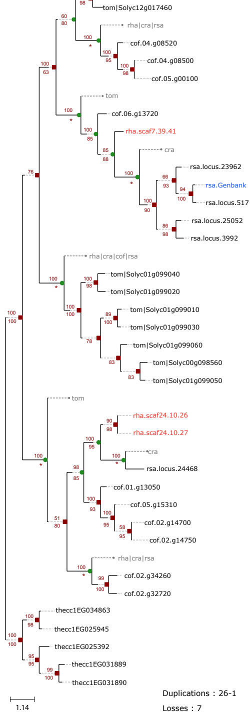

tom|Solyc12g096620
tom|Solyc12g017460
rha|cra|rsa
cof.04.g08520
cof.04.g08500
cof.05.g00100
tom
cof.06.g13720
rha.scaf7.39.41
cra
rsa.locus.23962
rsa.Genbank
rsa.locus.517
rsa.locus.25052
rsa.locus.3992
rha|cra|cof|rsa
tom|Solyc01g099040
tom|Solyc01g099020
tom|Solyc01g099010
tom|Solyc01g099030
tom|Solyc01g099060
tom|Solyc00g098560
tom|Solyc01g099050
tom
rha.scaf24.10.26
rha.scaf24.10.27
cra
rsa.locus.24468
cof.01.g13050
cof.05.g15310
cof.02.g14700
cof.02.g14750
rha|cra|rsa
cof.02.g34260
cof.02.g32720
thecc1EG034863
thecc1EG025945
thecc1EG025392
thecc1EG031889
thecc1EG031890
1.14
Duplications : 26-1
Losses : 7

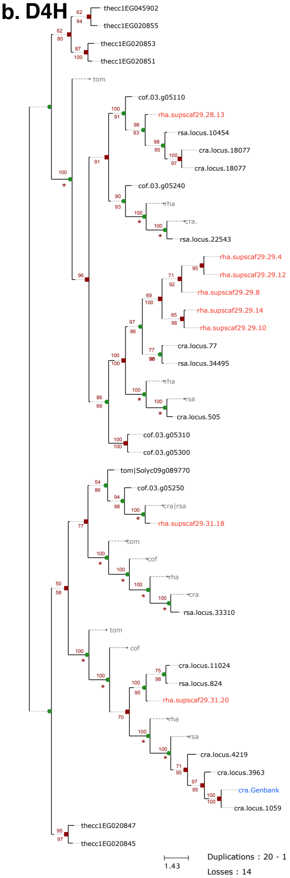
b. D4H
thecc1EG045902
thecc1EG020855
thecc1EG020853
thecc1EG020851
tom
cof.03.g05110
rha.supscaf29.28.13
rsa.locus.10454
cra.locus.18077
cra.locus.18077
cof.03.g05240
rha
cra.
rsa.locus.22543
rha.supscaf29.29.4
rha.supscaf29.29.12
rha.supscaf29.29.8
rha.supscaf29.29.14
rha.supscaf29.29.10
cra.locus.77
rsa.locus.34495
rha
rsa
cra.locus.505
cof.03.g05310
cof.03.g05300
tom|Solyc09g089770
cof.03.g05250
cra|rsa
rha.supscaf29.31.18
tom
cof
rha
cra
rsa.locus.33310
tom
cof
cra.locus.11024
rsa.locus.824
rha.supscaf29.31.20
rha
rsa
cra.locus.4219
cra.locus.3963
cra.Genbank
cra.locus.1059
thecc1EG020847
thecc1EG020845
1.43
Duplications : 20 - 1
Losses : 14

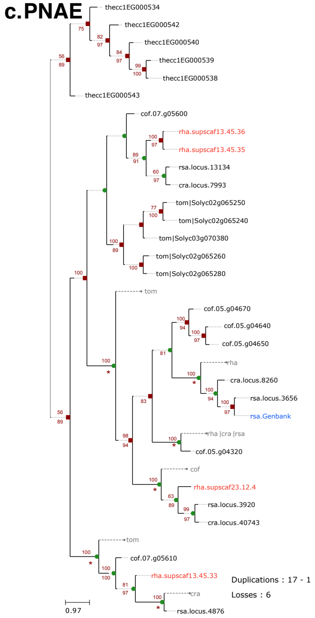
c.PNAE
thecc1EG000534
thecc1EG000542
thecc1EG000540
thecc1EG000539
thecc1EG000538
thecc1EG000543
cof.07.g05600
rha.supscaf13.45.36
rha.supscaf13.45.35
rsa.locus.13134
cra.locus.7993
tom|Solyc02g065250
tom|Solyc02g065240
tom|Solyc03g070380
tom|Solyc02g065260
tom|Solyc02g065280
tom
cof.05.g04670
cof.05.g04640
cof.05.g04650
rha
cra.locus.8260
rsa.locus.3656
rsa.Genbank
rha|cra|rsa
cof.05.g04320
cof
rha.supscaf23.12.4
rsa.locus.3920
cra.locus.40743
tom
cof.07.g05610
rha.supscaf13.45.33
cra
rsa.locus.4876
Duplications : 17 - 1
Losses : 6
0.97

d.SGD

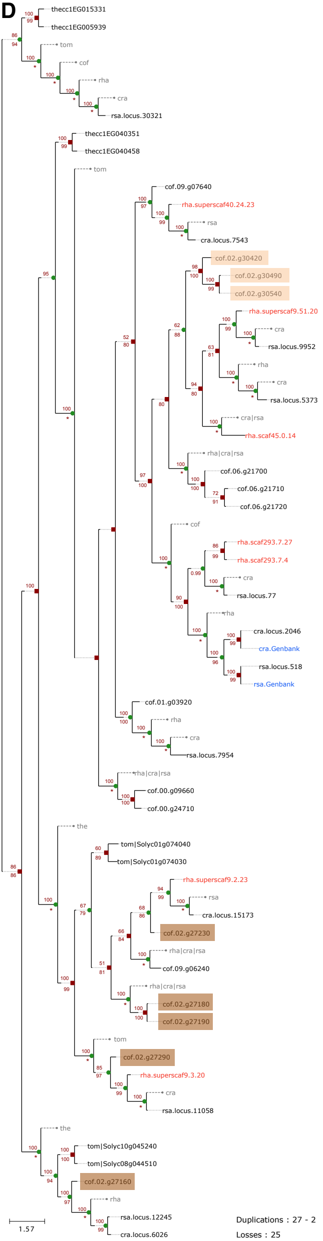
thecc1EG015331
thecc1EG005939
tom
cof
rha
cra
rsa.locus.30321
thecc1EG040351
thecc1EG040458
tom
cof.09.g07640
rha.superscaf40.24.23
rsa
cra.locus.7543
cof.02.g30420
cof.02.g30490
cof.02.g30540
rha.superscaf9.51.20
cra
rsa.locus.9952
rha
cra
rsa.locus.5373
cra|rsa
rha.scaf45.0.14
rha|cra|rsa
cof.06.g21700
cof.06.g21710
cof.06.g21720
cof
rha.scaf293.7.27
rha.scaf293.7.4
cra
rsa.locus.77
rha
cra.locus.2046
cra.Genbank
rsa.locus.518
rsa.Genbank
cof.01.g03920
rha
cra
rsa.locus.7954
rha|cra|rsa
cof.00.g09660
cof.00.g24710
the
tom|Solyc01g074040
tom|Solyc01g074030
rha.superscaf9.2.23
rsa
cra.locus.15173
cof.02.g27230
rha|cra|rsa
cof.09.g06240
rha|cra|rsa
cof.02.g27180
cof.02.g27190
tom
cof.02.g27290
rha.superscaf9.3.20
cra
rsa.locus.11058
the
tom|Solyc10g045240
tom|Solyc08g044510
cof.02.g27160
rha
rsa.locus.12245
cra.locus.6026
1.57
Duplications : 27 - 2
Losses : 25

e. STR

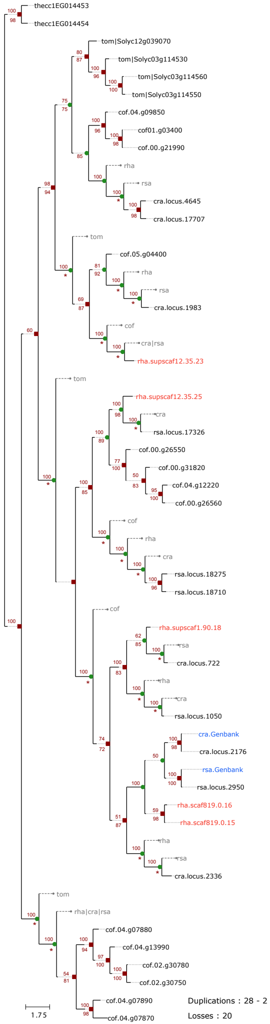

thecc1EG014453
thecc1EG014454
tom|Solyc12g039070
tom|Solyc03g114530
tom|Solyc03g114560
tom|Solyc03g114550
cof.04.g09850
cof01.g03400
cof.00.g21990
rha
rsa
cra.locus.4645
cra.locus.17707
tom
cof.05.g04400
rha
rsa
cra.locus.1983
cof
cra|rsa
rha.supscaf12.35.23
tom
rha.supscaf12.35.25
cra
rsa.locus.17326
cof.00.g26550
cof.00.g31820
cof.04.g12220
cof.00.g26560
cof
rha
cra
rsa.locus.18275
rsa.locus.18710
cof
rha.supscaf1.90.18
rsa
cra.locus.722
rha
cra
rsa.locus.1050
cra.Genbank
cra.locus.2176
rsa.Genbank
rsa.locus.2950
rha.scaf819.0.16
rha.scaf819.0.15
rha
rsa
cra.locus.2336
tom
rha|cra|rsa
cof.04.g07880
cof.04.g13990
cof.02.g30780
cof.02.g30750
cof.04.g07890
cof.04.g07870
1.75
Duplications : 28 - 2
Losses : 20

f. T16H

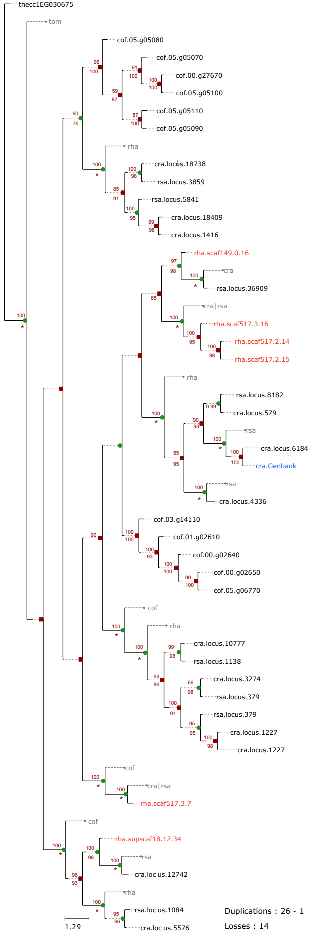
thecc1EG030675
tom
cof.05.g05080
cof.05.g05070
cof.00.g27670
cof.05.g05100
cof.05.g05110
cof.05.g05090
rha
cra.locus.18738
rsa.locus.3859
rsa.locus.5841
cra.locus.18409
cra.locus.1416
rha.scaf149.0.16
cra
rsa.locus.36909
cra|rsa
rha.scaf517.3.16
rha.scaf517.2.14
rha.scaf517.2.15
rha
rsa.locus.8182
cra.locus.579
rsa
cra.locus.6184
cra.Genbank
rsa
cra.locus.4336
cof.03.g14110
cof.01.g02610
cof.00.g02640
cof.00.g02650
cof.05.g06770
cof
rha
cra.locus.10777
rsa.locus.1138
cra.locus.3274
rsa.locus.379
rsa.locus.379
cra.locus.1227
cra.locus.1227
cof
cra|rsa
rha.scaf517.3.7
cof
rha.supscaf18.12.34
rsa
cra.loc us.12742
rha
rsa.loc us.1084
cra.loc us.5576
1.29
Duplications : 26 - 1
Losses : 14

g.VS

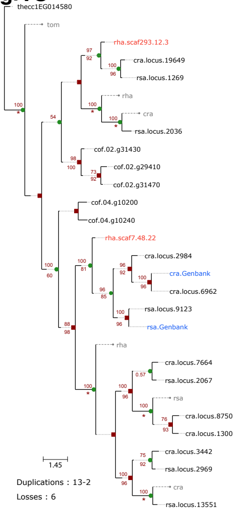

Duplications : 13-2

Losses : 6
